# Targeting α-Synuclein for PD Therapeutics: A Pursuit on All Fronts

**DOI:** 10.3390/biom10030391

**Published:** 2020-03-03

**Authors:** Margaux Teil, Marie-Laure Arotcarena, Emilie Faggiani, Florent Laferriere, Erwan Bezard, Benjamin Dehay

**Affiliations:** 1 Univ. de Bordeaux, Institut des Maladies Neurodégénératives, UMR 5293, F-33000 Bordeaux, France; margaux.teil@u-bordeaux.fr (M.T.); marie-laure.arotcarena@u-bordeaux.fr (M.-L.A.); emilie.faggiani@u-bordeaux.fr (E.F.); florent.laferriere@u-bordeaux.fr (F.L.); erwan.bezard@u-bordeaux.fr (E.B.); 2 CNRS, Institut des Maladies Neurodégénératives, UMR 5293, F-33000 Bordeaux, France

**Keywords:** Parkinson’s disease, α-synuclein, neurodegeneration, therapy, aggregation

## Abstract

Parkinson’s Disease (PD) is characterized both by the loss of dopaminergic neurons in the substantia nigra and the presence of cytoplasmic inclusions called Lewy Bodies. These Lewy Bodies contain the aggregated α-synuclein (α-syn) protein, which has been shown to be able to propagate from cell to cell and throughout different regions in the brain. Due to its central role in the pathology and the lack of a curative treatment for PD, an increasing number of studies have aimed at targeting this protein for therapeutics. Here, we reviewed and discussed the many different approaches that have been studied to inhibit α-syn accumulation via direct and indirect targeting. These analyses have led to the generation of multiple clinical trials that are either completed or currently active. These clinical trials and the current preclinical studies must still face obstacles ahead, but give hope of finding a therapy for PD with time.

## 1. Introduction

Parkinson’s disease (PD) is the second most common neurodegenerative disease, affecting about 1%–5% of the population over the age of 60 [1]. Clinically, PD is associated with motor impairments including bradykinesia, akinesia, rigidity, resting tremor and gait disturbance [2]. These symptoms are, in major part, due to the progressive loss of dopaminergic neurons in the substantia nigra (SN). In addition to this loss of dopaminergic neurons, another hallmark of PD is the presence of intraneuronal cytoplasmic inclusions, named Lewy Bodies (LB) [3], composed of the misfolded, aggregated α-synuclein (α-syn) protein, but also of other proteins and organelles [4,5]. Genetic and post-mortem evidence of PD patients undoubtedly highlight the key role of α-syn in PD pathology [6]. α-syn has also been shown to aggregate in other diseases, grouped under the term synucleinopathies. Targeting neuronal accumulation of α-syn is thus appealing to potentially halt or delay the progression of PD and other synucleinopathies.

In this review, we aimed to highlight α-syn properties that make this protein an appealing therapeutic target exclusively in the PD context. We then tried to expose the current therapeutic strategies that have been tested to target α-syn to inhibit its accumulation, aggregation or toxicity. We focused this review on discussing strategies that target specifically α-syn in preclinical and clinical studies.

## 2. α-syn: A Relevant Therapeutic Target for PD

### 2.1. α-syn Aggregation and Its Deleterious Impacts on Cellular Homeostasis

α-syn is a 14 kDa natively unfolded protein of 140 amino acids, encoded by the *SNCA* gene. This protein is highly abundant in presynaptic compartments, where it can associate with vesicles via its possible binding to membranous phospholipids [7]. It is composed of three domains: an N-terminal α-helix domain allowing lipid binding, a central non-amyloid-β component (NAC) domain responsible for its amyloid aggregation, and an unstructured flexible C-terminal domain. The protein has been shown to form a native tetramer in physiological conditions [8], but these findings are still controversial in the field [9]. In 1997, it was discovered that early-onset familial forms of PD were associated with a mutation in the *SNCA* gene [6]. That same year, α-syn was identified as the major component of LB presented by affected neurons of PD subjects [4]. These two studies shed light on this protein as a possible major actor in PD pathogenesis.

In pathological conditions, misfolded and aggregated forms of α-syn accumulate in LB. The NAC domain of α-syn confers a high propensity for the protein to misfold and to form β-sheet rich amyloid assemblies, also termed fibrils [10]. α-syn assembly into amyloid fibrils is dynamic and the existence of intermediate oligomeric species has extensively been studied. These oligomers provoked more seeding and neurotoxic effects than larger fibrillar assemblies, inferring that they might in fact be the real pathogenic species [11,12]. Deciphering the pathological relevance and exact role of these assemblies in the pathogenesis of PD is very challenging due to their reduced size, massive heterogeneity and transient nature. Recently, studies have aimed at identifying the different α-syn strains between synucleinopathies that could explain their divergent pathologies and clinical manifestations. It has been shown by several teams that there appears to be a specific aggregated form of α-syn that induces PD pathology [13,14,15].

α-syn aggregation can be considered as a stochastic event, which would increase with age and/or cell stress conditions, forming initial seed nuclei that would escape cellular clearance due to perturbed proteostasis. Increased α-syn expression and point mutations have been extensively shown to promote aggregation [16]. External factors such as viral [17] or bacterial [18] infections, and cell stress due to pesticide or toxin exposure [19,20] can also trigger the aggregation of the protein. Perturbed calcium homeostasis, mitochondrial failure, oxidative stress, and neuroinflammation are implicated in the initiation of the accumulation of α-syn as well [21,22,23].

The accumulation of intracellular aggregated α-syn shows pleiotropic pathological effects on the cell such as synaptic vesicle impairments, mitochondrial dysfunction, oxidative and endoplasmic reticulum (ER) stress, or dysfunction in the clearance pathways [24] which in turn contribute to neurodegeneration. The physiological interaction of α-syn with vesicles [25] and its role in vesicle trafficking via interactions with the SNARE complex may explain the disruption of this complex and of synaptic vesicle motility by α-syn oligomers associated with increased dopamine (DA) release [26,27]. A synaptic loss-of-function of normal α-syn, which could be triggered by its abnormal accumulation, has been shown to induce perturbations in dopamine release [28,29]. Conversely, increased levels in oxidized dopamine also impact the formation of oligomeric α-syn species and lysosomal activity [30,31]. In addition, misfolded α-syn aggregates can associate with ER membrane and cause a morphologic dysfunction, perturbations in ER chaperone levels, resulting in increased reactive oxygen species (ROS) levels, or calcium leakage to the cytosol [32]. The transport of proteins from the Golgi apparatus to the ER can also be affected by α-syn aggregates, increasing the ER stress and leading to a lack of function of protein production and quality control [33]. Mitochondrial swelling and depolarization combined with an accelerated release of cytochrome c and calcium dyshomeostasis have also been observed in vitro due to α-syn oligomers [34]. α-syn aggregates could also lead to an increase in oxidative stress by disturbing mitochondrial respiration via inhibition of mitochondrial complex I subunits [35], increasing the production of ROS by NADPH oxidase [36] or failure in antioxidant proteins [37]. These pathological assemblies are also capable of interacting with membrane lipids, thus inducing lysosomal membrane permeabilization and compromising autophagic function [38]. Lastly, it has been shown that aggregated α-syn can activate microglial inflammation, in turn participating in the induction of cell death [39]. Altogether, these various toxic effects of aggregated α-syn contribute to the loss of cellular homeostasis, therefore contributing to neurodegeneration.

In parallel, disruptions in cellular functions could also have a crucial role in α-syn toxicity towards neurons in PD. A dysfunction in α-syn clearance can lead to the abnormal accumulation of the protein, which in turn can disrupt autophagic-lysosomal and ubiquitin-proteasomal functions, entering in a vicious circle [40]. In addition, metal dyshomeostasis within the cell has been shown to impact α-syn by itself and its aggregation, via the metal binding sites present on α-syn [41,42,43].

Altogether, these findings reveal the tight links between α-syn aggregation and cellular dysfunctions in PD pathogenesis, but do not decrypt which is the trigger and which is the consequence.

### 2.2. The Prion-Like Hypothesis as a Model of α-syn Pathogenicity

The prion-like hypothesis of α-syn was raised in 2003 when six different neuropathological stages of PD were classified based on the spreading of α-syn deposits throughout affected brain areas [44]. Given that the successively affected regions are interconnected, they hypothesized the prion-like propagation of PD: the Lewy pathology would appear at the brainstem or olfactory bulb, for still unidentified reasons, and expand spatiotemporally to the neuronal connectome. Even if still under debate, this hypothesis was further strengthened in 2008 with the discovery of the development of Lewy pathology in fetal neurons grafted in the brains of PD patients more than a decade before their death [45,46,47], highlighting a potential cell-to-cell propagation of pathological α-syn.

Evidence showing that α-syn can transfer from a donor cell to a recipient cell was demonstrated in vitro using neuroblastoma cells [48] and in vivo when the transfer of α-syn to grafts in 6-OHDA-lesioned rats was observed [49], confirming the seminal findings of α-syn transfer from host to graft. These processes have since then been intensively described, with the demonstration that α-syn assemblies can be taken-up by neurons [48,50,51] via binding to the cell surface [52], or interaction with protein partners [53,54,55]. Once inside the neuron, aggregated α-syn has been shown to be directed to the lysosomal compartment [50] and can be transported along the axon [56,57]. The intracellular aggregates can, in the end, be transmitted to neighboring cells via several non-exclusive possible routes: exported extracellularly [56,58], in exosomes [59], or simply released after the affected neuron death.

This prion-like propagation of aggregated α-syn was further demonstrated following intrastriatal inoculation of recombinant α-syn pre-formed fibrils (PFF) in WT mice. This PFF injection induced the formation of α-syn-positive cytoplasmic inclusions in neurons of the striatum as well as interconnected regions such as the cortex, associated with a loss of dopaminergic neurons in the SN and motor dysfunctions [60], while a similar injection of soluble α-syn had no effect [61]. PFF also induced a progressive synucleinopathy after intracerebral injection into susceptible transgenic mice [62,63,64], and in non-human primate models [65]. In 2014, LB-enriched fractions purified from PD brains containing pathological aggregated α-syn were injected unilaterally in the SN and striatum of WT mice and non-human primates. This resulted in the induction of a progressive accumulation of pathological and aggregated forms of α-syn in nigral neurons and interconnected brain regions, leading to nigrostriatal neurodegeneration [66]. These pathogenic effects were abolished when injections were performed into mice lacking α-syn expression, or when inoculates were experimentally deprived of α-syn. The propagation of α-syn has also been demonstrated from the gut to the brain by injections of PFFs in the gut, which did not occur in α-syn knockout mice [67].

In addition to its ability to aggregate and propagate, α-syn has shown the capacity to seed the formation of new aggregates. The exposure of cells to exogenous α-syn fibrils has been shown to induce the accumulation of aggregated endogenous α-syn in many independent studies [68,69]. In these studies, fluorescently labelled α-syn exogenous fibrils were found to colocalize with newly formed endogenous α-syn aggregates [70]. The exposed cells developed a synucleinopathy resembling the one found in PD affected neurons, with the formation of cytosolic inclusions and Lewy Neurites (LN), containing pathological phosphorylated α-syn at the serine residue position 129 (pS129). These findings show that exogenous aggregates of the protein are able to recruit endogenous α-syn monomers and serve as templates in their seeded aggregation.

Altogether, these models have shed light on the capacity of α-syn to form aggregates that can be transferred from an affected cell to a naive one and induce the recruitment and seeded aggregation of the endogenous protein, leading to the expansion of the pathology. These events, recapitulated under the term “prion-like propagation”, highlight the implication of α-syn and its aggregation in the pathogenesis of PD.

All the above-mentioned findings and models undoubtedly show the central implication of α-syn in the expansion of PD pathology, leading to the disruptions and impairments of physiological cellular processes, giving rise to a progressive neurodegenerative disorder. There are currently no curative therapies for PD. In line with the pathogenic mechanisms linked to α-syn mentioned above, global effort has been focused on: (1) reducing α-syn expression and synthesis, (2) inhibiting its aggregation or reducing its accumulation through post-translational modifications, immunotherapy, and anti-aggregative molecules, (3) enhancing the degradation of α-syn as possible therapeutic strategies for PD, (4) modulating oxidative stress via anti-oxidative molecules or metal alteration.

## 3. Reducing α-syn Synthesis

The discovery of seven missense mutations, A53T, A30P, E46K, H50Q, G51D, A53V and A53E [6,71,72,73,74,75,76,77], as well as duplication and triplication [78,79] of the *SNCA* gene encoding for α-syn as a cause of several PD familial cases undoubtedly rises the interest of the field towards *SNCA* gene targeting. Moreover, the association between α-syn expression levels and the severity of the pathology [80,81] in sporadic and familial cases, strongly reinforced the link of *SNCA* gene expression to the pathophysiology of PD. Silencing *SNCA* or normalizing its levels of expression has thus been deeply explored as an appealing genetic-based therapeutic approach for PD.

The discovery of RNA interference (RNAi) paved the way to reduce *SNCA* expression level in vitro and in vivo. RNAi is a conserved process by which the double-stranded RNA targets a specific sequence of mRNA resulting either in the degradation of the targeted mRNA by the RISC complex when using short hairpin (sh)RNA, or in its translational inhibition when using small interfering (si)RNA [82,83]. Both RNAi strategies have been used in the last decades in an attempt to reduce α-syn levels through *SNCA* silencing, aiming to delay or prevent dopaminergic cell loss. In 2006, it was first shown that a lentiviral-mediated delivery of shRNA targeting human *SNCA* was efficient to silence *SNCA* expression in vitro and in rat brains when it was co-expressed with the human *SNCA* gene [84]. That same year, successful down-regulation of *SNCA* gene expression in vivo occurred using an Adeno-Associated Virus (AAV)-based approach. They were able to prevent nigral dopaminergic cell loss by injecting hammerhead ribozyme-mediated inhibition of α-syn into MPP^+^-treated adult rats [85]. Another delivery method demonstrated that stabilized naked siRNA against *SNCA* gene infused into mice hippocampi was resistant to endo- and exonuclease activity and led to efficient knockdown of *SNCA* locally (up to 70%) that lasted for up to three weeks post-infusion [86]. In addition to RNAi strategies, two microRNA (miR) targeting the 3′-untranslated region of *SNCA* have been identified as potential therapeutic targets to downregulate *SNCA* transcription. miR-7 was demonstrated to repress post-transcriptional *SNCA* gene expression in neurons and to contribute to protection against oxidative stress [87]. One year later, miR-7 and miR-153 overexpression reduced endogenous expression of α-syn in vitro and expression of these microRNA mirrored the *SNCA* expression in tissues, making them interesting for genetic-based approaches [88].

Despite the benefits obtained by such genetic-based therapies to reduce α-syn expression, the first demonstration of neurotoxicity induced by α-syn downregulation in WT rats raised long-lasting adverse effects [89]. In this study, they showed that intranigral unilateral injection of AAV carrying shRNA against *SNCA* in WT rats led to a rapid and progressive dopaminergic lesion in the nigrostriatal pathway, associated with pronounced amphetamine-induced behavioral asymmetry which correlated with the level of reduction of *SNCA* expression [89]. In 2012, these results were confirmed using an AAV delivery of shRNA against human *SNCA* into the dopaminergic neurons of the SN of rats ectopically overexpressing human α-syn [90]. They showed that human α-syn knockdown to undetectable levels aggravated the dopaminergic cell loss presented by this model despite behavioral motor improvement [90]. The same team tested a miR-embedded shRNA against human *SNCA* in vitro and in the same model of α-syn overexpressing rats [91,92]. They showed that the miR-30-embedded shRNA silencing vector was effective to reduce α-syn expression level in the rat SN and the striatum, and to prevent dopaminergic cell loss and motor impairment at two months post-injection [92]. Nevertheless, this new genetic tool also induced negative effects such as toxicity on dopaminergic fibers in the striatum, reduction of tyrosine hydroxylase global expression and neuroinflammation in the SN [92]. Finally, in 2016, three months after injection of shRNA against *SNCA* in four St. Kitts green monkeys, a loss of dopaminergic cells and fibers in a titer-dependent manner occurred [93]. These results highlighted a critical window of *SNCA* expression which is required to maintain dopaminergic cell viability. To directly test the effect of *SNCA* silencing on endogenous α-syn, the neurotoxicity induced by injection of shRNA targeting rat *SNCA* in the SN of adult rats was assessed. They showed that the nigrostriatal cell loss observed one-month post-injection was specifically due to the silencing of endogenous *SNCA* as no cell loss occurred in *SNCA* knockout mice. Loss of endogenous α-syn was toxic specifically for dopaminergic neurons as they described the initiation of both the innate and adaptive immune systems induced by *SNCA* silencing that finally contributed to a neuroinflammatory cascade and dopaminergic cell death [94]. They thus confirmed a critical threshold of endogenous *SNCA* silencing within nigral neurons above which neurotoxicity and neuronal death were certain. However, another study recently demonstrated that long-term reduction by 90% of α-syn expression using AAV-mediated delivery of shRNA against *SNCA* in rats during one year did not induce neurodegeneration in WT rats, observing only a slight decrease in tyrosine hydroxylase due to non-specific toxicities that were attributable to cellular transduction [95]. Altogether, most of these studies have shown the potential detrimental effects of complete α-syn knockdown, but these results remain controversial given the latest studies.

Even though the physiological role of α-syn is not yet completely understood, some evidence has pointed to synaptic functions [26], which could explain such adverse effects after long-lasting downregulation of *SNCA* gene. Genetic-based therapies required refining to efficiently reduce *SNCA* at physiological levels and to protect dopaminergic cells without neurotoxic side-effects. In 2010, this issue was highlighted when by partially suppressing α-syn expression using siRNA targeting specifically the squirrel monkey transcript [96]. In this first successful primate study, they produced a 21-base pair siRNA duplex targeting squirrel monkey *SNCA* transcript that was infused for four weeks unilaterally in the left SN of three monkeys. They demonstrated that naked si*SNCA* infusion induced a significant partial reduction of α-syn expression by 40%–50% in nigrostriatal dopaminergic neurons [96]. Despite this α-syn reduction, they did not observe any dopaminergic cell loss, phenotypic tyrosine hydroxylase alteration, neuroinflammation or significant neurochemical dysfunction in the striatum (dopamine, DOPAC and HVA levels were unchanged). They thus confirmed the absence of deleterious adverse effects with partial α-syn silencing induced by naked siRNA infusion in an animal model highly pertinent to humans, but they did not assess the efficacy of this strategy on the reduction of *SNCA* in PD neuropathology. Furthermore, α-syn knockdown using shRNA in human neuroblastoma SHSY5Y cells enhanced cell survival and resisted methamphetamine-induced neurotoxicity, reinforcing the interest of silencing α-syn expression as an effective therapy for neuroprotection in PD pathology [97]. To revive the interest of the field in genetic-based therapies, a Japanese group developed siRNA against human *SNCA* that reduced the expression of α-syn by half in PD patient’s fibroblasts carrying *SNCA* triplications, normalizing α-syn levels. Using *Drosophila melanogaster* as a PD model, they developed transgenic lines that co-expressed human α-syn and a siRNA directed against *SNCA* and showed that motor dysfunctions of flies were improved depending on α-syn reduction levels. They thus confirmed that moderate α-syn silencing, normalizing *SNCA* expression levels, was efficient to improve motor deficits in a simple model of PD [98]. A short-term reduction by 35% of α-syn expression by AAV-mediated delivery of shRNA targeting endogenous rat *SNCA* transcript was shown sufficient to prevent motor deficit and dopaminergic cell and fiber loss presented by rotenone-exposed rats [99]. This study also showed that even if dopamine release was slightly affected by this *SNCA* reduction, motor function or nigral dopaminergic integrity were not affected in WT rats. They thus brought the first proof of concept that partial reduction of α-syn levels could be effective in slowing down neuropathology in a rodent PD model. Altogether, these studies demonstrated that partial reduction of *SNCA* could be beneficial for PD, without the neurotoxicity observe with total ablation of *SNCA*.

Another strategy employed to reduce pathologic α-syn levels was based on the principle of allele-specific RNAi effectors. In familial cases, mutated forms of α-syn are responsible for the development of the pathology. Changes of a single nucleotide in the 21–23 nucleotides that composed an RNAi effector can abrogate the silencing ability of an RNA trigger. Allele-specific RNAi silencing can thus eliminate transcripts of mutated forms of *SNCA* without affecting WT transcripts. In 2011, a study succeeded in developing shRNA that discriminated between A30P mutated and WT *SNCA* transcripts in vitro [100]. This study highlighted the interest of targeting mutant-specific silencing of *SNCA* as genetic-based therapy in the case of hereditary forms of the pathology.

Despite emerging efforts to refine strategies aiming at silencing *SNCA* in vivo, this approach has not yet been developed enough to pursue clinical studies using *SNCA*-genetic-based therapies. Indeed, limitations of RNAi strategies slow down the application to humans, such as the need: (i) to use vectors to express certain RNAi, (ii) to directly inject into the brain to target local *SNCA* and (iii) to repeat this protocol chronically to enable sustained expression of genetic tools. The first major challenging point of such strategy for clinical application is thus to find safe and efficient vectors to cross the blood brain barrier (BBB) and deliver the genetic tools in a sustained way into the brain after a systemic administration. To overcome this limitation, modified exosomes were developed that expressed a brain-targeting peptide, the rabies virus glycoprotein peptide (RVG), and loaded with siRNA against *SNCA* gene in order to specifically target α-syn silencing to the brain [101]. After intravenous administration of RVG-modified exosomes containing si*SNCA*, they showed a significant decrease of α-syn mRNA in the midbrain, the striatum and the cortex of transgenic Tg13 mice model, with no toxicity observed [101]. More recently, RVG-decorated liposomes were used to deliver siRNA for α-syn silencing into mice primary hippocampal neurons as a first validated step towards in vivo systemic therapy [102]. To optimize viral vector penetration into the brain, a non-invasive magnetic resonance-guided focused ultrasound was tested combined with systemic injection of microbubbles to locally and transiently increase the BBB penetrance [103]. Using this non-invasive methodology, they succeeded in silencing human *SNCA* by 60% in four mice brain regions (hippocampus, SN, olfactory bulb and dorsal motor nucleus) after systemic delivery of AAV-sh*SNCA* vectors through the tail vein of transgenic mice overexpressing human *SNCA*, with no deleterious side effects [103]. Another group proposed the use of intranasal delivery—a semi-permeable BBB region—of antisense oligonucleotide against *SNCA* conjugated with indatraline, a triple monoamine transporter blocker with differential affinities, to specifically target monoamine neurons involved in PD pathology [104]. They obtained a time-dependent knockdown of α-syn specifically in serotoninergic and dopaminergic neurons of the SN, the ventral tegmental area, the putamen, the caudate and the locus coeruleus [104]. Finally, a non-viral vector approach was developed based on the systemic injection of a small peptide derived from the envelope protein of the rabies virus (C2–9r) complexed with siRNA targeting α-syn in MPTP-mice [105]. This showed a sustainable neuronal specific knockdown of α-syn by 60%–90% associated with dopaminergic neuroprotection and motor improvement. Recently, the same group developed another peptide derived from the apoB protein in order to carry the si*SNCA* and showed equivalent efficient neuronal specific silencing of α-syn and neuroprotection after intraperitoneal injection in human-*SNCA*-overexpressed transgenic mice [106]. Even though no clinical trials exist yet, reduction of *SNCA* expression and delivery of these gene-based therapies are being developed and could be promising for PD therapeutics.

Despite all these technical advances and appealing strategies to silence efficiently and with refinement the *SNCA* gene in the brain, genetic-based therapies targeting α-syn silencing reflected the complexity of modifying gene expression in the brain, and notably in the field of PD pathology. Interestingly, the recent discovery of ZSCAN21 as a transcriptional factor for *SNCA* [107] and its upstream regulation with TRIM17 and TRIM41 [108] could be an alternative route to modulate *SNCA* gene expression. In the same line of evidence, a recent paper reported the role of β2-adrenoreceptor agonists on *SNCA* gene expression and regulation showing that β2-adrenoreceptor agonists decreased *SNCA* expression in a dose-dependent manner through Histone 3 Lysine 27 deacetylation in WT mice SN [109]. Future strategies to silence partially *SNCA* gene expression may be focused on drug candidates that may act on these new pathways in order to pave the way of genetic-based approaches targeting α-syn silencing with high translational impact for PD. Nonetheless, these genetic-based strategies still require refinement to perturb pathological forms of α-syn all the while maintaining the physiological levels of α-syn.

## 4. Targeting α-syn Post-Translational Modifications

α-syn has been shown to undergo several post-translational modifications (PTM), of which numerous are associated with the pathological forms of the proteins. These modifications, such as phosphorylation, truncation, acylation, ubiquitination, or glycation can impact pathological α-syn aggregation, or oligomer formation, with many different mechanisms, and represent possible molecular targets in the development of disease-modifying therapeutic approaches.

### 4.1. Phosphorylation

Phosphorylation of α-syn has been the most studied PTM of the protein since the finding in 2002 that α-syn is phosphorylated at residue S129 in LB [110]. Importantly, non-fibrillar forms of α-syn extracted from tissues of PD patients can also be phosphorylated at this site [111]. pS129 α-syn has since emerged as a major hallmark of the disease, as well as other synucleinopathies. Other serine and tyrosine residues of the protein can be phosphorylated, such as S87, Y125, Y133 and Y136 [112,113,114]. The exact roles of the latter in PD pathogenesis are still to be determined, as many independent laboratories have shown it to be either neuroprotective or neurotoxic. Multiple studies have shown that pS129 α-syn promotes its fibrillization in vitro [110], or accelerates the formation of inclusions and exacerbates its toxicity in cell models [115]. Other studies have shown a neuroprotective effect of this PTM, as the phosphorylation deficient mutant S129A was found to be more toxic in different animal models compared to WT α-syn [116,117]. Paradoxically, this mutant was shown to induce the formation of large inclusions, rather than—possibly more toxic—small oligomers ending with a protective effect [118,119].

In an attempt to modulate the phosphorylation of α-syn as a therapeutic approach, two molecular players in this pathway have been targeted: kinases and phosphatases. Numerous kinases phosphorylate α-syn, especially at S129, such as casein kinases [112], G protein-coupled receptor kinases [113,120], or Polo-like kinases (PLK) [121]. PLK2 was shown to phosphorylate α-syn more efficiently than PLK1 and PLK3, and this PTM was largely reduced in PLK2-/- transgenic mice [117,121]. In transfected cells expressing WT α-syn and treated with recombinant PFF, PLK2 phosphorylated α-syn under its monomeric, or aggregated forms, allowing the modulation of its phosphorylation status at different stages [122]. In this model, PLK2-induced pS129 α-syn had no effect on the aggregation of the protein, but could mediate its clearance through the autophagy-lysosomal pathway [123]. The modulation of PLK2-mediated phosphorylation was obtained by its inhibition with selective and brain-permeable chemical compound BI2536 [121,122]. In WT mice and rats, treatment with BI2536 inhibited pS129 α-syn [121,124], and could be delivered locally by nanoparticles [125] but the exact consequences on this inhibition on the pathology of PD are still to be decrypted. However, as kinases phosphorylating α-syn have ubiquitous distribution and possibly compensatory effects, the development of safe and efficient brain-penetrant compounds selectively modulating this PTM is extremely challenging.

To modulate α-syn phosphorylation, phosphatases may represent another possible therapeutic target. Despite the interest in its phosphorylation, few studies have targeted phosphatases selectively dephosphorylating α-syn. Protein phosphatase 2A (PP2A) has been shown to dephosphorylate pS129 α-syn [126], although these results remain controversial [127,128]. Recently, the antidiabetic drug metformin significantly reduced the levels of pS129, through activation of PP2A, in cells and in vivo [129,130]. In MPTP-exposed mice, activation of PP2A by metformin presented neuroprotective effects, with restored dopamine depletion and behavioral impairments [130]. This phosphatase is ubiquitous and its specificity is determined by regulatory B subunits [126]. Assembly of pS129-dephosphorylating PP2A is regulated by reversible methylation, representing a possible control mechanism [131,132]. Thus, the use of activators to increase its S129 phosphatase activity represent an interesting therapeutic approach for PD. This has been achieved in mice brains with the use of a coffee component, eicosanoyl-5-hydroxytryptamide (EHT), which, by inhibiting demethylation of PP2A, enhanced its phosphatase activity toward α-syn [126]. In a human α-syn transgenic mouse model, using an EHT-enhanced diet, S129 phosphorylation was decreased, with concomitant reduction in α-syn aggregation and improvement of neuronal integrity leading to improved motor performance. Equivalent results were obtained on a similar WT α-syn transgenic mouse model, showing a synergistic effect of caffeine and EHT in enhancing PP2A activation [133]. These mechanisms of enhancing PP2A dephosphorylation of α-syn appear as promising therapeutic approaches, as they are safe and can be specific to α-syn through an allosteric activation, therefore avoiding the deleterious effects towards off-target proteins.

### 4.2. Truncation

Numerous studies have reported the presence of different truncated forms of α-syn in LB, and about 15% of LB-contained α-syn is truncated [134,135,136]. The generation of C-terminally truncated forms of the protein has been demonstrated in vivo [137] and was shown repeatedly to promote fibril assembly and to enhance full-length α-syn propensity to aggregate [138,139,140,141]. C-terminal truncation of α-syn is required for its cellular processing and can be found in control brains [137], but truncated α-syn is present in higher amounts and within higher molecular-weight species in PD brains [140]. The underlying mechanisms of α-syn proteolytic cleavage are not yet understood, but several α-syn-cleaving proteases have been identified, and represent interesting potential targets for modulating its aggregation.

Calpain I is a calcium-dependent neutral protease, activated by increased intracellular calcium levels, therefore prone to cleave α-syn in presynaptic terminals where it is predominantly localized. In vitro, calpain I cleaved α-syn after amino acid 57, and within the NAC domain, and the former cleavage was impossible on A53T mutant α-syn [142]. Under fibrillar forms, α-syn is cleaved at amino acids 117 and 122, generating C-terminal fragments that retain their fibrillar structure and induce the co-aggregation of the full-length protein. Calpain I-mediated cleavage of soluble monomeric α-syn inhibited its fibrillization, while processing of the fibrillar form of the protein by calpain I promoted its further aggregation [143]. In PD, calpain expression levels and activity were elevated in the brain, and led to synaptic dysfunction and neuronal death by contribution to the formation of toxic oligomers [144]. This study also revealed that calpain-cleaved α-syn was found in nigral LB and LN, colocalizing with activated calpain. Recently, fibrillar α-syn C-terminal truncation mediated by calpains was shown to participate in regulating seeding, fibrillization, and LB formation and maturation in vitro [145]. The inhibition of α-syn cleavage by calpain could thus represent an interesting therapeutic option. The effects of calpastatin, a natural calpain-specific inhibitor, were demonstrated in vivo, in double transgenic mouse models either overexpressing both human mutated A30P α-syn and human calpastatin showing reduced calpain activity, or overexpressing A30P on a calpastatin-deficient background associated with increased calpain activity [146]. In these animal models, calpastatin-induced reduction of calpain activity led to decreased α-syn aggregation, while the loss of calpastatin led to increased truncation of α-syn. Furthermore, overexpression of calpastatin ameliorated the neuropathology observed in A30P mutant mice. In a recent study, the effects of systemically administered low molecular weight calpain inhibitors, Neurodur and Gabadur, on α-syn were demonstrated in vivo. In transgenic mice overexpressing human WT α-syn, increased calpain activity and α-syn cleavage resulted in its aggregation and toxicity [147]. Treatments with both calpain inhibitors resulted in the reduction in α-syn deposits, with a reduction in its C-terminal cleavage, and improvement in neurodegeneration and activity performance of these mice.

Caspase I was also shown to truncate α-syn in vitro, which generated highly aggregation-prone species. This truncation-induced aggregation was toxic to neuronal cultures, and the specific inhibition of caspase I by the chemical compound VX-765 improved neuron survival [148]. In addition, neurosin, a serine protease predominantly expressed in the central nervous system, previously detected in LB, inhibited α-syn fibrillization in vitro and was involved in its degradation [149] through α-syn cleavage within the NAC region [150], therefore inhibiting its aggregation. Lastly, similarly to calpain and neurosin, α-syn C-terminal cleavage has been shown to be mediated by cathepsin D [151] or matrix metalloproteinase 3—which were also detected in LB—[152,153], but their respective roles in truncating α-syn intracellularly in vivo remain unclear, rendering their inhibition challenging in therapeutic development for PD. Taken together, inhibiting truncation of α-syn has been shown to prevent fibrillation and aggregation, making it an interesting potential therapeutic target.

### 4.3. O-GlcNAcylation

The *O*-linked β-*N*-acetyl glucosamine (*O*-GlcNAc) modification is a dynamic glycosylation in which the uncharged acetylated hexosamine sugar N-acetylglucosamine (GlcNAc) is added and removed to the serine or threonine residues of proteins by intracellular enzymes *O*-GlcNAc transferase and O-GlcNAcase respectively [154]. α-syn has been shown to be O-GlcNAcylated at nine different threonine residues in proteomics experiments from mouse and human tissues [155,156,157]. Most of these modifications are located in the NAC region, with the T72 O-GlcNAcylation appearing as particularly important. Indeed, the addition of a single O-GlcNAcylated residue at T72 completely inhibited the oligomerization and fibrillization of the full-length unmodified protein, without affecting its ability to bind membranes, and prevented the toxicity of α-syn in the treatment of neuronal cultures [158]. S87 O-GlcNAcylation was also shown to decrease the aggregation of the protein [159]. Both T72 and S87 O-GlcNAcylation inhibit the calpain-mediated cleavage of α-syn, associated with its aggregation [160]. It was shown recently, through the synthesis of six site-specific O-GlcNAcylated α-syn variants, that O-GlcNAcylation in general inhibited the aggregation of the protein, and could also alter the structure of its aggregated forms [161]. In this study, several of the O-GlcNAcylations prevented the toxicity of extracellular α-syn fibrils in a primary neuron culture PD model. Additionally, O-GlcNAcylation inhibited the aggregation of an aggressive α-syn mutant. Interestingly, MK-8719, a CNS penetrant O-GlcNAcase inhibitor towards tauopathies, has been advanced to phase 1 clinical trial [162]. This molecule could also represent a promising anti-aggregative therapeutic target towards α-syn in PD.

### 4.4. Other PTMs: Ubiquitination, SUMOylation, Acetylation, Glycation and Nitration

As many other cellular inclusions associated with neurodegenerative disorders, ubiquitylated proteins are components found in LB [163]. α-syn itself in LB is mainly mono-, di-, and tri-ubiquitinated [164,165,166]. A recent study showed that ubiquitin-specific protease 13 (USP13) can regulate α-syn clearance [167]. This de-ubiquitinase was found to be upregulated in post-mortem PD brains. Furthermore, knocking down USP13 resulted in increased α-syn ubiquitination and clearance in WT mice, prevented neuronal death and improved motor performance in α-syn lentiviral or transgenic PD mouse models. The major issue with targeting these ubiquitinating ligases resides in the lack of specificity of these pathways, and on the opposite effects of ubiquitination on α-syn aggregates [168,169,170,171,172,173,174,175].

α-syn has also been shown to be SUMOylated at lysine residues by PIAS2. An increase in PIAS2 expression along with SUMOylated α-syn were detected in PD brains, and PIAS2 was even detected in LB within nigral neurons. SUMOylation by PIAS2 directly promoted the aggregation of α-syn, and impaired its ubiquitination by the above cited ligases, preventing its degradation [176]. Importantly, the SUMOylation of α-syn was also shown in other studies to inhibit its aggregation and toxicity [177,178]. Thus, SUMOylation could be an interesting target but, given the inconsistencies observed, more research must be done on this PTM.

Acetylation of α-syn has also been demonstrated at several Lysine residues, predominantly in the N-terminus (K6 and K10). This acetylation at the N-terminus stabilized the helical structure of α-syn, increasing its binding to the membrane. In addition, acetylated α-syn led to a lower propensity to aggregate than non-acetylated α-syn, but also to a different fibril polymorphism [179]. Sirtuin 2 (Sirt2), a deacetylase, has been implicated in α-syn mediated toxicity and aggregation in *Drosophila melanogaster* [180]. More recently, α-syn acetylation at K6 and K10 modulated not only aggregation but also autophagy in vitro. In this same study, Sirt2 knockout mice injected with either AAV-α-syn or MPTP showed less dopaminergic cell loss [181]. Altogether, these studies elucidate the potential therapeutic effects of Sirt2 targeting to decrease α-syn aggregation.

Finally, glycation of α-syn was first shown to be implicated in its toxicity through DJ-1, which has both glyoxalase and deglycase activities [182]. The increased glycation on α-syn Lysine residues through the use of methylglyoxal (MGO) induced an increase in α-syn toxicity and aggregation in vitro and in vivo. MGO was shown to amplify α-syn oligomer production and interfere with the N-terminal structure of the protein. Using anti-MGO molecules such as aminoguanidine and tenisetam, this same study demonstrated a better α-syn clearance and lower aggregation in *Drosophila melanogaster* [183]. Glycation inhibition via MGO scavengers constitute potential novel possibilities to lower α-syn oligomer formation.

Lastly, nitration of all four tyrosine residues of α-syn have been described [184,185,186]. But this PTM has shown very diverse effects on the aggregation of the protein [186,187,188] and therefore is not yet a viable therapeutic target in the treatment of PD.

In summary, targeting α-syn PTM in the development of treatments for PD can represent an interesting therapeutic approach. However, we have to bear in mind that the exact roles of these protein modifications in the pathogenesis of the disease are not yet fully unraveled. In addition, the enzymes that must be targeted to modify these PTM do not act specifically on α-syn, so they could be deleterious for the function of other proteins. Modifying these enzymes must thus be precise to only modify α-syn PTM.

## 5. Immunotherapy

Immunotherapy relies on the boost of the immune system as a potential therapy. In the case of PD, it relies on the immune system being directed towards the elimination of α-syn aggregates. There exist different types of immunizations: passive, active and alternative.

### 5.1. Passive Immunization

Passive immunization relies on the use of antibodies directed towards α-syn protein sequence to protect against neurodegeneration and reduce α-syn accumulation and propagation. This strategy presents many unknowns, including the fact that antibodies must be able to recognize α-syn despite its cytoplasmic localization and pass the BBB. Nonetheless, the propagation and abnormal localization of α-syn could make passive immunization relevant as a therapeutic strategy. Two main regions have been used to target α-syn: the C-terminal and the N-terminal regions of this protein.

The 9E4 antibody was one of the first antibodies tested in transgenic mice overexpressing human WT α-syn that showed beneficial effects [189]. After six months of 9E4 injections, the antibody had indeed entered the CNS and localized in lysosomes. These mice showed an improvement in their motor and learning deficits, as well as a reduction of α-syn accumulation in synapses and axons of cortical and hippocampal neurons [189]. Following this positive study, this murine antibody was derived to create the first antibody used for human clinical trials under the name PRX002. Combined, two phase 1 clinical trials showed promising results in the safety and tolerance of PRX002 in healthy volunteers and PD patients, and its potential to eliminate free α-syn [190,191]. Currently, phase 2 trials for this antibody are underway (NCT03100149). Targeting the C-terminus of α-syn using antibodies was also tested using α-syn transgenic mice. Weekly administrations of the antibody named 274 revealed its beneficial effects on α-syn clearance. The interaction of 274 antibody with α-syn formed complexes which were then internalized by microglia via Fcγ receptors. Injection in vivo of 274 decreased cell-to-cell transfer of α-syn, reduced accumulation of α-syn, and increased α-syn uptake and clearance by microglial cells [192]. The third group of antibodies targeting the α-syn C-terminus, 1H7, 5C1 and 5D12, were tested in transgenic mice overexpressing WT human α-syn. 1H7 and 5C1 antibodies were able to reduce accumulation of cortical and striatal α-syn, neurodegeneration and behavioral alterations in these mice. The authors determined that these antibodies were able to block α-syn truncation by calpain I, inhibiting propagation and accumulation of α-syn, and thus its neurotoxicity [147]. Following these results, a combined model of transgenic mice overexpressing WT α-syn and lentiviral α-syn unilateral injection was used to assess the effect of immunization with the 1H7 antibody. Injections of 1H7 reduced α-syn-induced neurodegeneration, motor impairment, as well as axonal accumulation and transport to the contralateral hemisphere after the lentiviral injection [193]. Recently, a fourth antibody targeting the C-terminus of α-syn has been proven to sequester extracellular α-syn and to decrease spreading in vitro and in vivo: MEDI1341. MEDI1341 was capable of blocking α-syn accumulation and its trans-axonal spreading in hippocampi of mice unilaterally injected with human α-syn [194]. MEDI1341 is currently in a single-ascending dose phase 1 human clinical trial in healthy volunteers to determine its safety and tolerability (NCT03272165).

Concerning antibodies targeting the N-terminus, α-syn monoclonal antibodies were tested in PFF-injected mice to determine whether immunization could block PFF entry, cell-to-cell propagation and neurodegeneration. Syn303 was capable of recognizing and blocking misfolded α-syn in vitro, and was then tested in PFF-injected mice. Syn303 treatment reduced and delayed the spread of α-syn pathology, improved motor performances, and inhibited dopaminergic cell loss compared to non-treated PFF mice [195]. In another study targeting the human N-terminus of α-syn, intraperitoneal injection of the AB1 antibody in an AAV-WT α-syn rat PD model was able to protect dopaminergic cells, but was not completely able to protect against the motor deficits. In addition, AB1 reduced levels of α-syn and microglial activation in the SN [196]. Recently, another human-derived antibody (BIIB054) targeting the N-terminus was capable of binding aggregated forms of α-syn. This study showed the ability of this antibody to bind to pathologic α-syn in different mice models (PFF-injected and AAV-A53T human α-syn mice) as well as in post-mortem PD human brains. In mice injected with PFFs, treatment using BIIB054 was able to reduce the loss of dopamine transporter DAT in the striatum, decrease α-syn spreading, and alleviate motor impairments [197]. This antibody has already been implicated in a phase 1 clinical trial to test its safety and tolerance in healthy and PD participants. Single-dose intravenous injections up to 130 mg/kg were tested and relatively well tolerated in healthy participants, with similar adverse effects for all doses up to 45 mg/kg. PD patients then received a single dose up to 45 mg/kg, which was well tolerated, and displayed pharmacokinetic profiles that were similar to healthy volunteers [198]. This encouraging phase 1 data supports the start of the phase 2 trial of BIIB054 (NCT03318523).

In addition to antibodies targeting the specific sequence of α-syn, certain antibodies are being developed to specifically target oligomeric or fibrillary pathogenic forms of α-syn [199,200,201,202]. The mAb47 antibody was shown to target specifically α-syn protofibrils in human A30P α-syn transgenic mice. Administrations of mAb47 proved to specifically reduce the amount of protofibrils, without affecting monomeric or fibrillary α-syn, and decreased the motor deficits present in this model [201]. Based on these results, the BioArctic company has developed an antibody targeting human α-syn derived from mAb47, BAN0805, which is currently being tested in phase 1 clinical trials (NCT04127695). Finally, five antibodies specific to oligomeric and fibrillary α-syn were developed and used to immunize mice weekly for over 3 months. Immunization with Syn-F1, Syn-O1 and Syn-O4 decreased accumulation of α-syn oligomers, reduced PK-resistant α-syn aggregates, prevented neurodegeneration in the hippocampus, and improved behavior of WT α-syn overexpressing mice. Additionally, only Syn-O4 was able to reduce the activation of microglia [202]. Taken together, the last decade has seen the rise of passive immunization as a potential lead in the treatment of PD. Multiple promising phase 1 and 2 clinical trials are currently underway and many preclinical studies could give rise to other trials as well.

### 5.2. Active Immunization

Active immunization or vaccination relies on the activation of the immune system to generate antibodies targeting a specific antigen. In the case of PD, the goal is to target α-syn to potentially reduce its aggregation and propagation in patients in the long-term.

In 2005, a first study aimed at using active immunization in WT α-syn transgenic mice by injecting mice with purified full-length recombinant α-syn expressed in *E. Coli*. After immunization, human α-syn antibodies generated in mice were able to decrease the accumulation of α-syn in neurons and neurodegeneration in synapses. Given the colocalization of α-syn with the lysosomal marker cathepsin D, the immunized mice could activate degradation pathways and thus eliminate α-syn more efficiently [203]. Following this study, another vaccination approach fused α-syn epitopes (α-syn_85–99_, α-syn_109–126_, α-syn_126–140_) with P30, a T-helper epitope from tetanus toxin, to avoid the activation of harmful T-helper responses after vaccination. These three α-syn epitopes fused with P30 were injected in WT mice, which induced a strong antibody response. The antibodies produced by this response were in turn able to recognize LB and LN in post-mortem brain samples [204].

Other studies have tried active immunizations using small peptides, or Affitopes. Affitopes are short peptides with a sequence mimicking the native epitope, which are capable of eliciting a B-cell response without activating T-cell responses. These peptides have already been tested in AD successfully and have reached clinical trials. AFF1 was used to target the C-terminus of α-syn in WT α-syn transgenic mice. Immunization with AFF1 promoted the clearance of aggregated α-syn without affecting murine monomers or total α-syn. In addition, AFF1 was able to ameliorate motor functions, reduce neurodegeneration, decrease inflammation of microglia and astroglia, and promote secretion of anti-inflammatory cytokines [205]. Following these positive results of AFF1, Affitopes PD01 and PD03 were developed for human clinical trials. In 2012, the first phase 1 clinical trial for PD01 including early-stage PD patients and healthy volunteers demonstrated low toxicity for both subcutaneously injected doses that were tested. This was followed by a boost immunization in participants, which was also well tolerated and participants are currently being observed over time. First immunizations with PD01 induced an immune response against the peptide and against the targeted α-syn epitope, with generation of α-syn antibodies. The boost immunization then showed an increase in reactivity in the immune response [206,207,208].

Recently, combining cellular and humoral immunization using a glucan particle (GP) vaccine containing both an α-syn antigen and rapamycin has also been tested in WT α-syn transgenic mice. This GP vaccine induced a strong immune response with activation of T-regulatory cells in the CNS as well as a strong α-syn antibody production. This in turn reduced α-syn accumulation, neuron loss, and microglial activation, which could not be observed without the combined immunization [209].

The pursuit of active immunization in the case of PD has been growing quickly in the last 15 years, in particular due to its large-scale implications if successful. Nonetheless, vaccination remains difficult due to the fact that the antibodies generated must find their way to the CNS in large enough quantities to have a substantial effect.

### 5.3. Alternative Immunizations

Other than passive and active immunizations, alternative immunizations have arisen where α-syn is not targeted directly by antibodies. Dendritic cell (DC)-based vaccination relies on the use of antigen-sensitized dendritic cells as vehicles for immunization. A53T α-syn transgenic mice were immunized with different α-syn peptide-sensitized DC vaccines over 17 months to assess their effect on α-syn antibody production and α-syn clearance. Results indicated that peptide-sensitized DC vaccines were able to induce a humoral response and generate α-syn antibodies. They were also able to decrease the levels of pro-inflammatory cytokines and partially protect from locomotor defects [210].

Aptamers are short, single-stranded DNA or RNA molecules that have been used as an alternative to antibodies as they are not immunogenic or toxic and, given the fact that they are composed of nucleic acids, they are extremely stable in the organism. Recently, aptamers targeting α-syn have been developed to inhibit α-syn aggregation and rescue cellular functions associated with its accumulation [211]. After successful tests in vitro, two aptamers were tested via RVG-exosome delivery in PFF-injected mice. After injections of the aptamer-containing exosomes, mice showed a decrease in dopaminergic cell loss and in α-syn accumulation in the midbrain. In addition, motor symptoms were reduced, but no changes were observed in microglial inflammation [212]. This was the first study that successfully delivered aptamers to the CNS and reduced the accumulation of α-syn.

Immune Toll-like Receptor-2 (TLR2) has previously been implicated in synucleinopathy in multiple capacities: astrogliosis, cell-to-cell transfer of α-syn, and autophagy-mediated clearance [213,214]. In this respect, TLR2 appears to be a potentially interesting target to decrease α-syn accumulation and propagation, via its roles in autophagic clearance and cell-to-cell transfer. To target TLR2, TLR2-depleting antibody (T2.5) was used in WT α-syn transgenic mice. After treatment, mice showed a decrease in α-syn aggregation, astrogliosis and microgliosis, and neuronal loss [215]. This suggests a potential alternative immunization by targeting α-syn accumulation and propagation indirectly.

Finally, intrabodies are small 14–30 kDa proteins that are derived from antibody fragments and are engineered to act intracellularly. Intracellular antibodies, unlike classic antibodies, are composed only of the Fv variable regions which determine the specificity of the antibody. Different intrabody structures exist: single-chain Fv intrabodies, with both the variable heavy and light chain, single domain intrabodies, or camelid nanobodies, with small heavy-chain-only fragments [216]. Multiple studies aiming to target α-syn via intrabodies have reported efficiency in vitro and with computational modeling [217,218]. Recently, VH14 and NbSyn87 intrabodies, targeting the NAC region and C-terminus of α-syn respectively, have been shown to interfere with α-syn aggregation in vitro [219,220]. VH14 and NbSyn87 combined with the PEST motif that targets the ubiquitin-proteasome system, have additionally been shown to block α-syn accumulation and attenuate proteasomal stress [221,222]. VH14*PEST proved to be more successful in targeting α-syn accumulation and showed very little neuroinflammation, demonstrating its potential for future potential therapies.

To conclude, immunotherapy targeting α-syn has been extensively studied using passive, active or alternative immunizations. Independently of the type of immunization, it appears that it could be feasible to reduce the amount of both intracellular and extracellular α-syn. Additionally, self-made antibodies could also inhibit the cell-to-cell propagation of α-syn. This could have extremely beneficial effects in avoiding spreading of α-syn, and thus neurodegeneration and neuroinflammation. In addition, complete immunization would require several injections over a long period of time, but the procedure is relatively non-invasive for patients. Nonetheless, it is important to note the disadvantages that accompany immunization. Little is also known on the effect of producing such large amounts of soluble α-syn on the native and pathologic forms of the protein, nor the localization of the protein. To successfully target α-syn, these immunizations rely on targeting a specific type of oligomer or aggregate, which would restrict the immunization and omit other forms of oligomers/fibrils. Also, LB contain different forms of α-syn, modified by PTM and truncated forms, which could also reduce the efficiency of these α-syn antibodies. Furthermore, we have yet to determine the immune reaction that could occur in the long-term against these α-syn antibodies and repeated injections. Certain strategies such as nanobodies and Affitopes attempt to avoid this immune activation, but the reaction to other strategies remains to be seen. Finally, despite extensive studies in animal models, a method to measure the penetrance and efficacy of α-syn-targeting treatments has yet to be discovered. Measuring the CSF and serum levels of α-syn have not yet been completely refined nor been proved to correlate with brain α-syn levels [223]. Without this measure in vivo, it seems difficult to determine the actual efficiency of the treatment.

## 6. Anti-Aggregative Small Molecules

Several small molecules have been discovered or developed by drug screening or drug repositioning to treat PD in the goal to provide an anti-aggregative strategy. Several mechanisms of action have been reported: direct interaction with α-syn by small molecules or indirect modulation of chaperones to inhibit α-syn aggregation.

### 6.1. Molecules Directly Interacting with α-syn

Among the existing anti-aggregative small molecules, some have the ability to directly interact with α-syn and have been explored for their ability to slow accumulation of α-syn, making them potential therapeutic targets for PD.

Certain small molecules have been developed to specifically target Lysine residues of α-syn. Molecular tweezers inhibit key interactions in the self-assembly of amyloidogenic protein by binding to positively charged amino acid residues and disrupting both hydrophobic and electrostatic interactions [224,225]. The molecular tweezer, termed CLR01, was demonstrated as an inhibitor of aggregation of α-syn into fibrils in cell culture and zebra fish embryo. It could also disaggregate de novo fibrils by binding specifically with the lysine residue of α-syn via the hydrophobic and electrostatic forces [226]. CLR01 was shown to bind multiple lysine residues and potentially modified the kinetically controlled aggregation process [227]. More recently, continuous intracerebroventricular administration of CLR01 in WT α-syn transgenic mice improved motor dysfunction and caused a significant decrease of soluble α-syn in the striatum [228]. In 2005, DA and its analogs were also showed to inhibit α-syn fibrillization. More specifically, DA oxidation products, termed quinones, interact with lysine residues of α-syn, leading to the inhibition of α-syn fibrilization [229]. Moreover, in vitro fibrilization assays were performed and revealed that five selected dopamine analogs affected the aggregation process [230]. Recently, developing an assay to screen compounds with α-syn modulating properties, dopamine agonists D-519 and D-520 were discovered and showed to modulate aggregation and toxicity of α-syn. They notably showed its neuroprotective effect against the toxicity caused by α-syn in a *Drosophila melanogaster* model of synucleinopathy [231]. Analyzing α-syn species by biochemical approaches, another small molecule, an *ortho*-iminoquinone (IQ) was shown to reduce amyloid aggregation by reacting with lysine residues. IQ also reacted with free amines within the amyloid fibrils preventing their dissociation and seeding capacity [232].

Another group of small molecules preferentially bind to the C-terminal region of α-syn. Among them, the protein endosulfine-alpha (ENSA), a member of the cAMP-regulated phosphoprotein family, has been reported to interact specifically with membrane-bound α-syn [233,234]. The interaction between ENSA and α-syn led to an inhibition of membrane-induced α-syn aggregation and ENSA overexpression decreased α-syn neurotoxicity in neuronal cultures [235]. In the same line of evidence, the isoquinoline derivative Fasudil is the first small molecule Rho-associated protein kinase (ROCK) inhibitor developed for clinical use in humans. Using cell culture and cell free assay, the anti-aggregative potential of Fasudil was revealed, through its effects mediated by ROCK-inhibition, binding to tyrosine residues Y133 and Y136 in the C-terminal region of α-syn. Furthermore, long term treatment improved motor and cognitive functions and significantly reduced the α-syn pathology in the midbrain in the A53T α-syn transgenic mouse model [236]. Finally, despite its lack of metabolic stability and low oral bioavailability, previous studies demonstrated promising beneficial effects of NPT100-18A, an α-syn misfolding inhibitor, in vitro and in α-syn overexpressing transgenic mice [237]. NPT200-11, an optimized compound, with pharmacokinetic properties suitable for clinical evaluations, maintained robust beneficial actions in α-syn-based animal models. NPT200-11 was shown to interact with a domain in the C-terminal region of α-syn, thus reducing α-syn pathology, neurodegeneration and CNS inflammation and improving behavior impairment in transgenic mice that overexpressed WT α-syn or α-syn linked to GFP [238]. A phase 1 clinical trial for this molecule testing its safety and tolerability has already been completed, and another phase 1 clinical trial to test its safety in PD patients is planned (NCT02606682).

Finally, based on a systematic high-throughput screening campaign combined with medicinal chemistry optimization, the oligomer modulator anle138b [3-(1,3-benzodioxol-5-yl)-5-(3bromophenyl)-1H-pyrazole] was developed. In vitro and in two different PD mice models, anle138b blocked the formation of pathological aggregates of α-syn targeting specifically oligomeric forms. In both rotenone-exposed and A30P α-syn transgenic mice, it strongly inhibited oligomer accumulation, neuronal degeneration [239], and disease progression even if the treatment started after disease onset [240]. Recently, anle138b rescued the dopamine deficit and reduced the density of α-syn aggregates associated with an increase in dispersed monomeric and small assemblies in transgenic mice brains [241]. This promising molecule for PD therapy, with high bioavailability and low toxicity, is currently in phase 1 of a clinical trial started at the end of 2019 (NCT04208152).

### 6.2. Heat Shock Protein Modulators

Heat shock proteins (Hsp) are molecular chaperones that assist in proper conformational binding of proteins, including α-syn [242]. It was shown that α-syn co-immunoprecipitated with Hsp90 and Hsp70 [243] and that Hsp modulators were protective against α-syn-induced toxicity, and could prevent its aggregation. In protein quality control processes, Hsp90 and Hsp70 had opposing effects on target protein stability. Hsp90 stabilized the proteins and inhibited their ubiquitination, whereas Hsp70, along with its co-chaperone Hsp40, was required for the degradation of many proteins promoting ubiquitination and proteasomal degradation dependent of CHIP, a component of LB in the human brain [168]. Modulating proteostasis by inhibiting Hsp90 function or by promoting Hsp70 function enhanced the degradation of the critical aggregating proteins and could be used for the treatment of PD against α-syn toxicity [244].

The first compound that was investigated in PD models was Geldanamycin, a naturally occurring antibiotic of the Ansamycin family. Geldanamycin effectively prevented α-syn aggregation by increasing its clearance, leading to a reduced toxicity in yeast model of PD, expressing WT or A53T α-syn [245]. A *Drosophila melanogaster* model of α-syn toxicity confirmed the decrease of α-syn oligomerization associated with neuroprotective effects of Geldanamycin [246,247,248]. This protective effect was also showed in the MPTP mouse model of PD [249]. Mechanistically, Geldanamycin protected cells against extracellular α-syn-induced neurotoxicity by preventing re-secretion of α-syn [250]. Similarly to Geldanamycin, 17-AAG (Tanespimycin) attenuated α-syn toxicity, prevented oligomerization and facilitated α-syn clearance in cultured cells [251,252]. Despite encouraging results, the use of Geldanamycin or 17-AAG has been limited because of their poor solubility and BBB permeability. On the contrary, synthetic small-molecule inhibitors of Hsp90 such as SNX-2112 and its derivatives displayed good pharmacokinetic characteristics. Using the bioluminescent complementation assay, a decrease in both high-molecular weight and monomeric α-syn was showed, as well as a reduction of α-syn oligomerization in cell culture models treated with SNX compounds. Even if most derivatives inhibited α-syn oligomerization, the four compounds SNX-3113, SNX-3723, SNX-8891 and SNX-0723 were found most potent in this study [251]. The site of interaction between Hsp90 and α-syn affected regions that are responsible for vesicle binding and amyloid fibril assembly. Those processes are perturbed in an ATP-dependent manner. Indeed, in the absence of ATP, Hsp90 inhibited α-syn fibril formation and promoted α-syn oligomer accumulation whereas, in the presence of ATP and Hsp90, fibril formation was favored [253].

Overexpression of Hsp70 or Hsp40 reduced or prevented the formation of high molecular weight forms of α-syn in cellular PD models [254,255,256]. By inducing Hsp70, Geldanamycin effectively prevented α-syn aggregation in cell culture model of PD [257]. The chemical induction of Hsp70 by Carbenoxolone (CBX), a glycyrrhizic acid derivative, decreased α-syn aggregation and prevented α-syn-induced cytotoxicity in cell cultures [258]. Similarly, overexpression of CHIP inhibited α-syn inclusion formation and reduced α-syn protein levels, [168] increasing ubiquitination of α-syn both in vitro and in solution [259]. Hsp70 was also shown to inhibit α-syn toxicity in a *Drosophila melanogaster* and transgenic mouse model of PD [247,254]. Moreover, AAV-Hsp70 overexpression into dopaminergic neurons significantly protected the nigrostriatal pathway in MPTP mouse model [260]. Several studies suggested that Hsp70 interacted with prefibrillar α-syn species and interactions between the Hsp70 substrate binding domain and the α-syn core hydrophobic region was sufficient for assembly inhibition [261,262,263]. Cooperating with Hsp70 and Hsp40, Hsp104 reduced the formation of phosphorylated inclusions and prevented α-syn-induced neurodegeneration in a rat PD model [264]. More recently, the efficacy of Hsp110 in preventing or reducing α-syn aggregation was demonstrated in cell cultures and in double-transgenic α-syn/Hsp110 mice [265]. Development of activators promoting Hsp104 or Hsp110 functions could be interesting for the treatment of PD.

Several small molecules, by direct interaction with α-syn or indirect chaperone modulation, are able to reduce α-syn aggregation and neurodegeneration in vitro and in vivo using different animal PD models. As described, binding to lysine residues or C-terminal domain of α-syn, some small molecules can prevent and/or inhibit de novo fibrils. For some molecules, this beneficial effect is associated with neuroprotection and motor improvement in preclinical studies. Even if no clinical proof exists yet, these promising results encourage the development of small molecules for PD therapy. Hsp modulators could also be good candidates for future treatments of PD.

## 7. Increasing Clearance of α-syn

Two cellular pathways are involved in α-syn clearance trying to maintain its physiological protein levels: the ubiquitin-proteasome system (UPS) [266] and the autophagy-lysosomal pathway (ALP) [267,268,269]. UPS is involved in short-lived, damaged and misfolded protein degradation through a step of ubiquitination followed by a proteolysis, involving the action of multiple proteases. ALP is a complex process in charge of long-lived and aggregated protein degradation, as well as clearance of damaged organelles through multiple pathways including the non-selective macroautophagy (MA) and the selective chaperone-mediated autophagy (CMA). MA degrades cellular waste through the fusion of the autophagosomes carrying the material into the lysosomes containing the enzymatic material, whereas CMA degrades the proteins after the specific recognition of a pentapeptide KFERQ-like motif by the cytosolic chaperone heat-shock cognate 70kDa (Hsc70) and delivery of the targeted protein to the lysosomes through the Lysosomal-Associated Membrane Protein 2A (LAMP-2A). Alterations in both ALP pathways involved in α-syn clearance have been implicated in PD pathology by both genetic and post-mortem studies [40]. Therapeutic strategies aiming to increase the α-syn degradation through activation of these clearance pathways have thus been deeply explored in order to reestablish physiological levels of the protein and prevent from its accumulation and propagation in a PD context.

### 7.1. Activation of the UPS

The discovery of ubiquitin as a component of LB [266] as well as decreased proteasome activity measured in the SN of PD patients [270] already highlighted a detrimental alteration of the UPS contributing to PD neuropathology. In vitro, UPS inhibition was shown to increase the amount of ubiquitin-positive α-syn aggregates, confirming the significance of the proteasome system deficiency in α-syn accumulation [271,272]. It has also been shown that α-syn aggregates inhibited the catalytic 26S proteasome subunit through its direct interaction with the regulatory 19S proteasome subunit [273,274], pushing the cell into a vicious circle of detrimental factors leading to α-syn accumulation.

Various strategies have been investigated in order to increase UPS activity, but only few studies demonstrated the benefits directly achieved by an enhancement of the UPS-mediated α-syn degradation. A first study proposed to deliver additional free ubiquitin molecules to the cell in order to facilitate the labeling of the targeted protein and thus the overall proteasomal activity in a fly model of PD [275]. They showed that co-transfection of WT or K48-ubiquitin into neuronal cells of flies provided neuroprotection against α-syn-induced toxicity. In 2014, a plant extract from the *Rhodiola rosea L.*, called Salidroside, was shown to protect MPP^+^-treated PC12 cells and MPTP-treated mice by reducing expression of proteins involved in the UPS pathway regulation [276]. The group demonstrated that administration of Salidroside induced dopaminergic neuroprotection, associated with decreased levels of α-syn protein in MPTP-treated mice [276]. They confirmed the relevance of using Salidroside in a second study. They showed in vitro that transfection with WT or A30P α-syn vectors, after Salidroside treatment for 24h, increased clearance of α-syn by 30%, associated with a 60%–70% increase of the 20S proteasome activity [277]. Then, they administrated the molecule to 6-OHDA-treated SHSY5Y cells and showed increased cell viability and decreased levels of phosphorylated α-syn through UPS-dependent activation [277]. Similarly, a suitable screening assay found the PD163916 compound, which activated proteasomal activity through the inhibition of the p38aMAPK pathway [278]. They showed that PD163916 treatment in primary mouse neurons increased the proteasome activity and decreased α-syn levels, suggesting an increased clearance of α-syn through the UPS activation [278]. More recently, the T-006 compound properties, a derivative of tetramethylpyrazine, were explored for its potential therapeutic activities to enhance proteasomal α-syn clearance [279]. In an inducible PC12/A53T α-syn cell model, they demonstrated a dose-dependent decrease of soluble and insoluble α-syn levels upon T-006 treatment. T-006 was showed to activate the chymotrypsin-like UPS through LMP7 protein expression upregulation by activation of the PKA/Akt/mTOR/P70S6K pathway [279]. They confirmed the significance of the use of T-006 in vivo providing evidence that T-006 prevented from neurodegeneration and motor deficits and induced decreased α-syn levels in A53T α-syn transgenic mice [279]. Finally, as previously mentioned, neuroscientists used two nanobodies fused with a proteasome-targeting proline, aspartate or glutamate, serine, and threonine (PEST) motif targeting either the NAC domain or the C-terminal of the α-syn protein. They first demonstrated that treatment of WT-α-syn-overexpressed ST14A cells upon PEST-nanobodies treatment enhanced turnover of the α-syn protein through, at least in part, proteasomal activation [221]. They later showed that these two PEST-nanobodies reduced the levels of phosphorylated and aggregated α-syn in rats overexpressing the WT-α-syn in the SN [222]. PEST mediated clearance of α-syn also led to dopaminergic neuroprotection when the NAC-targeting nanobody was used in those animals [222].

Enhancing UPS seems efficient to prevent α-syn accumulation in PD neuropathology. Strategies aiming at targeting the UPS are still at their early stages and still need to be developed further. Nevertheless, it is important to note that distinct roles of UPS and ALP have been described in vivo. While UPS degrades α-syn in physiological conditions, ALP process could be in charge of degrading elevated intracellular α-syn levels in more advanced pathological conditions [280]. Enhancing α-syn degradation through ALP activation, by both CMA and MA, seemed thus compulsory in a PD context.

### 7.2. Activation of the CMA

α-syn is a substrate of the CMA as its protein sequence contains a KFERQ-like motif (VKKDQ) [281]. It has been demonstrated that CMA contributes to α-syn degradation in different cell types in vitro [268,269]. Through the same line of evidence, mutation in the CMA-recognition motif of α-syn sequence [268] or knocking-down the rate-limited LAMP-2A CMA receptor [282] led to accumulation of α-syn in vitro. CMA process has been suggested to be altered in PD brains as LAMP-2A and Hsc70 CMA protein levels are significantly reduced in the SN and the amygdala of PD patients [283]. Interestingly, this decrease was shown to be associated with α-syn accumulation at early stages of PD [284]. Reciprocally, CMA is also a target of α-syn toxicity as it has been demonstrated that A30P and A53T mutated α-syn [268], as well as dopamine-modified forms of α-syn [285,286], inhibited the CMA activity through their high affinity to the LAMP-2A receptor, associated with poor internalization and degradation into the lysosomal compartment. Some studies also pointed out a detrimental role of WT α-syn overexpression on CMA activity [286,287]. Therapeutic strategies aiming to increase α-syn clearance through CMA activation have thus been investigated to normalize the protein level and to prevent from its neuronal accumulation. In order to boost α-syn clearance through CMA, the rate-limited LAMP-2A receptor was overexpressed in the nigral dopaminergic neurons of WT-α-syn-overexpressed rats. This was a non-toxic, efficient strategy to enhance CMA-mediated pathological and aggregated α-syn clearance, associated with dopaminergic neuroprotective effects [288]. Another strategy to modulate LAMP-2A levels is based on the use of retinoic acid derivatives. In 2013, targeting the retinoic acid receptor by chemical retinoic derivatives led to a specific CMA activation through LAMP-2A transcriptional upregulation and could be relevant therapeutic molecules to enhance CMA-mediated α-syn clearance [289]. Finally, miR-based therapies were also considered to enhance CMA activity. Interestingly, post-mortem studies revealed that increased LAMP-2A and Hsc70-targeted miR levels were associated with decreased LAMP-2A and Hsc70 protein levels, which correlated with the severity of LB pathology in the SN and the amygdala of PD patients [290]. A more recent study notably demonstrated that miR-21 upregulation led to decreased LAMP-2A transcription and protein levels and contributed to increased levels of α-syn protein in MPP^+^-treated SHSY5Y cells and MPTP-treated mice [291]. This miR-21 expression was shown to be upregulated in the SN of PD patients [292]. Geniposide, an iridroid glucoside extracted from the fruit of *Gardenia jasminoides*, completely reversed the miR-21-induced effects, and decreased the α-syn levels through LAMP-2A upregulation in MPTP mice model [291].

### 7.3. Activation of MA and Lysosomal Function

Multiple evidence also suggested that the MA process is involved in the degradation of WT, mutated and aggregated forms of α-syn in different cellular models [267,268,269]. MA was shown to be recruited when the UPS activity was altered and intracellular levels of α-syn were elevated in α-syn-transgenic mice brains [280]. In PD conditions, alteration of MA has been observed with accumulation of the Microtubule-associated protein 1A/1B-light chain 3 (LC3) which colocalized with LB in the nigral dopaminergic neurons of PD patients [283,293]. This observation suggested a defective clearance of the autophagosomes by lysosomes. In the same line of evidence, decreased levels of lysosomal proteins such as the Lysosomal-Associated Protein 1 (LAMP1) and the ATPase Cation Transporting 13A2 (ATP13A2) have been observed in PD brains [294,295,296], confirming a deficit in lysosomal activity. Moreover, decreased enzymatic activity of the lysosomal protein β-glucocerebrosidase (GCase), a well-known genetic risk factor for PD, has been measured in the SN [297,298] of sporadic PD patients, and correlated with the accumulation of α-syn [299]. The role of MA deficits in α-syn accumulation have been directly demonstrated in rodents, notably with the conditional knock-out of the autophagic related gene 7 in dopaminergic neurons of mice which led to the accumulation of α-syn [300]. Inversely, α-syn has been shown to directly alter the MA function either by inhibiting autophagosome formation through the Rab1 pathway [286,301,302], by inhibiting HMGB1 and Beclin1 complex formation, which is essential for the autophagic activation [302], or by inducing lysosomal permeabilization [38,303]. Thus, multiple therapeutic strategies aimed to increase the MA activity in order to enhance α-syn clearance and to reverse the α-syn toxicity on the MA machinery. Although multiple molecules have been investigated as activators of the MA process, we will only present here the strategies which directly linked MA activation to α-syn clearance as therapeutic approaches.

As the upstream autophagic actor mTOR (mechanistic Target Of Rapamycin) negatively regulates MA, multiple approaches first aimed to activate MA through mTOR-dependent strategies. Rapamycin, an FDA-approved antibiotic, is an allosteric inhibitor of mTOR and was deeply studied as a MA enhancer in the context of PD. Rapamycin has been shown to increase α-syn clearance of WT and mutated forms in cellular models [267] and in α-syn transgenic mice [304] through activation of the MA. Rapamycin treatment also prevented neurodegeneration and motor deficits in human A53T α-syn transgenic mice through MA reestablishment, although they failed to prove any effect on human α-syn levels in their model [305]. However, another team succeeded in demonstrating beneficial effects in WT α-syn overexpressed rat model with decreased behavioral impairment and dopaminergic cell loss associated with reduced α-syn levels induced by an FDA-approved derivate of rapamycin, CCI-779 (temsirolimus) [306]. Nonetheless, the use of Rapamycin as a drug candidate for PD was limited due to its lack of specificity, its action on immunosuppression, and its poor solubility and stability in aqueous solutions [307]. mTOR-dependent MA induction can also be achieved through AMPK protein activation, an upstream effector of the mTOR pathway, that directly inhibits the mTOR protein. In this context, Resveratrol, a natural phytoestrogen found in grapes and red wine, was shown to directly increase MA activity through AMPK activation. Resveratrol treatment in MPTP-injected mice enhanced α-syn degradation through MA activation that finally attenuated neurodegeneration and motor deficits [308,309]. It was suggested that Resveratrol activated MA through the induction of the LC3 deacetylation by NAD^+^-dependent deacetylase SIRT1 activation in an AMPK-dependent manner [310]. mTOR signaling also regulates the action of the downstream transcription factor EB (TFEB) which emerged as the master gene regulator of autophagy machinery [311,312]. TFEB regulates the expression of autophagic genes through the Coordinated Lysosomal Expression and Regulation (CLEAR) signaling network, enhancing both lysosomal biogenesis and autophagy [313,314,315]. TFEB overexpression prevented motor deficits and induced dopaminergic neuroprotection associated with enhancement of α-syn clearance through MA activation in human WT and A53T α-syn overexpressing rats [306,316]. Similarly, TFEB overexpression in MPTP-intoxicated mice induced autophagosomal formation and lysosomal activity in addition to neurotrophic effects [317]. Chemical activation of TFEB using FDA-approved 2-hydroxypropyl-β-cyclodextrin (HPβCD) molecule also induced MA-dependent α-syn clearance in vitro and has been suggested to be a viable therapeutic strategy in a PD context [318,319]. More interestingly, pomegranate extract was shown to activate TFEB in vitro, increasing the nuclear localization of the transcription factor, and thus activated the MA process as well as mitophagy process [320].

mTOR-independent pathways have been also extensively explored to activate MA and induce α-syn clearance in the context of PD. Among the mTOR-independent autophagy enhancers, trehalose, a disaccharide found in invertebrates, was used to activate MA processes and showed efficiency to enhance clearance of WT and mutated forms of α-syn in cellular models [321,322,323]. Trehalose showed in vivo beneficial effects on the clearance of α-syn aggregates through MA activation associated with neuroprotective effects and prevention of motor deficits in AAV-human-A53T α-syn rats [237]. Similarly, a short intake of trehalose for one week was sufficient to induce autophagy in mice brains and to decrease insoluble α-syn levels in a transgenic mouse model overexpressing human A53T α-syn [324]. They showed that MA-activation seemed to occur through the phosphorylation of the autophagic Beclin-1 modulator at serine 15 [324]. The mechanisms behind trehalose-mediated MA activation were further investigated and demonstrated that trehalose activated MA through the inhibition of the glucose transporter SLC2A, finally leading to the activation of the AMPK protein [325]. More recently, a pharmacokinetic study showed that a daily oral administration of trehalose was more efficient than administration of the same dose by drinking water in A53T α-syn rat model of PD, and prevented from α-syn-mediated neurodegeneration and motor deficits [326]. They also showed that trehalose oral administration was efficient to prevent from α-syn-induced striatal dopaminergic deficits in a macaque model overexpressing human A53T α-syn, although they failed to show beneficial effects on α-syn levels [326]. Altogether, trehalose has been identified as an interesting drug to target α-syn, but the correct dose and administration remains to be identified. Nilotinib, an FDA-approved second-generation cAbl inhibitor, was also investigated as a potential MA enhancer through a mTOR-independent pathway. Nilotinib administration in a transgenic A53T α-syn mouse induced clearance of accumulated α-syn leading to dopaminergic neuroprotection and decreased motor deficits in this model [327]. The involvement of the Nilotinib-induced MA activation in α-syn clearance was confirmed by demonstrating that in vitro inhibition of cAbl protein by Nilotinib prevented α-syn phosphorylation at the Y39 residue [328]. Clinical studies investigating the therapeutic interest of Nilotinib have been recently conducted on PD patients. The first clinical study demonstrated that administration of Nilotinib at 150 mg and 300 mg doses appeared to be safe and well-tolerated by twelve advanced PD patients with the limited observation of a reduction in blood and CSF α-syn levels [329]. A more recent large-scale clinical study enrolling seventy-five participants confirmed these results with an optimal daily dose of Nilotinib at 200 mg for one year that has been shown to be efficient for the reestablishment of dopaminergic metabolism associated with decreased levels of oligomeric α-syn in the CSF [330]. Another mTOR-independent enhancer has been developed aiming to directly target the autophagic activator Beclin-1 protein through genetic or pharmacological approaches. Following Beclin-1 lentiviral injection in the temporal cortex and hippocampus of α-syn transgenic mice, a decrease in α-syn levels was observed through autophagy activation [331]. This activation was mediated through an enhancement of HMGB1-Beclin1 complex formation, an essential step to induce MA, which in turn increased α-syn clearance [332]. Moreover, Isorhynchophylline, an alkaloid derived from a Chinese herbal plant, was efficient to induce α-syn clearance through a Beclin-1-mediated MA activation in different cellular models, thus opening the way for alkaloid screening as Beclin-1 dependent autophagic inducers [333]. Interestingly, chronic caffeine treatment has also been studied as a mTOR-independent autophagy enhancer. Given that human coffee consumption has been associated with a reduced risk of developing PD [334], caffeine could be inducing MA through activation of the adenosine receptor [335]. Chronic caffeine drinking during four months in human A53T α-syn fibrils-injected mice reversed α-syn-induced MA defects and decreased levels of phosphorylated α-syn in the injected site [335].

Finally, an increase in activity of the lysosomal enzymes responsible for α-syn degradation could be an appealing approach to induce MA-associated clearance of α-syn. *GBA1* encodes the lysosomal enzyme GCase. Related to PD, *GBA1* mutations are the most common genetic risk factor for developing PD and GCase activity has been demonstrated to be defective in fibroblasts derived from PD patients harboring the *GBA1* mutations [336,337] or in a mouse model of Gaucher’s disease that presented α-syn accumulation [338]. Interestingly, decreased GCase activity has been observed in the SN of sporadic PD patients [339] and has been shown to correlate with increased α-syn levels at the early stage of the disease [340]. Moreover, reduced GCase activity was shown to influence α-syn aggregation through stabilization of soluble and toxic oligomeric intermediates [341,342]. This phenomenon seemed to be dependent on neuron type, on the level of extent pathology and on the stages of the pathology [343]. In return, pathological α-syn was shown to decrease lysosomal hydrolase activity, including GBA1, possibly due to a disruption of proper enzyme targeting from endoplasmic reticulum to lysosomal compartment [341,344]. All these data pointed out GBA1 enzyme as a high-relevant therapeutic target to increase α-syn clearance and decrease α-syn pathological aggregation [345]. Ambroxol hydrochloride, a safe FDA-approved molecule, was proven to enhance GCase activity and to increase α-syn clearance [336,337]. WT and α-syn transgenic mice treated with Ambroxol presented increased brain activity of GCase, associated with decreased levels of total and phosphorylated α-syn protein in different brain regions of the transgenic model [346]. Based on the results obtained after Ambroxol treatment on Gaucher’s disease patients [347] and on PD rodent models, a pilot study in humans has been launched recently in order to determine the effective dose and to prove the efficacy of such a strategy on seventy-five PD patients [348]. In parallel, another recent clinical trial enrolling seventeen PD patients and showed that Ambroxol crossed the BBB and increased the GCase activity in patients both with and without *GBA1* mutation [349]. The iminosugar isofagomine (IGF) was shown to increase GCase activity in vitro [350] and in mice [351] in a tissue-specific manner [352], making this molecule interesting for approaches targeting lysosomal activity of GCase. Histone deacetylase inhibitors such as IGF have been implicated in GCase activation through hyperacetylation of the chaperone Hsp90 that enabled the appropriate folding of the GCase protein and the elimination of the mutated forms of the protein [353]. A study succeeded in confirming that oral administration of IGF in WT α-syn overexpressed transgenic mice increased α-syn clearance through lysosomal activation and improved motor performances, supporting the interest of using pharmacological chaperones in a PD context [354]. Using high-throughput screening, two GBA1 chaperone compounds, NCGC607 and NCGC758, have been tested in iPSC-derived dopaminergic neurons from PD patients and induced improved GCase activity as well as decreased levels of α-syn and GCase substrates [355,356]. In the same line of evidence, using a Gaucher’s disease mice model, administration of another GCase modulator S-181 that stabilizes wild-type GCase protein was sufficient to decrease lipid substrates and α-syn in mice brains [357]. Targeting another lysosomal hydrolase, direct overexpression of Cathepsin D gene into mammalian cells and human α-syn-transgenic *C. elegans* induced neuroprotective effects against α-syn aggregation and toxicity [358].

Finally, combined approaches have been developed to enhance MA through both mTOR-dependent and mTOR-independent pathway in order to improve α-syn clearance. A synergistic effect using Rapamycin and Trehalose treatment on dopaminergic neuroprotection was shown in the MPTP mouse model of PD through active enhancement of MA process [359].

In conclusion, enhancing the degradation of α-syn species mediated by the UPS, CMA, MA or lysosomal enzymes using multiple approaches and targets are appealing therapeutic strategies in a PD context. Given that proteasomal and autophagic activity are decreased in an age-dependent manner and in pathological conditions, collective efforts should be pursued to find efficient drug candidates to enhance these pathways independently or synergistically. Likewise, future investigations must be focused on combined therapies in order to increase the promising of such therapies in a PD context.

## 8. Anti-Oxidative Strategies

Natural and endogenous antioxidants have been evaluated in vitro and in vivo as therapeutic agents for preventing and delaying the development of PD. Indeed, they have shown indirect protective effects against oxidative-induced neuronal death, and/or direct interaction with different forms of α-syn decreasing its toxic aggregation.

### 8.1. Polyphenols

Polyphenols, a group of chemical substances present in plants, fruits, vegetables or tea, have been studied for their antioxidant property associated to the capacity to prevent and to reduce the protein aggregation. Within anti-oxidant polyphenols, there are two families that have been shown to impact α-syn aggregation: phenolic acid derivatives and flavonoids.

Amongst phenolic acid derivatives, curcumin, chemically known as diferuloylmethane, was shown to have beneficial effects in neurodegenerative diseases, including PD, through its anti-oxidant, anti-inflammatory and anti-protein aggregation properties [360]. It was found that curcumin could inhibit α-syn fibril formation and destabilize preformed fibrils [361,362]. The anti-aggregative effect of curcumin occurred through interaction of the molecule to the hydrophobic NAC domain of α-syn via both hydrophobic and hydrogen bonding [363,364]. Curcumin interacted with both α-syn monomers and oligomers via its phenolic groups [365], but was found to preferentially bind oligomeric intermediates [360]. Conformational changes of α-syn affected the extent of binding of curcumin to α-syn and its potential in inhibiting oligomers or fibrils [360,364,366]. In addition, curcumin significantly decreased the cytotoxicity of preformed α-syn oligomeric species in SH-SY5Y cell line [367] and in PC12 cells [368]. Moderate doses of curcumin increased the level of phosphorylated forms of α-syn at cortical presynaptic terminals and improved motor behavior performance in mice overexpressing human GFP-tagged WT α-syn [369]. Its anti-aggregative effect on α-syn was also shown in dopaminergic neurons of a rat model of lipopolysaccharide-induced PD [370]. Whereas some studies suggested the interest of curcumin to treat PD, its potential efficacy is limited owing to its poor stability and bioavailability. For this reason, many nanoformulations or stable curcumin analogs were evaluated against α-syn aggregation, fibrillation, and toxicity. Curcumin pyrazole derivatives reduced the toxicity of both WT and mutant A53T α-syn by preventing fibrillation and disrupting preformed fibrils [371]. In the same way, the biphenyl analogs of dehydrozingerone and O-methyl-dehydrozingerone reduced α-syn aggregation [372]. Additionally, a curcumin-glucoside derivative prevented oligomers and inhibited fibril formation in a dose-dependent manner [373]. Amine-functionalized mesoporous silica nanoparticles of curcumin showed interaction with α-syn species and prevented fibrillation [374]. A nanoformulation containing curcumin and piperine with glyceryl monooleate nanoparticles has been shown to prevent α-syn oligomerization and fibrillation [375]. Another nanoformulation prepared with lactoferrin by sol-oil chemistry reduced α-syn expression in dopaminergic SK-N-SH cells treated with rotenone [376]. Combining curcumin with β-cyclodextrin showed a synergistic inhibition of α-syn aggregation and degraded the preformed aggregates into monomers at very low concentrations [365,377]. Finally, an analog, the liposomal nanohybrid of curcumin with polysorbate 80-modified cerasome ameliorated motor deficits and improved dopamine expression by promoting α-syn clearance in the MPTP mouse model [378]. Gallic acid (GA), another type of phenolic acid chemically known as 3,4,5-trihydroxybenzoic acid, is found in its free form or as part of the hydrolysable tannins in many plants [379]. GA and its structurally similar benzoic acid derivatives also showed anti-aggregating effects [380]. In PD models, GA was shown to inhibit α-syn fibrillation and toxicity and to disaggregate fibrils of α-syn [380,381]. GA also bound to soluble and non-toxic oligomers and stabilized their structure. It has been shown that the number of hydroxyl groups and their presence on the phenyl ring in these structural derivatives of GA were responsible for binding and inhibiting α-syn fibrillation [381]. Finally, tyrosol, a simple phenol present in Extra Virgin Olive Oil, was also shown to be effective by reducing α-syn inclusions in a *C. elegans* model of PD. Moreover, in this in vivo model, Tyrosol had a protective effect on dopaminergic neurons, reduced ROS level and promoted the expression of specific chaperones and antioxidant enzymes [382].

Within polyphenols, flavonoid compounds have also been shown to impact α-syn aggregation. The flavonoid Baicalein, isolated from the roots of *Scutellaria baicalensis Georgi*, a Chinese herbal medicine [383] is a well-known potent antioxidant. Several studies demonstrated the efficiency of Baicalein to prevent α-syn oligomerization and fibrillation [384,385]. Baicalein had a strong effect in inhibiting α-syn oligomer formation and in disaggregating pre-formed oligomers at low concentrations [386,387]. Baicalein inhibited the formation of high molecular weight α-syn oligomers and protected against neurotoxicity in Hela and SH-SY5Y cell lines [388]. By tightly binding to α-syn, Baicalein stabilized its natively unfolded conformation [384] whereas another study specified that Baicalein-stabilized oligomers were β-sheet-enriched [389]. In another study, Baicalein reduced α-syn in the media of dopaminergic cell lines overexpressing A53T α-syn [390]. The Baicalein derivative N′-benzylidene-benzohydrazide also inhibited oligomer formation [391]. Baicalein in combination with β-cyclodextrin synergistically inhibited α-syn aggregation and disaggregated preformed fibrils even at very low concentrations [365]. Two studies also reported the in vivo protective effects of Baicalein on decreasing α-syn aggregation [392,393]. Baicalein prevented the transition from α-syn monomers to oligomers, associated with behavioral improvement and neuroprotection in the striatum of rotenone-induced PD in rats [392]. Furthermore, Baicalein attenuated MPTP-induced α-syn aggregate formation in the injected SN of mice [393]. This strong inhibition was mostly due to the formation of Schiff base [384] and the presence of vicinal dihydroxyl group on the phenyl ring of Baicalein that could be responsible for its anti-aggregative action [388]. Integrating evidence from in vitro and in vivo studies, Baicalein appears to be a potential drug candidate to inhibit α-syn aggregation, fibrillation, and propagation in neurons. Interestingly, another polyphenol, Cuminaldehyde, isolated from Iranian cumin, was found to be superior to Baicalein. It inhibited α-syn fibrillation [394] and blocked protein assembly into β-structural fibrils by the interaction with its amine and aldehyde groups with α-syn [395]. In the same line of evidence, one of the most popular catechins, (−)-Epigallocatechin 3-gallate (EGCG), a flavanol compound predominantly present in green tea, inhibited α-syn aggregation and fibrillation in a concentration-dependent manner [396]. In primary cortical neuron cultures challenged with oxidative injury, EGCG inhibited fibrillation of α-syn and apoptosis [397]. EGCG also inhibited α-syn aggregation using PD post-mortem tissue [398]. The molecular mechanisms by which EGCG blocks amyloid aggregation are not yet completely understood. However, EGCG bound the natively unfolded polypeptides directly and prevented their conversion into toxic intermediates [399]. Moreover, it can induce a conformational change by binding directly with β-sheet-rich aggregates without disassembling them into monomers or small diffusible oligomers [400,401,402]. It was also notably suggested that EGCG had the potential to bind to the oligomeric state of α-syn, destabilizing it and blocking the membrane affinity of α-syn [403]. EGCG was later showed to exhibit its protective effect by facilitating the conversion of active oligomers, which could exert membrane disruption and cellular toxicity, into amyloid fibrils [404]. The effects of EGCG have also been related to metal homeostasis, which will be discussed later in this review [405]. Finally, some studies suggested the hydrophobic binding and Schiff base formation with Lysine residues as important features of its mechanism of action [400,406]. Although EGCG has been shown to be neuroprotective in MPTP-induced animal models of PD, there is no proof about its direct protective effect against α-syn aggregation toxicity in an in vivo model [398]. However, when EGCG was conjugated with nanoparticles, it allowed a neuroprotective effect and a considerable inhibition of α-syn aggregation in a mouse model of PD [407]. Finally, Theaflavins, another polyphenol present in fermented black tea, had effects comparable to EGCG but it appeared less vulnerable to oxidation by air and exhibited better activity in oxidizing environments compared to EGCG [408].

### 8.2. Other Antioxidant Strategies

Besides the use of polyphenols, other antioxidant strategies have been investigated for their potential to reduce α-syn aggregation. Among them, coenzyme Q10 (coQ10) is an important anti-oxidant in both mitochondria and in lipid membranes [409,410]. coQ10 administration protected against MPTP toxicity in mice [411,412] and reduced α-syn aggregation [412,413]. A first positive human trial for coQ10 was reported [414] but, in the end, the investigational drug associated with Vitamin E was unlikely to demonstrate efficacy over placebo for this indication in a phase III (NCT00740714). The mechanisms by which coQ10 protects dopaminergic neurons against degeneration are not well understood. However, coQ10 as an antioxidant attenuated changes in H_2_O_2_ measured in PD cybrid cells by increasing complex I activity and inhibiting α-syn oligomerization [415]. Another molecule, ginseng, also known as red ginseng (*Panax ginseng, Araliaceae*), is a well-known medicinal plant and popular source of saponins. Several studies identified the biological active components of ginseng, the ginsenosides, that were shown to play many protective roles [416]. The most frequently used and studied ginsenosides, Rg1, Rg3, and Rb1, were investigated for their effect on α-syn aggregation in vitro [417,418]. Ginseng extract reduced dopaminergic cell loss, microgliosis, α-syn aggregate buildup, and improved locomotor activity in a PD rat model [419]. In another in vivo study, Rg1 attenuated neurodegeneration in the MPTP mouse model of PD and reduced oligomeric and phosphorylated α-syn in the SN [418]. An alternate anti-oxidant molecule, Apelin, was proved to be a neuroprotective peptide, which was first extracted and purified from bovine stomach [420]. In vitro, Apelin-36 was neuroprotective in MPP^+^-treated SH-SY5Y cells [421]. Furthermore, in MPTP-induced PD mice, they demonstrated that the neuroprotection induced by Apelin-36 could be explained by its effect on the reduction of oxidative stress and nitrated α-syn expression. Apelin-36 also promoted autophagy and inhibited ASK1/JNK/caspase-3 apoptotic pathway [422]. Finally, the activation of the transcription factor Nrf2/antioxidant response element (ARE) pathway has shown to protect against neurodegeneration by decreasing both oxidative stress and protein aggregation [423]. Nrf2 reduced α-syn toxicity by a time-dependent, cell-autonomous mechanism. Nrf2 accelerated the clearance of α-syn, shortening its half-life and leading to lower overall levels of α-syn [424]. Scopoletin, an active principle obtained from *Morinda citrifolia*, also presented antioxidant property [425] by quenching free radicals, reversing apoptosis in rotenone-treated SH-SY5Y cells and prevented α-syn aggregation in rotenone-treated rats through activation of DJ-1/Nrf2/ARE pathway [426]. Additionally, a synthetic morpholine-containing chalcone, KMS99220, reduced α-syn aggregation in GFP A53T α-syn-overexpressing cells. In MPTP-treated mice, oral administration of KMS99220 prevented degeneration of the nigral dopaminergic neurons, induced the Nrf2 target genes, and prevented the associated motor deficits [427].

To conclude, antioxidants have a strong power to inhibit α-syn aggregation by direct interaction with the protein for polyphenols, as well as by its other beneficial properties, such as antioxidative, anti-inflammatory and pro-autophagic activities. These specific properties can have indirect effects on the reduction in protein aggregation, activating antioxidative enzymes or pathways and decreasing oxidative stress. Polyphenols interact directly with the monomeric or oligomeric forms of α-syn mainly through the hydroxyl groups by forming hydrogen bonds with residues of α-syn [385]. By such hydrophobic interactions, polyphenols stabilized the natively unfolded conformation of α-syn, and disaggregated preformed fibrils into monomers or soluble and non-toxic oligomers [384,400]. Even if no clinical studies confirm their beneficial effects, antioxidants, by their anti-aggregative and neuroprotective efficacy in vitro and in vivo, seem to be interesting candidates for PD therapy.

## 9. Metal Dyshomeostasis

Divalent metals such as iron, copper, and zinc have been shown to interact with α-syn via multiple metal binding sites and affect its stability and aggregation [41,43,428,429,430]. To target therapeutically the interactions between metals and α-syn, different strategies have been used including chelation, metalloproteases and several metal-interfering molecules.

### 9.1. Targeting Iron Homeostasis

Iron has been primarily studied for its effects on PD pathology progression, particularly in its interaction with α-syn and induction of dopaminergic cell loss [352,431,432]. α-syn phosphorylation and expression were increased both in SH-SY5Y cells and in rats when exposed to higher amounts of iron, suggesting the key role of iron in regulating α-syn expression and S129 phosphorylation [433].

Chelation-based therapy, a strategy based on the depletion and/or sequestration of metals using chelators, has mostly been studied in the case of iron and has showed some promising findings. Deferiprone is an iron chelator that has the capacity of crossing the BBB, making it of great interest in the study of chelation for PD. Deferiprone decreased neuronal cell loss in human dopaminergic neurons in vitro and in MPTP mice by decreasing oxidative stress without interfering with iron-dependent mechanisms [434]. Additionally, deferiprone was able to rescue behavioral deficits and trended to decrease α-syn accumulation in iron-fed A53T α-syn-overexpressing mice [435]. Phase 1 clinical trials of this drug also showed that 12 months of deferiprone administration decreased disease progression in PD patients compared to the placebo group [434]. Deferiprone was also tested in a phase 2 clinical trial using 22 PD patients where it showed to decrease iron and have little side effects on the patients [436]. Similarly, intranasal administration of iron chelator deferoxamine in α-syn overexpressing rats improved motor defects, decreased both the number and size of α-syn aggregates, and reduced inflammation. However, this molecule did not rescue the loss of dopaminergic neurons in this animal model [437]. 8-Hydroxyquinoline (8-HQ)-derived chelators have also shown great potential for treating neurodegeneration and α-syn aggregation. The beneficial effects of clioquinol, an 8-HQ derivative, were first demonstrated in MPTP-intoxicated mice [438]. Treatment with clioquinol led to a decrease in iron and induced alleviation in oxidative stress, increase of striatal dopamine loss and neuroprotection. Furthermore, clioquinol improved cognition, motor functions and dopaminergic cell loss in transgenic A53T α-syn-overexpressing mice [439]. A concomitant treatment of clioquinol and L-DOPA in A53T α-syn-transgenic mice not only prevented neurodegeneration but also improve motor symptoms [440]. Q1 and Q4 are other 8-HQ derivatives that target specifically mitochondrial and cytosolic iron pools respectively. Oral administration of these iron chelators in MPTP-treated mice were able to protect from mitochondrial and cytosolic iron increase, against oxidative stress, and dopaminergic cell loss [441]. Other 8-HQ derivatives such as VK-28, M30, and HLA20 were also tested in different models to determine their therapeutic potential and were capable of reducing oxidative stress, iron accumulation, and α-syn accumulation [442,443]. D-607 is another iron-chelating molecule that is capable of activating D2/D3 dopamine receptors [444]. In vitro, D-607 was able to increase cell viability in PC-12 exposed to 6-OHDA. In fly expressing WT or mutant α-syn, D-607 protected from α-syn toxicity by reducing multimeric species with a slight increase in monomeric α-syn. Neuroprotective effects of D-607 were also assessed in MPTP-injected mice and treatment with this iron-chelator inhibited dopaminergic cell loss. These in vitro and in vivo data demonstrated the neuroprotective effects of D-607 by reducing α-syn toxicity and dopaminergic loss [445]. Recently, PBT434, another chelator binding iron, has been tested in neuroblastoma cells and was able to inhibit iron-mediated oxidative stress as well as aggregation of α-syn. In the same study, they tested the effects of PBT434 in three in vivo PD models: 6-OHDA, MPTP and human A53T α-syn transgenic mice. In these mouse models of PD, PBT434 was able to rescue dopaminergic cell loss in the SN, which translated by an improvement in motor behavior. Most importantly, PBT434 decreased the levels of α-syn in either MPTP and human A53T α-syn mice [446]. This molecule shows promise as it is a low-binding chelator and could thus have less side-effects compared to other chelators.

Another strategy that has been tested for iron-targeting is the use of Rosmarinic acid (RA), an ester of caffeic acid. RA has been shown to have multiple biological roles including anti-inflammatory, antioxidative and antiviral activities. In 2010, this molecule demonstrated its protective effects in vitro as it was able to antagonize the neurotoxic effects of MPP^+^ exposure in dopaminergic cells [447]. Recently, these neuroprotective properties were also observed in a MPP^+^/MPTP context tied to iron-induced α-syn aggregation. In cells, RA protected cells against iron-induced toxicity and decreased α-syn aggregation. In mice, treatment with RA protected against the decrease in tyrosine hydroxylase and superoxide dismutase expression, inhibited the increase in mesencephalic iron content, and inhibited the increase in α-syn mRNA induced by iron [448].

Finally, another approach has been to target α-syn expression at the RNA level. The 5′-untranslated region (5′-UTR) of *SNCA* is well structured and contains an iron-response element (IRE) region that is capable of regulating its translation. At low iron concentrations, the IRE is bound by the iron response protein (IRP) whereas at high iron concentrations, IRP binds to iron and frees the IRE which induces translation of *SNCA*. A study attempted to create a small molecule to target the *SNCA* IRE to regulate its translation in PD models. They designed a compound, synucleozid, that was capable of targeting α-syn mRNA 5′UTR in a neuronal cell line, inducing a decrease in protein expression and a protective effect in cells [449]. This strategy of targeting the SNCA IRE by this small molecule could prove to be efficient in reducing α-syn and thus its toxicity and aggregation, but in vivo studies remain to be seen.

### 9.2. Targeting Zinc and Copper Homeostasis

Besides iron, zinc and copper are essential healthy cellular elements and have been shown to also interact with α-syn. Copper has been shown to bind α-syn with the most affinity, despite mutations in certain residues, and can accelerate α-syn aggregation [42,450,451]. In addition, zinc dyshomeostasis due to the loss of its transporter ATP13A2 was showed to induce an accumulation of α-syn and reduce its exosomal transport [452,453]. In another study, overexposure to zinc in WT rats prompted a PD-like pathology. This zinc-induced Parkinsonism showed a loss of dopaminergic cells, motor impairment, aggregation of α-syn and impairment of UPS-mediated degradation. Altogether, zinc exposure induced consequences that were similar to those seen in sporadic PD and were reversible by treatment with L-DOPA [454].

As previously stated, the polyphenol EGCG has been shown to bind to α-syn and inhibit its fibrillation in PC12 cells [455]. Following these results, another study aimed at determining if EGCG had an effect on copper-mediated α-syn aggregation. α-syn overexpressing PC12 cells treated with both copper and EGCG exhibited a decrease in cell loss, in ROS production, and in α-syn accumulation [456]. EGCG was determined to be able to bind copper, thus inhibiting the copper binding on α-syn. This was also studied in the case of iron in PC12 cells, where EGCG had similar chelating effects on iron, thus inhibiting metal binding on α-syn [457]. Combined, these studies show the potential beneficial effects of EGCG on metal-induced α-syn accumulation and aggregation.

Another potential therapy that could be used to target metals in PD is by regulating the expression or function of metal-binding proteins. Metallothioneins (MT) are small copper/zinc binding proteins that are instrumental in homeostasis of these two metals. Dexamethasone, a glucocorticoid which acts on the MT gene promoter and can thus activate MT mRNA expression, rescued mice from dopaminergic cell loss and inflammation after MPTP exposure [458]. Dexamethasone was also used in SH-SY5Y cells to increase MT expression and found that this suppressed copper-induced α-syn aggregate formation in vitro [459].

To conclude, modifying metal distribution in the brain via chelators or other molecules has proven to inhibit neurodegeneration, oxidative stress, and α-syn accumulation. Targeting metals in PD remains a relatively novel approach, with only one iron-based compound that has crossed into clinical trials. Nonetheless, promising studies are underway, with the goal of reducing α-syn aggregation without affecting the other proteins to which they bind as well. Another caveat of targeting metals is their importance within cellular mechanisms in the brain but also throughout our whole organism. Thus, it is important to keep in mind that metal therapies should beware of the high risks of side-effects.

## 10. Challenges and Open Questions

The present review describes a myriad of strategies. We have been careful in, so far, not rating them according to a degree of confidence, giving the feeling that they hold a similar translational potential, which they obviously do not. Amazingly, some have reached clinical development with, what we consider, a very limited package of evidence. Although this is in agreement with the concept of clinical equipoise that relieves the need to achieve the impossible ideal of preclinical certainty that a therapeutic strategy will work in patients, the selection of interesting candidates should be based on the soundest clinically driven preclinical validation [460].

How can one establish a confidence rating system and what variables shall be integrated into such a system? We will not solve here an issue that is the cornerstone of the industrial therapeutic development. However, a number of steps should be, in our opinion, fulfilled. The first criterion shall be the demonstration of the presence of the affected mechanisms in human samples, whether they are post-mortem tissues and/or biological fluids. The chosen cellular and, later, animal models shall exhibit comparable changes reminiscent of what happens in the human pathology. It is striking to observe that these basic considerations are not even fulfilled in most studies. In addition, the vast majority of the preclinical studies involve only one PD animal model, raising immediately the concern of the actual recapitulation of the pathology/pathogenesis by a single animal model. Nowadays, our field has the unique opportunity to use different animal species and different triggers for inducing different aspects of α-syn pathology, starting, from a historical point of view, from neurotoxin, transgenic animals, viral-based models, and finally the use of different inocula containing α-syn aggregates (recombinant or human brain-derived). The adoption of several and intrinsically different animal models should become standard for the community to cross-validate a positive result, in a single laboratory as well as between independent laboratories.

Similarly, the issue of the experimental design that leads to the demonstration of efficacy of given therapeutic strategies in these models is astonishingly not taken into account in most (if not all) translational studies. While PD is a progressive neurodegenerative disorder, a large majority of α-syn-related therapeutic candidates have been tested using a prophylactic exposure or a concomitant administration. While PD patients are likely to receive a neuroprotective agent following diagnosis—that is, when the extent of dopamine neuron degeneration is already approximately 50% [461,462]—therapeutic candidates are tested in association with, or even weeks before, α-syn-related triggers of pathology. What is the relevance of such an administration protocol with regard to the natural progression of PD? It is not at all surprising that, despite the strength of the available PD models, they have not identified a neuroprotective agent that has been shown to be efficacious in PD patients, even in non-human primate models.

The definition of the actual therapeutics objectives are also critical. With the example of Alzheimer’s disease, one should be very cautious in using the terminology “neuroprotection”, “disease-modifying”, etc. What does one try to achieve with one given strategy? Truly protecting the neurons from degenerating? Slowing down the prion-like spread of the α-syn aggregates? Decreasing the monomeric α-syn load? Dampening the phospho-α-syn pathology? Most papers are unclear about the true objective and importantly about how the communicated result can translate into an exploitable clinical trial endpoint.

Another level of complexity refers to the strategies for brain drug delivery employed, i.e., gene therapy or pharmacological drugs in disease models of pathogenicity. For these, critical points have to be fulfilled, such as no toxic or adverse effects, a suitable and efficient biodistribution, and evidence of target engagement in vivo. The booming field of synucleinopathies, if it does not build upon past failures, is likely to meet the same issues as other neurodegenerative conditions. It is thus time for building large consortiums of academic labs and to establish, in coordination with clinicians, the minimal package of data that would convince us to move an appealing preclinical finding into real life, i.e., in the clinic.

## 11. Conclusions

Given the central role of α-syn in PD pathology and progression, α-syn met the criteria to be a tantalizing and evident therapeutic target for PD. In this review, we discussed the potential strategies that are currently being investigated to reduce or block α-syn accumulation and propagation. Whether the strategies target α-syn directly (via gene silencing, immunotherapy or small molecules) or indirectly (via its clearance), they all aimed at restoring cellular homeostasis by bringing α-syn back to its physiological levels, non-aggregative and toxic state or by inhibiting the propagation of pathological forms of α-syn. Despite the various strategies described here, individual challenges remain for each approach. Many research efforts have been made in the various technologies/methodologies aiming to target α-syn, giving rise to multiple clinical trials currently underway (Table 1). Within these many strategies, some currently seem more promising than others, or have at least progressed more rapidly in clinical trials. These include predominantly immunization, anti-aggregative molecules and an increase in α-syn clearance, compared to the less developed PTM targeting and anti-oxidant strategies. Ultimately, we could envision that one possible solution could be combining different strategies, both direct and indirect, to target α-syn accumulation at different steps and both intracellularly and extracellularly. Nevertheless, scientists face multiple obstacles with clinical trials including BBB crossing, solubility, biodistribution, administration and toxicity. Finally, another challenge for all strategies is the lack of knowledge of the physiological roles of α-syn, as well as the absence of valid biomarkers for α-syn species accumulation. These biomarkers could have the potential to not only diagnose pre-symptomatic patients, but also to stratify patients for future trials according to their PD type (i.e., familial or idiopathic). Such could be the case for isoforms of apoE, which have been differentially implicated during PD progression [463,464]. Despite these challenges, it seems that it is only a matter of time before α-syn-based therapeutic strategies are successful in slowing PD progression.

## Figures and Tables

**Table 1:** Drug-based clinical trials targeting α-synuclein accumulation directly or indirectly.

	Molecule	Mechanism	Clinical Trial Phase	Year	Location of Trial	Reference
Immunotherapy	**PRX002**	Monoclonal antibody targeting C-terminal sequence of α-syn (amino acids 118–126)	1 (healthy volunteers)	2016	United States	NCT02095171 [190]
			1 (healthy volunteers and PD patients)	2017	United States	NCT02157714 [191]
			2 (PD patients)	Active	United States	NCT03100149
	**MEDI1341**	Monoclonal antibody targeting C-terminal sequence of α-syn	1 (healthy volunteers)	Recruiting	United States, United Kingdom	NCT03272165
	**BIIB054**	Monoclonal antibody targeting N-terminal aggregated forms of α-syn	1 (healthy volunteers and PD patients)	2018	United States	NCT02459886 [198]
			2 (PD patients)	Recruiting	United States, Japan	NCT03318523 NCT03716570
	**BAN0805**	Antibody targeting protofibrils of α-syn	1 (healthy volunteers)	Recruiting	United States	NCT04127695
	**PD01/PD03 Affitopes**	Vaccines targeting the C-terminal sequence of α-syn via small peptides	1 and 2 (healthy volunteers and PD patients)	2018	Austria	NCT01568099 NCT02216188
Clearance	**Nilotinib**	Tyrosine kinase Abelson (cAbl) inhibitor	1 (PD, PDD and DLB patients)	2016	United States	NCT02281474 [329]
			2 (PD patients)	2019	United States	NCT02954978 [330]
	**Ambroxol**	Pharmacological chaperone of β-glucocerebrosidase	2 (PDD patients)	Recruiting	Canada	NCT02914366 [348]
			2 (PD patients)	2020	United Kingdom	NCT02941822 [349]
Small molecules	**ANLE138B**	Small molecule targeting oligomeric forms of α-syn	1 (healthy volunteers)	Recruiting	United Kingdom	NCT04208152
	**NPT200-11**	Small molecule targeting the C-terminal region of α-syn	1 (healthy volunteers)	2016	United States	NCT02606682
Anti-oxidants	**CoQ10 + Vitamin E**	Antioxidant activity	3 (early PD patients)	2013	United States	NCT00740714
Metals	**Deferiprone**	Chelation of iron	1 (PD patients)	2012	France	NCT00943748 [434]
			2 (PD patients)	2019, Recruiting	France, Canada, Austria	NCT02728843 NCT02655315

Abbreviations: α-syn, α-synuclein; PD, Parkinson’s Disease; DLB, Dementia with Lewy Bodies; PDD, Parkinson’s Disease Dementia.

## References

[1] Pringsheim T., Jette N., Frolkis A., Steeves T.D. (2014). The prevalence of Parkinson’s disease: A systematic review and meta-analysis. Mov. Disord..

[2] Jankovic J. (2008). Parkinson’s disease: Clinical features and diagnosis. J. Neurol. Neurosurg. Psychiatry.

[3] Poewe W., Seppi K., Tanner C.M., Halliday G.M., Brundin P., Volkmann J., Schrag A.E., Lang A.E. (2017). Parkinson disease. Nat. Rev. Dis. Primers..

[4] Spillantini M.G., Schmidt M.L., Lee V.M.Y., Trojanowski J.Q., Jakes R., Goedert M. (1997). α-Synuclein in Lewy bodies. Nature.

[5] Shahmoradian S.H., Lewis A.J., Genoud C., Hench J., Moors T.E., Navarro P.P., Castano-Diez D., Schweighauser G., Graff-Meyer A., Goldie K.N. (2019). Lewy pathology in Parkinson’s disease consists of crowded organelles and lipid membranes. Nat. Neurosci..

[6] Polymeropoulos M.H., Lavedan C., Leroy E., Ide S.E., Dehejia A., Dutra A., Pike B., Root H., Rubenstein J., Boyer R. (1997). Mutation in the α-Synuclein Gene Identified in Families with Parkinson’s Disease. Science.

[7] Eliezer D., Kutluay E., Bussell R., Browne G. (2001). Conformational properties of alpha-synuclein in its free and lipid-associated states. J. Mol. Biol..

[8] Bartels T., Choi J.G., Selkoe D.J. (2011). alpha-Synuclein occurs physiologically as a helically folded tetramer that resists aggregation. Nature.

[9] Burre J., Vivona S., Diao J., Sharma M., Brunger A.T., Sudhof T.C. (2013). Properties of native brain alpha-synuclein. Nature.

[10] Giasson B.I., Murray I.V., Trojanowski J.Q., Lee V.M. (2001). A hydrophobic stretch of 12 amino acid residues in the middle of alpha-synuclein is essential for filament assembly. J. Biol. Chem..

[11] Winner B., Jappelli R., Maji S.K., Desplats P.A., Boyer L., Aigner S., Hetzer C., Loher T., Vilar M., Campioni S. (2011). In vivo demonstration that alpha-synuclein oligomers are toxic. Proc. Natl. Acad. Sci. USA.

[12] Karpinar D.P., Balija M.B., Kugler S., Opazo F., Rezaei-Ghaleh N., Wender N., Kim H.Y., Taschenberger G., Falkenburger B.H., Heise H. (2009). Pre-fibrillar alpha-synuclein variants with impaired beta-structure increase neurotoxicity in Parkinson’s disease models. EMBO J..

[13] Lau A., So R.W.L., Lau H.H.C., Sang J.C., Ruiz-Riquelme A., Fleck S.C., Stuart E., Menon S., Visanji N.P., Meisl G. (2020). alpha-Synuclein strains target distinct brain regions and cell types. Nat. Neurosci..

[14] Shahnawaz M., Mukherjee A., Pritzkow S., Mendez N., Rabadia P., Liu X., Hu B., Schmeichel A., Singer W., Wu G. (2020). Discriminating alpha-synuclein strains in Parkinson’s disease and multiple system atrophy. Nature.

[15] Peng C., Gathagan R.J., Covell D.J., Medellin C., Stieber A., Robinson J.L., Zhang B., Pitkin R.M., Olufemi M.F., Luk K.C. (2018). Cellular milieu imparts distinct pathological alpha-synuclein strains in alpha-synucleinopathies. Nature.

[16] Devine M.J., Ryten M., Vodicka P., Thomson A.J., Burdon T., Houlden H., Cavaleri F., Nagano M., Drummond N.J., Taanman J.W. (2011). Parkinson’s disease induced pluripotent stem cells with triplication of the alpha-synuclein locus. Nat. Commun..

[17] Massey A.R., Beckham J.D. (2016). Alpha-Synuclein, a Novel Viral Restriction Factor Hiding in Plain Sight. DNA Cell Biol..

[18] Chen S.G., Stribinskis V., Rane M.J., Demuth D.R., Gozal E., Roberts A.M., Jagadapillai R., Liu R., Choe K., Shivakumar B. (2016). Exposure to the Functional Bacterial Amyloid Protein Curli Enhances Alpha-Synuclein Aggregation in Aged Fischer 344 Rats and Caenorhabditis elegans. Sci. Rep..

[19] Manning-Bog A.B., McCormack A.L., Li J., Uversky V.N., Fink A.L., Di Monte D.A. (2002). The herbicide paraquat causes up-regulation and aggregation of alpha-synuclein in mice: Paraquat and alpha-synuclein. J. Biol. Chem..

[20] Kumar A., Leinisch F., Kadiiska M.B., Corbett J., Mason R.P. (2016). Formation and Implications of Alpha-Synuclein Radical in Maneb-and Paraquat-Induced Models of Parkinson’s Disease. Mol. Neurobiol..

[21] Guzman J.N., Sanchez-Padilla J., Wokosin D., Kondapalli J., Ilijic E., Schumacker P.T., Surmeier D.J. (2010). Oxidant stress evoked by pacemaking in dopaminergic neurons is attenuated by DJ-1. Nature.

[22] Surmeier D.J., Halliday G.M., Simuni T. (2017). Calcium, mitochondrial dysfunction and slowing the progression of Parkinson’s disease. Exp. Neurol..

[23] Hirsch E.C., Hunot S. (2009). Neuroinflammation in Parkinson’s disease: A target for neuroprotection?. Lancet. Neurol..

[24] Dehay B., Bourdenx M., Gorry P., Przedborski S., Vila M., Hunot S., Singleton A., Olanow C.W., Merchant K.M., Bezard E. (2015). Targeting α-synuclein for treatment of Parkinson’s disease: Mechanistic and therapeutic considerations. Lancet. Neurol..

[25] Maroteaux L., Campanelli J.T., Scheller R.H. (1988). Synuclein: A neuron-specific protein localized to the nucleus and presynaptic nerve terminal. J. Neurosci..

[26] Burre J., Sharma M., Tsetsenis T., Buchman V., Etherton M.R., Sudhof T.C. (2010). Alpha-synuclein promotes SNARE-complex assembly in vivo and in vitro. Science.

[27] Choi B.K., Choi M.G., Kim J.Y., Yang Y., Lai Y., Kweon D.H., Lee N.K., Shin Y.K. (2013). Large alpha-synuclein oligomers inhibit neuronal SNARE-mediated vesicle docking. Proc. Natl. Acad. Sci. USA.

[28] Senior S.L., Ninkina N., Deacon R., Bannerman D., Buchman V.L., Cragg S.J., Wade-Martins R. (2008). Increased striatal dopamine release and hyperdopaminergic-like behaviour in mice lacking both alpha-synuclein and gamma-synuclein. Eur. J. Neurosci..

[29] Anwar S., Peters O., Millership S., Ninkina N., Doig N., Connor-Robson N., Threlfell S., Kooner G., Deacon R.M., Bannerman D.M. (2011). Functional alterations to the nigrostriatal system in mice lacking all three members of the synuclein family. J. Neurosci..

[30] Mor D.E., Tsika E., Mazzulli J.R., Gould N.S., Kim H., Daniels M.J., Doshi S., Gupta P., Grossman J.L., Tan V.X. (2017). Dopamine induces soluble alpha-synuclein oligomers and nigrostriatal degeneration. Nat. Neurosci..

[31] Burbulla L.F., Song P., Mazzulli J.R., Zampese E., Wong Y.C., Jeon S., Santos D.P., Blanz J., Obermaier C.D., Strojny C. (2017). Dopamine oxidation mediates mitochondrial and lysosomal dysfunction in Parkinson’s disease. Science.

[32] Smith W.W., Jiang H., Pei Z., Tanaka Y., Morita H., Sawa A., Dawson V.L., Dawson T.M., Ross C.A. (2005). Endoplasmic reticulum stress and mitochondrial cell death pathways mediate A53T mutant alpha-synuclein-induced toxicity. Hum. Mol. Genet..

[33] Cooper A.A., Gitler A.D., Cashikar A., Haynes C.M., Hill K.J., Bhullar B., Liu K., Xu K., Strathearn K.E., Liu F. (2006). Alpha-synuclein blocks ER-Golgi traffic and Rab1 rescues neuron loss in Parkinson’s models. Science.

[34] Luth E.S., Stavrovskaya I.G., Bartels T., Kristal B.S., Selkoe D.J. (2014). Soluble, prefibrillar alpha-synuclein oligomers promote complex I-dependent, Ca2+-induced mitochondrial dysfunction. J. Biol. Chem..

[35] Di Maio R., Barrett P.J., Hoffman E.K., Barrett C.W., Zharikov A., Borah A., Hu X., McCoy J., Chu C.T., Burton E.A. (2016). alpha-Synuclein binds to TOM20 and inhibits mitochondrial protein import in Parkinson’s disease. Sci. Transl. Med..

[36] Choi D.H., Cristovao A.C., Guhathakurta S., Lee J., Joh T.H., Beal M.F., Kim Y.S. (2012). NADPH oxidase 1-mediated oxidative stress leads to dopamine neuron death in Parkinson’s disease. Antioxid. Redox. Signal..

[37] Indo H.P., Yen H.C., Nakanishi I., Matsumoto K., Tamura M., Nagano Y., Matsui H., Gusev O., Cornette R., Okuda T. (2015). A mitochondrial superoxide theory for oxidative stress diseases and aging. J. Clin. Biochem. Nutr..

[38] Freeman D., Cedillos R., Choyke S., Lukic Z., McGuire K., Marvin S., Burrage A.M., Sudholt S., Rana A., O’Connor C. (2013). Alpha-synuclein induces lysosomal rupture and cathepsin dependent reactive oxygen species following endocytosis. PLoS ONE.

[39] Kim C., Ho D.H., Suk J.E., You S., Michael S., Kang J., Joong Lee S., Masliah E., Hwang D., Lee H.J. (2013). Neuron-released oligomeric alpha-synuclein is an endogenous agonist of TLR2 for paracrine activation of microglia. Nat. Commun..

[40] Arotcarena M.L., Teil M., Dehay B. (2019). Autophagy in Synucleinopathy: The Overwhelmed and Defective Machinery. Cells.

[41] Paik S.R., Shin H.J., Lee J.H., Chang C.S., Kim J. (1999). Copper(II)-induced self-oligomerization of alpha-synuclein. Biochem. J..

[42] Binolfi A., Lamberto G.R., Duran R., Quintanar L., Bertoncini C.W., Souza J.M., Cervenansky C., Zweckstetter M., Griesinger C., Fernandez C.O. (2008). Site-specific interactions of Cu(II) with alpha and beta-synuclein: Bridging the molecular gap between metal binding and aggregation. J. Am. Chem. Soc..

[43] Uversky V.N., Li J., Fink A.L. (2001). Metal-triggered structural transformations, aggregation, and fibrillation of human alpha-synuclein. A possible molecular NK between Parkinson’s disease and heavy metal exposure. J. Biol. Chem..

[44] Braak H., Del Tredici K., Rub U., de Vos R.A., Jansen Steur E.N., Braak E. (2003). Staging of brain pathology related to sporadic Parkinson’s disease. Neurobiol. Aging.

[45] Kordower J.H., Chu Y., Hauser R.A., Freeman T.B., Olanow C.W. (2008). Lewy body-like pathology in long-term embryonic nigral transplants in Parkinson’s disease. Nat. Med..

[46] Li J.Y., Englund E., Holton J.L., Soulet D., Hagell P., Lees A.J., Lashley T., Quinn N.P., Rehncrona S., Bjorklund A. (2008). Lewy bodies in grafted neurons in subjects with Parkinson’s disease suggest host-to-graft disease propagation. Nat. Med..

[47] Mendez I., Vinuela A., Astradsson A., Mukhida K., Hallett P., Robertson H., Tierney T., Holness R., Dagher A., Trojanowski J.Q. (2008). Dopamine neurons implanted into people with Parkinson’s disease survive without pathology for 14 years. Nat. Med..

[48] Desplats P., Lee H.J., Bae E.J., Patrick C., Rockenstein E., Crews L., Spencer B., Masliah E., Lee S.J. (2009). Inclusion formation and neuronal cell death through neuron-to-neuron transmission of alpha-synuclein. Proc. Natl. Acad. Sci. USA.

[49] Kordower J.H., Dodiya H.B., Kordower A.M., Terpstra B., Paumier K., Madhavan L., Sortwell C., Steece-Collier K., Collier T.J. (2011). Transfer of host-derived alpha synuclein to grafted dopaminergic neurons in rat. Neurobiol. Dis..

[50] Lee H.J., Suk J.E., Bae E.J., Lee J.H., Paik S.R., Lee S.J. (2008). Assembly-dependent endocytosis and clearance of extracellular alpha-synuclein. Int. J. Biochem. Cell Biol..

[51] Luk K.C., Song C., O’Brien P., Stieber A., Branch J.R., Brunden K.R., Trojanowski J.Q., Lee V.M. (2009). Exogenous alpha-synuclein fibrils seed the formation of Lewy body-like intracellular inclusions in cultured cells. Proc. Natl. Acad. Sci. USA.

[52] Holmes B.B., DeVos S.L., Kfoury N., Li M., Jacks R., Yanamandra K., Ouidja M.O., Brodsky F.M., Marasa J., Bagchi D.P. (2013). Heparan sulfate proteoglycans mediate internalization and propagation of specific proteopathic seeds. Proc. Natl. Acad. Sci. USA.

[53] Shrivastava A.N., Redeker V., Fritz N., Pieri L., Almeida L.G., Spolidoro M., Liebmann T., Bousset L., Renner M., Lena C. (2015). alpha-synuclein assemblies sequester neuronal alpha3-Na+/K+-ATPase and impair Na+ gradient. EMBO J..

[54] Mao X., Ou M.T., Karuppagounder S.S., Kam T.I., Yin X., Xiong Y., Ge P., Umanah G.E., Brahmachari S., Shin J.H. (2016). Pathological alpha-synuclein transmission initiated by binding lymphocyte-activation gene 3. Science.

[55] Kam T.I., Mao X., Park H., Chou S.C., Karuppagounder S.S., Umanah G.E., Yun S.P., Brahmachari S., Panicker N., Chen R. (2018). Poly(ADP-ribose) drives pathologic alpha-synuclein neurodegeneration in Parkinson’s disease. Science.

[56] Freundt E.C., Maynard N., Clancy E.K., Roy S., Bousset L., Sourigues Y., Covert M., Melki R., Kirkegaard K., Brahic M. (2012). Neuron-to-neuron transmission of alpha-synuclein fibrils through axonal transport. Ann. Neurol..

[57] Brahic M., Bousset L., Bieri G., Melki R., Gitler A.D. (2016). Axonal transport and secretion of fibrillar forms of alpha-synuclein, Abeta42 peptide and HTTExon 1. Acta Neuropathol..

[58] Reyes J.F., Olsson T.T., Lamberts J.T., Devine M.J., Kunath T., Brundin P. (2015). A cell culture model for monitoring alpha-synuclein cell-to-cell transfer. Neurobiol. Dis..

[59] Danzer K.M., Kranich L.R., Ruf W.P., Cagsal-Getkin O., Winslow A.R., Zhu L., Vanderburg C.R., McLean P.J. (2012). Exosomal cell-to-cell transmission of alpha synuclein oligomers. Mol. Neurodegener..

[60] Luk K.C., Kehm V.M., Zhang B., O’Brien P., Trojanowski J.Q., Lee V.M. (2012). Intracerebral inoculation of pathological alpha-synuclein initiates a rapidly progressive neurodegenerative alpha-synucleinopathy in mice. J. Exp. Med..

[61] Masuda-Suzukake M., Nonaka T., Hosokawa M., Oikawa T., Arai T., Akiyama H., Mann D.M., Hasegawa M. (2013). Prion-like spreading of pathological alpha-synuclein in brain. Brain.

[62] Luk K.C., Kehm V., Carroll J., Zhang B., O’Brien P., Trojanowski J.Q., Lee V.M. (2012). Pathological alpha-synuclein transmission initiates Parkinson-like neurodegeneration in nontransgenic mice. Science.

[63] Mougenot A.L., Nicot S., Bencsik A., Morignat E., Verchere J., Lakhdar L., Legastelois S., Baron T. (2012). Prion-like acceleration of a synucleinopathy in a transgenic mouse model. Neurobiol. Aging.

[64] Henderson M.X., Cornblath E.J., Darwich A., Zhang B., Brown H., Gathagan R.J., Sandler R.M., Bassett D.S., Trojanowski J.Q., Lee V.M.Y. (2019). Spread of alpha-synuclein pathology through the brain connectome is modulated by selective vulnerability and predicted by network analysis. Nat. Neurosci..

[65] Chu Y., Muller S., Tavares A., Barret O., Alagille D., Seibyl J., Tamagnan G., Marek K., Luk K.C., Trojanowski J.Q. (2019). Intrastriatal alpha-synuclein fibrils in monkeys: Spreading, imaging and neuropathological changes. Brain.

[66] Recasens A., Dehay B., Bove J., Carballo-Carbajal I., Dovero S., Perez-Villalba A., Fernagut P.O., Blesa J., Parent A., Perier C. (2014). Lewy body extracts from Parkinson disease brains trigger alpha-synuclein pathology and neurodegeneration in mice and monkeys. Ann. Neurol..

[67] Kim S., Kwon S.H., Kam T.I., Panicker N., Karuppagounder S.S., Lee S., Lee J.H., Kim W.R., Kook M., Foss C.A. (2019). Transneuronal Propagation of Pathologic alpha-Synuclein from the Gut to the Brain Models Parkinson’s Disease. Neuron.

[68] Volpicelli-Daley L.A., Luk K.C., Patel T.P., Tanik S.A., Riddle D.M., Stieber A., Meaney D.F., Trojanowski J.Q., Lee V.M. (2011). Exogenous alpha-synuclein fibrils induce Lewy body pathology leading to synaptic dysfunction and neuron death. Neuron.

[69] Bousset L., Pieri L., Ruiz-Arlandis G., Gath J., Jensen P.H., Habenstein B., Madiona K., Olieric V., Bockmann A., Meier B.H. (2013). Structural and functional characterization of two alpha-synuclein strains. Nat. Commun..

[70] Hansen C., Angot E., Bergstrom A.L., Steiner J.A., Pieri L., Paul G., Outeiro T.F., Melki R., Kallunki P., Fog K. (2011). alpha-Synuclein propagates from mouse brain to grafted dopaminergic neurons and seeds aggregation in cultured human cells. J. Clin. Investig..

[71] Appel-Cresswell S., Vilarino-Guell C., Encarnacion M., Sherman H., Yu I., Shah B., Weir D., Thompson C., Szu-Tu C., Trinh J. (2013). Alpha-synuclein p.H50Q, a novel pathogenic mutation for Parkinson’s disease. Mov. Disord..

[72] Krüger R., Kuhn W., Müller T., Woitalla D., Graeber M., Kösel S., Przuntek H., Epplen J.T., Schols L., Riess O. (1998). AlaSOPro mutation in the gene encoding α-synuclein in Parkinson’s disease. Nat. Genet..

[73] Lesage S., Anheim M., Letournel F., Bousset L., Honoré A., Rozas N., Pieri L., Madiona K., Dürr A., Melki R. (2013). G51D α-synuclein mutation causes a novel Parkinsonian–pyramidal syndrome. Ann. Neurol..

[74] Pasanen P., Myllykangas L., Siitonen M., Raunio A., Kaakkola S., Lyytinen J., Tienari P.J., Pöyhönen M., Paetau A. (2014). A novel α-synuclein mutation A53E associated with atypical multiple system atrophy and Parkinson’s disease-type pathology. Neurobiol. Aging.

[75] Proukakis C., Dudzik C.G., Brier T., MacKay D.S., Cooper J.M., Millhauser G.L., Houlden H., Schapira A.H. (2013). A novel α-synuclein missense mutation in Parkinson disease. Neurology.

[76] Zarranz J.J., Alegre J., Gomez-Esteban J.C., Lezcano E., Ros R., Ampuero I., Vidal L., Hoenicka J., Rodriguez O., Atares B. (2004). The new mutation, E46K, of alpha-synuclein causes Parkinson and Lewy body dementia. Ann. Neurol..

[77] Yoshino H., Hirano M., Stoessl A.J., Imamichi Y., Ikeda A., Li Y., Funayama M., Yamada I., Nakamura Y., Sossi V. (2017). Homozygous alpha-synuclein p.A53V in familial Parkinson’s disease. Neurobiol. Aging.

[78] Chartier-Harlin M.C., Kachergus J., Roumier C., Mouroux V., Douay X., Lincoln S., Levecque C., Larvor L., Andrieux J., Hulihan M. (2004). Alpha-synuclein locus duplication as a cause of familial Parkinson’s disease. Lancet.

[79] Singleton A.B., Farrer M., Johnson J., Singleton A., Hague S., Kachergus J., Hulihan M., Peuralinna T., Dutra A., Nussbaum R. (2003). α-Synuclein Locus Triplication Causes Parkinson’s Disease. Science.

[80] Eriksen J.L., Przedborski S., Petrucelli L. (2005). Gene dosage and pathogenesis of Parkinson’s disease. Trends Mol. Med..

[81] Farrer M., Kachergus J., Forno L., Lincoln S., Wang D.-S., Hulihan M., Maraganore D., Gwinn-Hardy K., Wszolek Z., Dickson D. (2004). Comparison of kindreds with parkinsonism and α-synuclein genomic multiplications. Ann. Neurol..

[82] Fire A., Xu S., Montgomery M.K., Kostas S.A., Driver S.E., Mello C.C. (1998). Potent and specific genetic interference by double-stranded RNA in Caenorhabditis elegans. Nature.

[83] Scherr M., Eder M. (2007). Gene Silencing by Small Regulatory RNAs in Mammalian Cells. Cell Cycle.

[84] Sapru M.K., Yates J.W., Hogan S., Jiang L., Halter J., Bohn M.C. (2006). Silencing of human alpha-synuclein in vitro and in rat brain using lentiviral-mediated RNAi. Exp. Neurol..

[85] Hayashita-Kinoh H., Yamada M., Yokota T., Mizuno Y., Mochizuki H. (2006). Down-regulation of alpha-synuclein expression can rescue dopaminergic cells from cell death in the substantia nigra of Parkinson’s disease rat model. Biochem. Biophys. Res. Commun..

[86] Lewis J., Melrose H., Bumcrot D., Hope A., Zehr C., Lincoln S., Braithwaite A., He Z., Ogholikhan S., Hinkle K. (2008). In vivo silencing of alpha-synuclein using naked siRNA. Mol. Neurodegener..

[87] Junn E., Lee K.-W., Jeong B.S., Chan T.W., Im J.-Y., Mouradian M.M. (2009). Repression of α-synuclein expression and toxicity by microRNA-7. Proc. Natl. Acad. Sci. USA.

[88] Doxakis E. (2010). Post-transcriptional regulation of alpha-synuclein expression by mir-7 and mir-153. J. Biol. Chem..

[89] Gorbatyuk O.S., Li S., Nash K., Gorbatyuk M., Lewin A.S., Sullivan L.F., Mandel R.J., Chen W., Meyers C., Manfredsson F.P. (2010). In vivo RNAi-mediated alpha-synuclein silencing induces nigrostriatal degeneration. Mol. Therapy.

[90] Khodr C.E., Sapru M.K., Pedapati J., Han Y., West N.C., Kells A.P., Bankiewicz K.S., Bohn M.C. (2011). An alpha-synuclein AAV gene silencing vector ameliorates a behavioral deficit in a rat model of Parkinson’s disease, but displays toxicity in dopamine neurons. Brain Res..

[91] Han Y., Khodr C.E., Sapru M.K., Pedapati J., Bohn M.C. (2011). A microRNA embedded AAV alpha-synuclein gene silencing vector for dopaminergic neurons. Brain Res..

[92] Khodr C.E., Becerra A., Han Y., Bohn M.C. (2014). Targeting alpha-synuclein with a microRNA-embedded silencing vector in the rat substantia nigra: Positive and negative effects. Brain Res..

[93] Collier T.J., Redmond D.E., Steece-Collier K., Lipton J.W., Manfredsson F.P. (2016). Is Alpha-Synuclein Loss-of-Function a Contributor to Parkinsonian Pathology? Evidence from Non-human Primates. Front. Neurosci..

[94] Benskey M.J., Sellnow R.C., Sandoval I.M., Sortwell C.E., Lipton J.W., Manfredsson F.P. (2018). Silencing Alpha Synuclein in Mature Nigral Neurons Results in Rapid Neuroinflammation and Subsequent Toxicity. Front. Mol. Neurosci..

[95] Zharikov A., Bai Q., De Miranda B.R., Van Laar A., Greenamyre J.T., Burton E.A. (2019). Long-term RNAi knockdown of alpha-synuclein in the adult rat substantia nigra without neurodegeneration. Neurobiol. Dis..

[96] McCormack A.L., Mak S.K., Henderson J.M., Bumcrot D., Farrer M.J., Di Monte D.A. (2010). Alpha-synuclein suppression by targeted small interfering RNA in the primate substantia nigra. PLoS ONE.

[97] Chen L., Huang E., Wang H., Qiu P., Liu C. (2013). RNA interference targeting alpha-synuclein attenuates methamphetamine-induced neurotoxicity in SH-SY5Y cells. Brain Res..

[98] Takahashi M., Suzuki M., Fukuoka M., Fujikake N., Watanabe S., Murata M., Wada K., Nagai Y., Hohjoh H. (2015). Normalization of Overexpressed alpha-Synuclein Causing Parkinson’s Disease By a Moderate Gene Silencing With RNA Interference. Mol. Therapy Nucleic. Acids.

[99] Zharikov A.D., Cannon J.R., Tapias V., Bai Q., Horowitz M.P., Shah V., El Ayadi A., Hastings T.G., Greenamyre J.T., Burton E.A. (2015). shRNA targeting alpha-synuclein prevents neurodegeneration in a Parkinson’s disease model. J. Clin. Investig..

[100] Sibley C.R., Wood M.J. (2011). Identification of allele-specific RNAi effectors targeting genetic forms of Parkinson’s disease. PLoS ONE.

[101] Cooper J.M., Wiklander P.B., Nordin J.Z., Al-Shawi R., Wood M.J., Vithlani M., Schapira A.H., Simons J.P., El-Andaloussi S., Alvarez-Erviti L. (2014). Systemic exosomal siRNA delivery reduced alpha-synuclein aggregates in brains of transgenic mice. Mov. Disord..

[102] Schlich M., Longhena F., Faustini G., O’Driscoll C.M., Sinico C., Fadda A.M., Bellucci A., Lai F. (2017). Anionic liposomes for small interfering ribonucleic acid (siRNA) delivery to primary neuronal cells: Evaluation of alpha-synuclein knockdown efficacy. Nano Res..

[103] Xhima K., Nabbouh F., Hynynen K., Aubert I., Tandon A. (2018). Noninvasive delivery of an alpha-synuclein gene silencing vector with magnetic resonance-guided focused ultrasound. Mov. Disord..

[104] Alarcon-Aris D., Recasens A., Galofre M., Carballo-Carbajal I., Zacchi N., Ruiz-Bronchal E., Pavia-Collado R., Chica R., Ferres-Coy A., Santos M. (2018). Selective alpha-Synuclein Knockdown in Monoamine Neurons by Intranasal Oligonucleotide Delivery: Potential Therapy for Parkinson’s Disease. Mol. Therapy.

[105] Javed H., Menon S.A., Al-Mansoori K.M., Al-Wandi A., Majbour N.K., Ardah M.T., Varghese S., Vaikath N.N., Haque M.E., Azzouz M. (2016). Development of Nonviral Vectors Targeting the Brain as a Therapeutic Approach For Parkinson’s Disease and Other Brain Disorders. Mol. Therapy.

[106] Spencer B., Trinh I., Rockenstein E., Mante M., Florio J., Adame A., El-Agnaf O.M.A., Kim C., Masliah E., Rissman R.A. (2019). Systemic peptide mediated delivery of an siRNA targeting alpha-syn in the CNS ameliorates the neurodegenerative process in a transgenic model of Lewy body disease. Neurobiol. Dis..

[107] Dermentzaki G., Paschalidis N., Politis P.K., Stefanis L. (2016). Complex Effects of the ZSCAN21 Transcription Factor on Transcriptional Regulation of alpha-Synuclein in Primary Neuronal Cultures and in Vivo. J. Biol. Chem..

[108] Lassot I., Mora S., Lesage S., Zieba B.A., Coque E., Condroyer C., Bossowski J.P., Mojsa B., Marelli C., Soulet C. (2018). The E3 Ubiquitin Ligases TRIM17 and TRIM41 Modulate alpha-Synuclein Expression by Regulating ZSCAN21. Cell Rep..

[109] Mittal S., Bjornevik K., Im D.S., Flierl A., Dong X., Locascio J.J., Abo K.M., Long E., Jin M., Xu B. (2017). beta2-Adrenoreceptor is a regulator of the alpha-synuclein gene driving risk of Parkinson’s disease. Science.

[110] Fujiwara H., Hasegawa M., Dohmae N., Kawashima A., Masliah E., Goldberg M.S., Shen J., Takio K., Iwatsubo T. (2002). alpha-Synuclein is phosphorylated in synucleinopathy lesions. Nat. Cell Biol..

[111] Anderson J.P., Walker D.E., Goldstein J.M., de Laat R., Banducci K., Caccavello R.J., Barbour R., Huang J., Kling K., Lee M. (2006). Phosphorylation of Ser-129 is the dominant pathological modification of alpha-synuclein in familial and sporadic Lewy body disease. J. Biol. Chem..

[112] Okochi M., Walter J., Koyama A., Nakajo S., Baba M., Iwatsubo T., Meijer L., Kahle P.J., Haass C. (2000). Constitutive phosphorylation of the Parkinson’s disease associated alpha-synuclein. J. Biol. Chem..

[113] Pronin A.N., Morris A.J., Surguchov A., Benovic J.L. (2000). Synucleins are a novel class of substrates for G protein-coupled receptor kinases. J. Biol. Chem..

[114] Negro A., Brunati A.M., Donella-Deana A., Massimino M.L., Pinna L.A. (2002). Multiple phosphorylation of alpha-synuclein by protein tyrosine kinase Syk prevents eosin-induced aggregation. FASEB J..

[115] Smith W.W., Margolis R.L., Li X., Troncoso J.C., Lee M.K., Dawson V.L., Dawson T.M., Iwatsubo T., Ross C.A. (2005). Alpha-synuclein phosphorylation enhances eosinophilic cytoplasmic inclusion formation in SH-SY5Y cells. J. Neurosci..

[116] Azeredo da Silveira S., Schneider B.L., Cifuentes-Diaz C., Sage D., Abbas-Terki T., Iwatsubo T., Unser M., Aebischer P. (2009). Phosphorylation does not prompt, nor prevent, the formation of alpha-synuclein toxic species in a rat model of Parkinson’s disease. Hum. Mol. Genet..

[117] Mbefo M.K., Paleologou K.E., Boucharaba A., Oueslati A., Schell H., Fournier M., Olschewski D., Yin G., Zweckstetter M., Masliah E. (2010). Phosphorylation of synucleins by members of the Polo-like kinase family. J. Biol. Chem..

[118] Chen L., Feany M.B. (2005). Alpha-synuclein phosphorylation controls neurotoxicity and inclusion formation in a Drosophila model of Parkinson disease. Nat. Neurosci..

[119] Chen L., Periquet M., Wang X., Negro A., McLean P.J., Hyman B.T., Feany M.B. (2009). Tyrosine and serine phosphorylation of alpha-synuclein have opposing effects on neurotoxicity and soluble oligomer formation. J. Clin. Investig..

[120] Arawaka S., Wada M., Goto S., Karube H., Sakamoto M., Ren C.H., Koyama S., Nagasawa H., Kimura H., Kawanami T. (2006). The role of G-protein-coupled receptor kinase 5 in pathogenesis of sporadic Parkinson’s disease. J. Neurosci..

[121] Inglis K.J., Chereau D., Brigham E.F., Chiou S.S., Schobel S., Frigon N.L., Yu M., Caccavello R.J., Nelson S., Motter R. (2009). Polo-like kinase 2 (PLK2) phosphorylates alpha-synuclein at serine 129 in central nervous system. J. Biol. Chem..

[122] Waxman E.A., Giasson B.I. (2011). Induction of intracellular tau aggregation is promoted by alpha-synuclein seeds and provides novel insights into the hyperphosphorylation of tau. J. Neurosci..

[123] Oueslati A., Schneider B.L., Aebischer P., Lashuel H.A. (2013). Polo-like kinase 2 regulates selective autophagic alpha-synuclein clearance and suppresses its toxicity in vivo. Proc. Natl. Acad. Sci. USA.

[124] Aubele D.L., Hom R.K., Adler M., Galemmo R.A., Bowers S., Truong A.P., Pan H., Beroza P., Neitz R.J., Yao N. (2013). Selective and brain-permeable polo-like kinase-2 (Plk-2) inhibitors that reduce alpha-synuclein phosphorylation in rat brain. ChemMedChem.

[125] Rodriguez-Nogales C., Garbayo E., Martinez-Valbuena I., Sebastian V., Luquin M.R., Blanco-Prieto M.J. (2016). Development and characterization of polo-like kinase 2 loaded nanoparticles-A novel strategy for (serine-129) phosphorylation of alpha-synuclein. Int. J. Pharm..

[126] Lee K.W., Chen W., Junn E., Im J.Y., Grosso H., Sonsalla P.K., Feng X., Ray N., Fernandez J.R., Chao Y. (2011). Enhanced phosphatase activity attenuates alpha-synucleinopathy in a mouse model. J. Neurosci..

[127] Waxman E.A., Giasson B.I. (2008). Specificity and regulation of casein kinase-mediated phosphorylation of alpha-synuclein. J. Neuropathol. Exp. Neurol..

[128] Lou H., Montoya S.E., Alerte T.N., Wang J., Wu J., Peng X., Hong C.S., Friedrich E.E., Mader S.A., Pedersen C.J. (2010). Serine 129 phosphorylation reduces the ability of alpha-synuclein to regulate tyrosine hydroxylase and protein phosphatase 2A in vitro and in vivo. J. Biol. Chem..

[129] Perez-Revuelta B.I., Hettich M.M., Ciociaro A., Rotermund C., Kahle P.J., Krauss S., Di Monte D.A. (2014). Metformin lowers Ser-129 phosphorylated alpha-synuclein levels via mTOR-dependent protein phosphatase 2A activation. Cell Death Dis..

[130] Katila N., Bhurtel S., Shadfar S., Srivastav S., Neupane S., Ojha U., Jeong G.S., Choi D.Y. (2017). Metformin lowers alpha-synuclein phosphorylation and upregulates neurotrophic factor in the MPTP mouse model of Parkinson’s disease. Neuropharmacology.

[131] Tolstykh T., Lee J., Vafai S., Stock J.B. (2000). Carboxyl methylation regulates phosphoprotein phosphatase 2A by controlling the association of regulatory B subunits. EMBO J..

[132] Wu J., Tolstykh T., Lee J., Boyd K., Stock J.B., Broach J.R. (2000). Carboxyl methylation of the phosphoprotein phosphatase 2A catalytic subunit promotes its functional association with regulatory subunits in vivo. EMBO J..

[133] Yan R., Zhang J., Park H.J., Park E.S., Oh S., Zheng H., Junn E., Voronkov M., Stock J.B., Mouradian M.M. (2018). Synergistic neuroprotection by coffee components eicosanoyl-5-hydroxytryptamide and caffeine in models of Parkinson’s disease and DLB. Proc. Natl. Acad. Sci. USA.

[134] Baba M., Nakajo S., Tu P.H., Tomita T., Nakaya K., Lee V.M., Trojanowski J.Q., Iwatsubo T. (1998). Aggregation of alpha-synuclein in Lewy bodies of sporadic Parkinson’s disease and dementia with Lewy bodies. Am. J. Pathol.

[135] Crowther R.A., Jakes R., Spillantini M.G., Goedert M. (1998). Synthetic filaments assembled from C-terminally truncated alpha-synuclein. FEBS Lett..

[136] Campbell B.C., McLean C.A., Culvenor J.G., Gai W.P., Blumbergs P.C., Jakala P., Beyreuther K., Masters C.L., Li Q.X. (2001). The solubility of alpha-synuclein in multiple system atrophy differs from that of dementia with Lewy bodies and Parkinson’s disease. J. Neurochem..

[137] Li W., West N., Colla E., Pletnikova O., Troncoso J.C., Marsh L., Dawson T.M., Jakala P., Hartmann T., Price D.L. (2005). Aggregation promoting C-terminal truncation of alpha-synuclein is a normal cellular process and is enhanced by the familial Parkinson’s disease-linked mutations. Proc. Natl. Acad. Sci. USA.

[138] Murray I.V., Giasson B.I., Quinn S.M., Koppaka V., Axelsen P.H., Ischiropoulos H., Trojanowski J.Q., Lee V.M. (2003). Role of alpha-synuclein carboxy-terminus on fibril formation in vitro. Biochemistry.

[139] Hoyer W., Cherny D., Subramaniam V., Jovin T.M. (2004). Impact of the acidic C-terminal region comprising amino acids 109-140 on alpha-synuclein aggregation in vitro. Biochemistry.

[140] Liu C.W., Giasson B.I., Lewis K.A., Lee V.M., Demartino G.N., Thomas P.J. (2005). A precipitating role for truncated alpha-synuclein and the proteasome in alpha-synuclein aggregation: Implications for pathogenesis of Parkinson disease. J. Biol. Chem..

[141] Ulusoy A., Febbraro F., Jensen P.H., Kirik D., Romero-Ramos M. (2010). Co-expression of C-terminal truncated alpha-synuclein enhances full-length alpha-synuclein-induced pathology. Eur. J. Neurosci..

[142] Mishizen-Eberz A.J., Guttmann R.P., Giasson B.I., Day G.A., Hodara R., Ischiropoulos H., Lee V.M., Trojanowski J.Q., Lynch D.R. (2003). Distinct cleavage patterns of normal and pathologic forms of alpha-synuclein by calpain I in vitro. J. Neurochem..

[143] Mishizen-Eberz A.J., Norris E.H., Giasson B.I., Hodara R., Ischiropoulos H., Lee V.M., Trojanowski J.Q., Lynch D.R. (2005). Cleavage of alpha-synuclein by calpain: Potential role in degradation of fibrillized and nitrated species of alpha-synuclein. Biochemistry.

[144] Dufty B.M., Warner L.R., Hou S.T., Jiang S.X., Gomez-Isla T., Leenhouts K.M., Oxford J.T., Feany M.B., Masliah E., Rohn T.T. (2007). Calpain-cleavage of alpha-synuclein: Connecting proteolytic processing to disease-linked aggregation. Am. J. Pathol.

[145] Mahul-Mellier A.-L., Altay F., Burtscher J., Maharjan N., Ait Bouziad N., Chiki A., Vingill S., Wade-Martins R., Holton J., Strand C. (2018). The making of a Lewy body: The role of alpha-synuclein post-fibrillization modifications in regulating the formation and the maturation of pathological inclusions. bioRxiv.

[146] Diepenbroek M., Casadei N., Esmer H., Saido T.C., Takano J., Kahle P.J., Nixon R.A., Rao M.V., Melki R., Pieri L. (2014). Overexpression of the calpain-specific inhibitor calpastatin reduces human alpha-Synuclein processing, aggregation and synaptic impairment in [A30P]alphaSyn transgenic mice. Hum. Mol. Genet..

[147] Games D., Valera E., Spencer B., Rockenstein E., Mante M., Adame A., Patrick C., Ubhi K., Nuber S., Sacayon P. (2014). Reducing C-terminal-truncated alpha-synuclein by immunotherapy attenuates neurodegeneration and propagation in Parkinson’s disease-like models. J. Neurosci..

[148] Wang W., Nguyen L.T., Burlak C., Chegini F., Guo F., Chataway T., Ju S., Fisher O.S., Miller D.W., Datta D. (2016). Caspase-1 causes truncation and aggregation of the Parkinson’s disease-associated protein alpha-synuclein. Proc. Natl. Acad. Sci. USA.

[149] Iwata A., Maruyama M., Akagi T., Hashikawa T., Kanazawa I., Tsuji S., Nukina N. (2003). Alpha-synuclein degradation by serine protease neurosin: Implication for pathogenesis of synucleinopathies. Hum. Mol. Genet..

[150] Kasai T., Tokuda T., Yamaguchi N., Watanabe Y., Kametani F., Nakagawa M., Mizuno T. (2008). Cleavage of normal and pathological forms of alpha-synuclein by neurosin in vitro. Neurosci. Lett..

[151] Sevlever D., Jiang P., Yen S.H. (2008). Cathepsin D is the main lysosomal enzyme involved in the degradation of alpha-synuclein and generation of its carboxy-terminally truncated species. Biochemistry.

[152] Sung J.Y., Park S.M., Lee C.H., Um J.W., Lee H.J., Kim J., Oh Y.J., Lee S.T., Paik S.R., Chung K.C. (2005). Proteolytic cleavage of extracellular secreted {alpha}-synuclein via matrix metalloproteinases. J. Biol. Chem..

[153] Choi D.H., Kim Y.J., Kim Y.G., Joh T.H., Beal M.F., Kim Y.S. (2011). Role of matrix metalloproteinase 3-mediated alpha-synuclein cleavage in dopaminergic cell death. J. Biol. Chem..

[154] Hart G.W., Housley M.P., Slawson C. (2007). Cycling of O-linked beta-N-acetylglucosamine on nucleocytoplasmic proteins. Nature.

[155] Cole R.N., Hart G.W. (2001). Cytosolic O-glycosylation is abundant in nerve terminals. J. Neurochem..

[156] Wang Z., Udeshi N.D., O’Malley M., Shabanowitz J., Hunt D.F., Hart G.W. (2010). Enrichment and site mapping of O-linked N-acetylglucosamine by a combination of chemical/enzymatic tagging, photochemical cleavage, and electron transfer dissociation mass spectrometry. Mol. Cell Proteom..

[157] Alfaro J.F., Gong C.X., Monroe M.E., Aldrich J.T., Clauss T.R., Purvine S.O., Wang Z., Camp D.G., Shabanowitz J., Stanley P. (2012). Tandem mass spectrometry identifies many mouse brain O-GlcNAcylated proteins including EGF domain-specific O-GlcNAc transferase targets. Proc. Natl. Acad. Sci. USA.

[158] Marotta N.P., Lin Y.H., Lewis Y.E., Ambroso M.R., Zaro B.W., Roth M.T., Arnold D.B., Langen R., Pratt M.R. (2015). O-GlcNAc modification blocks the aggregation and toxicity of the protein alpha-synuclein associated with Parkinson’s disease. Nat. Chem..

[159] Lewis Y.E., Galesic A., Levine P.M., De Leon C.A., Lamiri N., Brennan C.K., Pratt M.R. (2017). O-GlcNAcylation of alpha-Synuclein at Serine 87 Reduces Aggregation without Affecting Membrane Binding. ACS Chem. Biol..

[160] Levine P.M., De Leon C.A., Galesic A., Balana A., Marotta N.P., Lewis Y.E., Pratt M.R. (2017). O-GlcNAc modification inhibits the calpain-mediated cleavage of alpha-synuclein. Bioorg. Med. Chem..

[161] Levine P.M., Galesic A., Balana A.T., Mahul-Mellier A.L., Navarro M.X., De Leon C.A., Lashuel H.A., Pratt M.R. (2019). alpha-Synuclein O-GlcNAcylation alters aggregation and toxicity, revealing certain residues as potential inhibitors of Parkinson’s disease. Proc. Natl. Acad. Sci. USA.

[162] Selnick H.G., Hess J.F., Tang C., Liu K., Schachter J.B., Ballard J.E., Marcus J., Klein D.J., Wang X., Pearson M. (2019). Discovery of MK-8719, a Potent O-GlcNAcase Inhibitor as a Potential Treatment for Tauopathies. J. Med. Chem..

[163] Gomez-Tortosa E., Newell K., Irizarry M.C., Sanders J.L., Hyman B.T. (2000). alpha-Synuclein immunoreactivity in dementia with Lewy bodies: Morphological staging and comparison with ubiquitin immunostaining. Acta Neuropathol..

[164] Hasegawa M., Fujiwara H., Nonaka T., Wakabayashi K., Takahashi H., Lee V.M., Trojanowski J.Q., Mann D., Iwatsubo T. (2002). Phosphorylated alpha-synuclein is ubiquitinated in alpha-synucleinopathy lesions. J. Biol. Chem..

[165] Sampathu D.M., Giasson B.I., Pawlyk A.C., Trojanowski J.Q., Lee V.M. (2003). Ubiquitination of alpha-synuclein is not required for formation of pathological inclusions in alpha-synucleinopathies. Am. J. Pathol..

[166] Tofaris G.K., Razzaq A., Ghetti B., Lilley K.S., Spillantini M.G. (2003). Ubiquitination of alpha-synuclein in Lewy bodies is a pathological event not associated with impairment of proteasome function. J. Biol. Chem..

[167] Liu X., Hebron M., Shi W., Lonskaya I., Moussa C.E. (2019). Ubiquitin specific protease-13 independently regulates parkin ubiquitination and alpha-synuclein clearance in alpha-synucleinopathies. Hum. Mol. Genet..

[168] Shin Y., Klucken J., Patterson C., Hyman B.T., McLean P.J. (2005). The co-chaperone carboxyl terminus of Hsp70-interacting protein (CHIP) mediates alpha-synuclein degradation decisions between proteasomal and lysosomal pathways. J. Biol. Chem..

[169] Tetzlaff J.E., Putcha P., Outeiro T.F., Ivanov A., Berezovska O., Hyman B.T., McLean P.J. (2008). CHIP targets toxic alpha-Synuclein oligomers for degradation. J. Biol. Chem..

[170] Lee J.T., Wheeler T.C., Li L., Chin L.S. (2008). Ubiquitination of alpha-synuclein by Siah-1 promotes alpha-synuclein aggregation and apoptotic cell death. Hum. Mol. Genet..

[171] Tofaris G.K., Kim H.T., Hourez R., Jung J.W., Kim K.P., Goldberg A.L. (2011). Ubiquitin ligase Nedd4 promotes alpha-synuclein degradation by the endosomal-lysosomal pathway. Proc. Natl. Acad. Sci. USA.

[172] Davies S.E., Hallett P.J., Moens T., Smith G., Mangano E., Kim H.T., Goldberg A.L., Liu J.L., Isacson O., Tofaris G.K. (2014). Enhanced ubiquitin-dependent degradation by Nedd4 protects against alpha-synuclein accumulation and toxicity in animal models of Parkinson’s disease. Neurobiol. Dis..

[173] Hejjaoui M., Haj-Yahya M., Kumar K.S., Brik A., Lashuel H.A. (2011). Towards elucidation of the role of ubiquitination in the pathogenesis of Parkinson’s disease with semisynthetic ubiquitinated alpha-synuclein. Angew Chem. Int. Ed. Engl..

[174] Meier F., Abeywardana T., Dhall A., Marotta N.P., Varkey J., Langen R., Chatterjee C., Pratt M.R. (2012). Semisynthetic, site-specific ubiquitin modification of alpha-synuclein reveals differential effects on aggregation. J. Am. Chem. Soc..

[175] Haj-Yahya M., Fauvet B., Herman-Bachinsky Y., Hejjaoui M., Bavikar S.N., Karthikeyan S.V., Ciechanover A., Lashuel H.A., Brik A. (2013). Synthetic polyubiquitinated alpha-Synuclein reveals important insights into the roles of the ubiquitin chain in regulating its pathophysiology. Proc. Natl. Acad. Sci. USA.

[176] Rott R., Szargel R., Shani V., Hamza H., Savyon M., Abd Elghani F., Bandopadhyay R., Engelender S. (2017). SUMOylation and ubiquitination reciprocally regulate alpha-synuclein degradation and pathological aggregation. Proc. Natl. Acad. Sci. USA.

[177] Krumova P., Meulmeester E., Garrido M., Tirard M., Hsiao H.H., Bossis G., Urlaub H., Zweckstetter M., Kugler S., Melchior F. (2011). Sumoylation inhibits alpha-synuclein aggregation and toxicity. J. Cell Biol..

[178] Abeywardana T., Pratt M.R. (2015). Extent of inhibition of alpha-synuclein aggregation in vitro by SUMOylation is conjugation site- and SUMO isoform-selective. Biochemistry.

[179] Watson M.D., Lee J.C. (2019). N-Terminal Acetylation Affects alpha-Synuclein Fibril Polymorphism. Biochemistry.

[180] Outeiro T.F., Kontopoulos E., Altmann S.M., Kufareva I., Strathearn K.E., Amore A.M., Volk C.B., Maxwell M.M., Rochet J.C., McLean P.J. (2007). Sirtuin 2 inhibitors rescue alpha-synuclein-mediated toxicity in models of Parkinson’s disease. Science.

[181] de Oliveira R.M., Vicente Miranda H., Francelle L., Pinho R., Szego E.M., Martinho R., Munari F., Lazaro D.F., Moniot S., Guerreiro P. (2017). The mechanism of sirtuin 2-mediated exacerbation of alpha-synuclein toxicity in models of Parkinson disease. PLoS Biol..

[182] Richarme G., Mihoub M., Dairou J., Bui L.C., Leger T., Lamouri A. (2015). Parkinsonism-associated protein DJ-1/Park7 is a major protein deglycase that repairs methylglyoxal- and glyoxal-glycated cysteine, arginine, and lysine residues. J. Biol. Chem..

[183] Vicente Miranda H., Szego E.M., Oliveira L.M.A., Breda C., Darendelioglu E., de Oliveira R.M., Ferreira D.G., Gomes M.A., Rott R., Oliveira M. (2017). Glycation potentiates alpha-synuclein-associated neurodegeneration in synucleinopathies. Brain.

[184] Giasson B.I., Duda J.E., Murray I.V., Chen Q., Souza J.M., Hurtig H.I., Ischiropoulos H., Trojanowski J.Q., Lee V.M. (2000). Oxidative damage linked to neurodegeneration by selective alpha-synuclein nitration in synucleinopathy lesions. Science.

[185] Sevcsik E., Trexler A.J., Dunn J.M., Rhoades E. (2011). Allostery in a disordered protein: Oxidative modifications to alpha-synuclein act distally to regulate membrane binding. J. Am. Chem. Soc..

[186] Burai R., Ait-Bouziad N., Chiki A., Lashuel H.A. (2015). Elucidating the Role of Site-Specific Nitration of alpha-Synuclein in the Pathogenesis of Parkinson’s Disease via Protein Semisynthesis and Mutagenesis. J. Am. Chem. Soc..

[187] Hodara R., Norris E.H., Giasson B.I., Mishizen-Eberz A.J., Lynch D.R., Lee V.M., Ischiropoulos H. (2004). Functional consequences of alpha-synuclein tyrosine nitration: Diminished binding to lipid vesicles and increased fibril formation. J. Biol. Chem..

[188] Danielson S.R., Held J.M., Schilling B., Oo M., Gibson B.W., Andersen J.K. (2009). Preferentially increased nitration of alpha-synuclein at tyrosine-39 in a cellular oxidative model of Parkinson’s disease. Anal. Chem..

[189] Masliah E., Rockenstein E., Mante M., Crews L., Spencer B., Adame A., Patrick C., Trejo M., Ubhi K., Rohn T.T. (2011). Passive immunization reduces behavioral and neuropathological deficits in an alpha-synuclein transgenic model of Lewy body disease. PLoS ONE.

[190] Schenk D.B., Koller M., Ness D.K., Griffith S.G., Grundman M., Zago W., Soto J., Atiee G., Ostrowitzki S., Kinney G.G. (2017). First-in-human assessment of PRX002, an anti-alpha-synuclein monoclonal antibody, in healthy volunteers. Mov. Disord..

[191] Jankovic J., Goodman I., Safirstein B., Marmon T.K., Schenk D.B., Koller M., Zago W., Ness D.K., Griffith S.G., Grundman M. (2018). Safety and Tolerability of Multiple Ascending Doses of PRX002/RG7935, an Anti-alpha-Synuclein Monoclonal Antibody, in Patients With Parkinson Disease: A Randomized Clinical Trial. JAMA Neurol..

[192] Bae E.J., Lee H.J., Rockenstein E., Ho D.H., Park E.B., Yang N.Y., Desplats P., Masliah E., Lee S.J. (2012). Antibody-aided clearance of extracellular alpha-synuclein prevents cell-to-cell aggregate transmission. J. Neurosci..

[193] Spencer B., Valera E., Rockenstein E., Overk C., Mante M., Adame A., Zago W., Seubert P., Barbour R., Schenk D. (2017). Anti-alpha-synuclein immunotherapy reduces alpha-synuclein propagation in the axon and degeneration in a combined viral vector and transgenic model of synucleinopathy. Acta Neuropathol. Commun..

[194] Schofield D.J., Irving L., Calo L., Bogstedt A., Rees G., Nuccitelli A., Narwal R., Petrone M., Roberts J., Brown L. (2019). Preclinical development of a high affinity alpha-synuclein antibody, MEDI1341, that can enter the brain, sequester extracellular alpha-synuclein and attenuate alpha-synuclein spreading in vivo. Neurobiol. Dis..

[195] Tran H.T., Chung C.H., Iba M., Zhang B., Trojanowski J.Q., Luk K.C., Lee V.M. (2014). Alpha-synuclein immunotherapy blocks uptake and templated propagation of misfolded alpha-synuclein and neurodegeneration. Cell Rep..

[196] Shahaduzzaman M., Nash K., Hudson C., Sharif M., Grimmig B., Lin X., Bai G., Liu H., Ugen K.E., Cao C. (2015). Anti-human alpha-synuclein N-terminal peptide antibody protects against dopaminergic cell death and ameliorates behavioral deficits in an AAV-alpha-synuclein rat model of Parkinson’s disease. PLoS ONE.

[197] Weihofen A., Liu Y., Arndt J.W., Huy C., Quan C., Smith B.A., Baeriswyl J.L., Cavegn N., Senn L., Su L. (2019). Development of an aggregate-selective, human-derived alpha-synuclein antibody BIIB054 that ameliorates disease phenotypes in Parkinson’s disease models. Neurobiol. Dis..

[198] Brys M., Fanning L., Hung S., Ellenbogen A., Penner N., Yang M., Welch M., Koenig E., David E., Fox T. (2019). Randomized phase I clinical trial of anti-alpha-synuclein antibody BIIB054. Mov. Disord..

[199] Nasstrom T., Goncalves S., Sahlin C., Nordstrom E., Screpanti Sundquist V., Lannfelt L., Bergstrom J., Outeiro T.F., Ingelsson M. (2011). Antibodies against alpha-synuclein reduce oligomerization in living cells. PLoS ONE.

[200] Fagerqvist T., Lindstrom V., Nordstrom E., Lord A., Tucker S.M., Su X., Sahlin C., Kasrayan A., Andersson J., Welander H. (2013). Monoclonal antibodies selective for alpha-synuclein oligomers/protofibrils recognize brain pathology in Lewy body disorders and alpha-synuclein transgenic mice with the disease-causing A30P mutation. J. Neurochem..

[201] Lindstrom V., Fagerqvist T., Nordstrom E., Eriksson F., Lord A., Tucker S., Andersson J., Johannesson M., Schell H., Kahle P.J. (2014). Immunotherapy targeting alpha-synuclein protofibrils reduced pathology in (Thy-1)-h[A30P] alpha-synuclein mice. Neurobiol. Dis..

[202] El-Agnaf O., Overk C., Rockenstein E., Mante M., Florio J., Adame A., Vaikath N., Majbour N., Lee S.J., Kim C. (2017). Differential effects of immunotherapy with antibodies targeting alpha-synuclein oligomers and fibrils in a transgenic model of synucleinopathy. Neurobiol. Dis..

[203] Masliah E., Rockenstein E., Adame A., Alford M., Crews L., Hashimoto M., Seubert P., Lee M., Goldstein J., Chilcote T. (2005). Effects of alpha-synuclein immunization in a mouse model of Parkinson’s disease. Neuron.

[204] Ghochikyan A., Petrushina I., Davtyan H., Hovakimyan A., Saing T., Davtyan A., Cribbs D.H., Agadjanyan M.G. (2014). Immunogenicity of epitope vaccines targeting different B cell antigenic determinants of human alpha-synuclein: Feasibility study. Neurosci. Lett..

[205] Mandler M., Valera E., Rockenstein E., Weninger H., Patrick C., Adame A., Santic R., Meindl S., Vigl B., Smrzka O. (2014). Next-generation active immunization approach for synucleinopathies: Implications for Parkinson’s disease clinical trials. Acta Neuropathol..

[206] Valera E., Masliah E. (2013). Immunotherapy for neurodegenerative diseases: Focus on alpha-synucleinopathies. Pharm. Ther..

[207] Schneeberger A., Tierney L., Mandler M. (2016). Active immunization therapies for Parkinson’s disease and multiple system atrophy. Mov. Disord..

[208] Fernandez-Valle T., Gabilondo I., Gomez-Esteban J.C. (2019). New therapeutic approaches to target alpha-synuclein in Parkinson’s disease: The role of immunotherapy. Int. Rev. Neurobiol..

[209] Rockenstein E., Ostroff G., Dikengil F., Rus F., Mante M., Florio J., Adame A., Trinh I., Kim C., Overk C. (2018). Combined Active Humoral and Cellular Immunization Approaches for the Treatment of Synucleinopathies. J. Neurosci..

[210] Ugen K.E., Lin X., Bai G., Liang Z., Cai J., Li K., Song S., Cao C., Sanchez-Ramos J. (2015). Evaluation of an alpha synuclein sensitized dendritic cell based vaccine in a transgenic mouse model of Parkinson disease. Hum. Vaccin Immunother..

[211] Zheng Y., Qu J., Xue F., Zheng Y., Yang B., Chang Y., Yang H., Zhang J. (2018). Novel DNA Aptamers for Parkinson’s Disease Treatment Inhibit alpha-Synuclein Aggregation and Facilitate its Degradation. Mol. Therapy Nucleic Acids.

[212] Ren X., Zhao Y., Xue F., Zheng Y., Huang H., Wang W., Chang Y., Yang H., Zhang J. (2019). Exosomal DNA Aptamer Targeting alpha-Synuclein Aggregates Reduced Neuropathological Deficits in a Mouse Parkinson’s Disease Model. Mol. Therapy Nucleic Acids.

[213] Kwon S., Iba M., Masliah E., Kim C. (2019). Targeting Microglial and Neuronal Toll-like Receptor 2 in Synucleinopathies. Exp. Neurobiol..

[214] Kim C., Rockenstein E., Spencer B., Kim H.K., Adame A., Trejo M., Stafa K., Lee H.J., Lee S.J., Masliah E. (2015). Antagonizing Neuronal Toll-like Receptor 2 Prevents Synucleinopathy by Activating Autophagy. Cell Rep..

[215] Kim C., Spencer B., Rockenstein E., Yamakado H., Mante M., Adame A., Fields J.A., Masliah D., Iba M., Lee H.J. (2018). Immunotherapy targeting toll-like receptor 2 alleviates neurodegeneration in models of synucleinopathy by modulating alpha-synuclein transmission and neuroinflammation. Mol. Neurodegener..

[216] Messer A., Butler D.C. (2020). Optimizing intracellular antibodies (intrabodies/nanobodies) to treat neurodegenerative disorders. Neurobiol. Dis..

[217] Yuan B., Sierks M.R. (2009). Intracellular targeting and clearance of oligomeric alpha-synuclein alleviates toxicity in mammalian cells. Neurosci. Lett..

[218] Mahajan S.P., Meksiriporn B., Waraho-Zhmayev D., Weyant K.B., Kocer I., Butler D.C., Messer A., Escobedo F.A., DeLisa M.P. (2018). Computational affinity maturation of camelid single-domain intrabodies against the nonamyloid component of alpha-synuclein. Sci. Rep..

[219] Lynch S.M., Zhou C., Messer A. (2008). An scFv intrabody against the nonamyloid component of alpha-synuclein reduces intracellular aggregation and toxicity. J. Mol. Biol..

[220] El Turk F., De Genst E., Guilliams T., Fauvet B., Hejjaoui M., Di Trani J., Chiki A., Mittermaier A., Vendruscolo M., Lashuel H.A. (2018). Exploring the role of post-translational modifications in regulating alpha-synuclein interactions by studying the effects of phosphorylation on nanobody binding. Protein Sci..

[221] Butler D.C., Joshi S.N., Genst E., Baghel A.S., Dobson C.M., Messer A. (2016). Bifunctional Anti-Non-Amyloid Component alpha-Synuclein Nanobodies Are Protective In Situ. PLoS ONE.

[222] Chatterjee D., Bhatt M., Butler D., De Genst E., Dobson C.M., Messer A., Kordower J.H. (2018). Proteasome-targeted nanobodies alleviate pathology and functional decline in an alpha-synuclein-based Parkinson’s disease model. NPJ Parkinsons Dis..

[223] Atik A., Stewart T., Zhang J. (2016). Alpha-Synuclein as a Biomarker for Parkinson’s Disease. Brain Pathol.

[224] Attar A., Chan W.T., Klarner F.G., Schrader T., Bitan G. (2014). Safety and pharmacological characterization of the molecular tweezer CLR01—A broad-spectrum inhibitor of amyloid proteins’ toxicity. BMC Pharm. Toxicol..

[225] Hadrovic I., Rebmann P., Klarner F.G., Bitan G., Schrader T. (2019). Molecular Lysine Tweezers Counteract Aberrant Protein Aggregation. Front. Chem..

[226] Prabhudesai S., Sinha S., Attar A., Kotagiri A., Fitzmaurice A.G., Lakshmanan R., Ivanova M.I., Loo J.A., Klarner F.G., Schrader T. (2012). A novel “molecular tweezer” inhibitor of alpha-synuclein neurotoxicity in vitro and in vivo. Neurotherapeutics.

[227] Acharya S., Safaie B.M., Wongkongkathep P., Ivanova M.I., Attar A., Klarner F.G., Schrader T., Loo J.A., Bitan G., Lapidus L.J. (2014). Molecular basis for preventing alpha-synuclein aggregation by a molecular tweezer. J. Biol. Chem..

[228] Richter F., Subramaniam S.R., Magen I., Lee P., Hayes J., Attar A., Zhu C., Franich N.R., Bove N., De La Rosa K. (2017). A Molecular Tweezer Ameliorates Motor Deficits in Mice Overexpressing alpha-Synuclein. Neurotherapeutics.

[229] Li H.-T., Lin D.-H., Luo X.-Y., Zhang F., Ji L.-N., Du H.-N., Song G.-Q., Hu J., Zhou J.-W., Hu H.-Y. (2005). Inhibition of alpha-synuclein fibrillization by dopamine analogs via reaction with the amino groups of alpha-synuclein. Implication for dopaminergic neurodegeneration. FEBS J..

[230] Latawiec D., Herrera F., Bek A., Losasso V., Candotti M., Benetti F., Carlino E., Kranjc A., Lazzarino M., Gustincich S. (2010). Modulation of alpha-synuclein aggregation by dopamine analogs. PLoS ONE.

[231] Yedlapudi D., Joshi G.S., Luo D., Todi S.V., Dutta A.K. (2016). Inhibition of alpha-synuclein aggregation by multifunctional dopamine agonists assessed by a novel in vitro assay and an in vivo Drosophila synucleinopathy model. Sci. Rep..

[232] Fernandes L., Moraes N., Sagrillo F.S., Magalhaes A.V., de Moraes M.C., Romao L., Kelly J.W., Foguel D., Grimster N.P., Palhano F.L. (2017). An ortho-Iminoquinone Compound Reacts with Lysine Inhibiting Aggregation while Remodeling Mature Amyloid Fibrils. ACS Chem. Neurosci..

[233] Boettcher J.M., Hartman K.L., Ladror D.T., Qi Z., Woods W.S., George J.M., Rienstra C.M. (2008). Membrane-induced folding of the cAMP-regulated phosphoprotein endosulfine-alpha. Biochemistry.

[234] Woods W.S., Boettcher J.M., Zhou D.H., Kloepper K.D., Hartman K.L., Ladror D.T., Qi Z., Rienstra C.M., George J.M. (2007). Conformation-specific binding of alpha-synuclein to novel protein partners detected by phage display and NMR spectroscopy. J. Biol. Chem..

[235] Ysselstein D., Dehay B., Costantino I.M., McCabe G.P., Frosch M.P., George J.M., Bezard E., Rochet J.C. (2017). Endosulfine-alpha inhibits membrane-induced alpha-synuclein aggregation and protects against alpha-synuclein neurotoxicity. Acta Neuropathol. Commun..

[236] Tatenhorst L., Eckermann K., Dambeck V., Fonseca-Ornelas L., Walle H., Lopes da Fonseca T., Koch J.C., Becker S., Tönges L., Bähr M. (2016). Fasudil attenuates aggregation of α-synuclein in models of Parkinson’s disease. Acta Neuropathol. Commun..

[237] Wrasidlo W., Tsigelny I.F., Price D.L., Dutta G., Rockenstein E., Schwarz T.C., Ledolter K., Bonhaus D., Paulino A., Eleuteri S. (2016). Ade novocompound targeting α-synuclein improves deficits in models of Parkinson’s disease. Brain.

[238] Price D.L., Koike M.A., Khan A., Wrasidlo W., Rockenstein E., Masliah E., Bonhaus D. (2018). The small molecule alpha-synuclein misfolding inhibitor, NPT200-11, produces multiple benefits in an animal model of Parkinson’s disease. Sci. Rep..

[239] Wagner J., Ryazanov S., Leonov A., Levin J., Shi S., Schmidt F., Prix C., Pan-Montojo F., Bertsch U., Mitteregger-Kretzschmar G. (2013). Anle138b: A novel oligomer modulator for disease-modifying therapy of neurodegenerative diseases such as prion and Parkinson’s disease. Acta Neuropathol..

[240] Levin J., Schmidt F., Boehm C., Prix C., Botzel K., Ryazanov S., Leonov A., Griesinger C., Giese A. (2014). The oligomer modulator anle138b inhibits disease progression in a Parkinson mouse model even with treatment started after disease onset. Acta Neuropathol..

[241] Wegrzynowicz M., Bar-On D., Calo’ L., Anichtchik O., Iovino M., Xia J., Ryazanov S., Leonov A., Giese A., Dalley J.W. (2019). Depopulation of dense α-synuclein aggregates is associated with rescue of dopamine neuron dysfunction and death in a new Parkinson’s disease model. Acta Neuropathol..

[242] Saibil H. (2013). Chaperone machines for protein folding, unfolding and disaggregation. Nat. Rev. Mol. Cell Biol..

[243] Uryu K., Richter-Landsberg C., Welch W., Sun E., Goldbaum O., Norris E.H., Pham C.T., Yazawa I., Hilburger K., Micsenyi M. (2006). Convergence of heat shock protein 90 with ubiquitin in filamentous alpha-synuclein inclusions of alpha-synucleinopathies. Am. J. Pathol..

[244] Danzer K.M., Ruf W.P., Putcha P., Joyner D., Hashimoto T., Glabe C., Hyman B.T., McLean P.J. (2011). Heat-shock protein 70 modulates toxic extracellular alpha-synuclein oligomers and rescues trans-synaptic toxicity. FASEB J..

[245] Flower T.R., Chesnokova L.S., Froelich C.A., Dixon C., Witt S.N. (2005). Heat shock prevents alpha-synuclein-induced apoptosis in a yeast model of Parkinson’s disease. J. Mol. Biol..

[246] Auluck P.K., Bonini N.M. (2002). Pharmacological prevention of Parkinson disease in Drosophila. Nat. Med..

[247] Auluck P.K., Chan H.Y., Trojanowski J.Q., Lee V.M., Bonini N.M. (2002). Chaperone suppression of alpha-synuclein toxicity in a Drosophila model for Parkinson’s disease. Science.

[248] Auluck P.K., Meulener M.C., Bonini N.M. (2005). Mechanisms of Suppression of {alpha}-Synuclein Neurotoxicity by Geldanamycin in Drosophila. J. Biol. Chem..

[249] Shen H.-Y., He J.-C., Wang Y., Huang Q.-Y., Chen J.-F. (2005). Geldanamycin Induces Heat Shock Protein 70 and Protects against MPTP-induced Dopaminergic Neurotoxicity in Mice. J. Biol. Chem..

[250] Liu J., Zhang J.P., Shi M., Quinn T., Bradner J., Beyer R., Chen S., Zhang J. (2009). Rab11a and HSP90 Regulate Recycling of Extracellular [alpha]-Synuclein. J. Neurosci..

[251] Putcha P., Danzer K.M., Kranich L.R., Scott A., Silinski M., Mabbett S., Hicks C.D., Veal J.M., Steed P.M., Hyman B.T. (2010). Brain-Permeable Small-Molecule Inhibitors of Hsp90 Prevent α-Synuclein Oligomer Formation and Rescue α-Synuclein-Induced Toxicity. J. Pharmacol. Exp. Ther..

[252] Gendelman H.E., Riedel M., Goldbaum O., Schwarz L., Schmitt S., Richter-Landsberg C. (2010). 17-AAG Induces Cytoplasmic α-Synuclein Aggregate Clearance by Induction of Autophagy. PLoS ONE.

[253] Falsone S.F., Kungl A.J., Rek A., Cappai R., Zangger K. (2009). The molecular chaperone Hsp90 modulates intermediate steps of amyloid assembly of the Parkinson-related protein alpha-synuclein. J. Biol. Chem..

[254] Klucken J., Shin Y., Masliah E., Hyman B.T., McLean P.J. (2004). Hsp70 Reduces alpha-Synuclein Aggregation and Toxicity. J. Biol. Chem..

[255] Yu F., Xu H., Zhuo M., Sun L., Dong A., Liu X. (2005). Impairment of redox state and dopamine level induced by alpha-synuclein aggregation and the prevention effect of hsp70. Biochem. Biophys. Res. Commun..

[256] Outeiro T.F., Kazantsev A. (2008). Drug Targeting of alpha-Synuclein Oligomerization in Synucleinopathies. Perspect Med. Chem..

[257] McLean P.J., Klucken J., Shin Y., Hyman B.T. (2004). Geldanamycin induces Hsp70 and prevents alpha-synuclein aggregation and toxicity in vitro. Biochem. Biophys. Res. Commun..

[258] Kilpatrick K., Novoa J.A., Hancock T., Guerriero C.J., Wipf P., Brodsky J.L., Segatori L. (2013). Chemical induction of Hsp70 reduces alpha-synuclein aggregation in neuroglioma cells. ACS Chem. Biol..

[259] Kalia L.V., Kalia S.K., Chau H., Lozano A.M., Hyman B.T., McLean P.J. (2011). Ubiquitinylation of alpha-synuclein by carboxyl terminus Hsp70-interacting protein (CHIP) is regulated by Bcl-2-associated athanogene 5 (BAG5). PLoS ONE.

[260] Dong Z., Wolfer D.P., Lipp H.P., Bueler H. (2005). Hsp70 gene transfer by adeno-associated virus inhibits MPTP-induced nigrostriatal degeneration in the mouse model of Parkinson disease. Mol. Therapy.

[261] Dedmon M.M., Christodoulou J., Wilson M.R., Dobson C.M. (2005). Heat shock protein 70 inhibits alpha-synuclein fibril formation via preferential binding to prefibrillar species. J. Biol. Chem..

[262] Huang C., Cheng H., Hao S., Zhou H., Zhang X., Gao J., Sun Q.H., Hu H., Wang C.C. (2006). Heat shock protein 70 inhibits alpha-synuclein fibril formation via interactions with diverse intermediates. J. Mol. Biol..

[263] Luk K.C., Mills I.P., Trojanowski J.Q., Lee V.M. (2008). Interactions between Hsp70 and the hydrophobic core of alpha-synuclein inhibit fibril assembly. Biochemistry.

[264] Lo Bianco C., Shorter J., Regulier E., Lashuel H., Iwatsubo T., Lindquist S., Aebischer P. (2008). Hsp104 antagonizes alpha-synuclein aggregation and reduces dopaminergic degeneration in a rat model of Parkinson disease. J. Clin. Investig..

[265] Taguchi Y.V., Gorenberg E.L., Nagy M., Thrasher D., Fenton W.A., Volpicelli-Daley L., Horwich A.L., Chandra S.S. (2019). Hsp110 mitigates α-synuclein pathology in vivo. Proc. Natl. Acad. Sci. USA.

[266] Beyer K., Domingo-Sàbat M., Ariza A. (2009). Molecular pathology of Lewy body diseases. Int. J. Mol. Sci..

[267] Webb J.L., Ravikumar B., Atkins J., Skepper J.N., Rubinsztein D.C. (2003). α-Synuclein Is Degraded by Both Autophagy and the Proteasome. J. Biol. Chem..

[268] Cuervo A.M., Stefanis L., Fredenburg R., Lansbury P.T., Sulzer D. (2004). Impaired Degradation of Mutant α-Synuclein by Chaperone-Mediated Autophagy. Science.

[269] Lee H.-J., Khoshaghideh F., Patel S., Lee S.-J. (2004). Clearance of α-Synuclein Oligomeric Intermediates via the Lysosomal Degradation Pathway. J. Neurosci..

[270] McNaught K.S.P., Belizaire R., Isacson O., Jenner P., Olanow C.W. (2003). Altered Proteasomal Function in Sporadic Parkinson’s Disease. Exp. Neurol..

[271] Martinez-Vicente M., Vila M. (2013). Alpha-synuclein and protein degradation pathways in Parkinson’s disease: A pathological feed-back loop. Exp. Neurol..

[272] Rideout H.J., Larsen K.E., Sulzer D., Stefanis L. (2001). Proteasomal inhibition leads to formation of ubiquitin/α-synuclein-immunoreactive inclusions in PC12 cells. J. Neurochem..

[273] Emmanouilidou E., Stefanis L., Vekrellis K. (2010). Cell-produced α-synuclein oligomers are targeted to, and impair, the 26S proteasome. Neurobiol. Aging.

[274] Snyder H., Mensah K., Theisler C., Lee J., Matouschek A., Wolozin B. (2003). Aggregated and Monomeric α-Synuclein Bind to the S6′ Proteasomal Protein and Inhibit Proteasomal Function. J. Biol. Chem..

[275] Lee F.K.M., Wong A.K.Y., Lee Y.W., Wan O.W., Edwin Chan H.Y., Chung K.K.K. (2009). The role of ubiquitin linkages on α-synuclein induced-toxicity in aDrosophilamodel of Parkinson’s disease. J. Neurochem..

[276] Wang S., He H., Chen L., Zhang W., Zhang X., Chen J. (2015). Protective effects of salidroside in the MPTP/MPP(+)-induced model of Parkinson’s disease through ROS-NO-related mitochondrion pathway. Mol. Neurobiol..

[277] Li T., Feng Y., Yang R., Wu L., Li R., Huang L., Yang Q., Chen J. (2018). Salidroside Promotes the Pathological alpha-Synuclein Clearance Through Ubiquitin-Proteasome System in SH-SY5Y Cells. Front. Pharm..

[278] Leestemaker Y., de Jong A., Witting K.F., Penning R., Schuurman K., Rodenko B., Zaal E.A., van de Kooij B., Laufer S., Heck A.J.R. (2017). Proteasome Activation by Small Molecules. Cell Chem. Biol..

[279] Zhou H., Shao M., Guo B., Li C., Lu Y., Yang X., Li H., Zhu Q., Zhong H., Wang Y. (2019). Tetramethylpyrazine Analogue T-006 Promotes the Clearance of Alpha-synuclein by Enhancing Proteasome Activity in Parkinson’s Disease Models. Neurotherapeutics.

[280] Ebrahimi-Fakhari D., Cantuti-Castelvetri I., Fan Z., Rockenstein E., Masliah E., Hyman B.T., McLean P.J., Unni V.K. (2011). Distinct roles in vivo for the ubiquitin-proteasome system and the autophagy-lysosomal pathway in the degradation of α-synuclein. J. Neurosci. Off. J. Soc. Neurosci..

[281] Mak S.K., McCormack A.L., Manning-Bog A.B., Cuervo A.M., Di Monte D.A. (2010). Lysosomal degradation of alpha-synuclein in vivo. J. Biol. Chem..

[282] Vogiatzi T., Xilouri M., Vekrellis K., Stefanis L. (2008). Wild type alpha-synuclein is degraded by chaperone-mediated autophagy and macroautophagy in neuronal cells. J. Biol. Chem..

[283] Alvarez-Erviti L., Rodriguez-Oroz M.C., Cooper J.M., Caballero C., Ferrer I., Obeso J.A., Schapira A.H. (2010). Chaperone-mediated autophagy markers in Parkinson disease brains. Arch. Neurol..

[284] Murphy K.E., Gysbers A.M., Abbott S.K., Spiro A.S., Furuta A., Cooper A., Garner B., Kabuta T., Halliday G.M. (2015). Lysosomal-associated membrane protein 2 isoforms are differentially affected in early Parkinson’s disease. Mov. Disord..

[285] Martinez-Vicente M., Talloczy Z., Kaushik S., Massey A.C., Mazzulli J., Mosharov E.V., Hodara R., Fredenburg R., Wu D.C., Follenzi A. (2008). Dopamine-modified alpha-synuclein blocks chaperone-mediated autophagy. J. Clin. Investig..

[286] Xilouri M., Vogiatzi T., Vekrellis K., Park D., Stefanis L. (2009). Abberant alpha-synuclein confers toxicity to neurons in part through inhibition of chaperone-mediated autophagy. PLoS ONE.

[287] Yang Q., She H., Gearing M., Colla E., Lee M., Shacka J.J., Mao Z. (2009). Regulation of neuronal survival factor MEF2D by chaperone-mediated autophagy. Science.

[288] Xilouri M., Brekk O.R., Landeck N., Pitychoutis P.M., Papasilekas T., Papadopoulou-Daifoti Z., Kirik D., Stefanis L. (2013). Boosting chaperone-mediated autophagy in vivo mitigates alpha-synuclein-induced neurodegeneration. Brain.

[289] Anguiano J., Garner T.P., Mahalingam M., Das B.C., Gavathiotis E., Cuervo A.M. (2013). Chemical modulation of chaperone-mediated autophagy by retinoic acid derivatives. Nat. Chem. Biol..

[290] Alvarez-Erviti L., Seow Y., Schapira A.H., Rodriguez-Oroz M.C., Obeso J.A., Cooper J.M. (2013). Influence of microRNA deregulation on chaperone-mediated autophagy and alpha-synuclein pathology in Parkinson’s disease. Cell Death Dis..

[291] Su C., Yang X., Lou J. (2016). Geniposide reduces alpha-synuclein by blocking microRNA-21/lysosome-associated membrane protein 2A interaction in Parkinson disease models. Brain Res..

[292] Khoo S.K., Neuman L.A., Forsgren L., Petillo D., Brundin P. (2013). Could miRNA expression changes be a reliable clinical biomarker for Parkinson’s disease?. Neurodegener. Dis. Manag..

[293] Dehay B., Bove J., Rodriguez-Muela N., Perier C., Recasens A., Boya P., Vila M. (2010). Pathogenic lysosomal depletion in Parkinson’s disease. J. Neurosci..

[294] Chu Y., Dodiya H., Aebischer P., Olanow C.W., Kordower J.H. (2009). Alterations in lysosomal and proteasomal markers in Parkinson’s disease: Relationship to alpha-synuclein inclusions. Neurobiol. Dis..

[295] Dehay B., Ramirez A., Martinez-Vicente M., Perier C., Canron M.H., Doudnikoff E., Vital A., Vila M., Klein C., Bezard E. (2012). Loss of P-type ATPase ATP13A2/PARK9 function induces general lysosomal deficiency and leads to Parkinson disease neurodegeneration. Proc. Natl. Acad. Sci. USA.

[296] Murphy K.E., Cottle L., Gysbers A.M., Cooper A.A., Halliday G.M. (2013). ATP13A2 (PARK9) protein levels are reduced in brain tissue of cases with Lewy bodies. Acta Neuropathol. Commun..

[297] Chiasserini D., Paciotti S., Eusebi P., Persichetti E., Tasegian A., Kurzawa-Akanbi M., Chinnery P.F., Morris C.M., Calabresi P., Parnetti L. (2015). Selective loss of glucocerebrosidase activity in sporadic Parkinson’s disease and dementia with Lewy bodies. Mol. Neurodegener..

[298] Moors T.E., Paciotti S., Ingrassia A., Quadri M., Breedveld G., Tasegian A., Chiasserini D., Eusebi P., Duran-Pacheco G., Kremer T. (2019). Characterization of Brain Lysosomal Activities in GBA-Related and Sporadic Parkinson’s Disease and Dementia with Lewy Bodies. Mol. Neurobiol..

[299] Gündner A.L., Duran-Pacheco G., Zimmermann S., Ruf I., Moors T., Baumann K., Jagasia R., van de Berg W.D.J., Kremer T. (2019). Path mediation analysis reveals GBA impacts Lewy body disease status by increasing α-synuclein levels. Neurobiol. Dis..

[300] Sato S., Uchihara T., Fukuda T., Noda S., Kondo H., Saiki S., Komatsu M., Uchiyama Y., Tanaka K., Hattori N. (2018). Loss of autophagy in dopaminergic neurons causes Lewy pathology and motor dysfunction in aged mice. Sci. Rep..

[301] Winslow A.R., Chen C.W., Corrochano S., Acevedo-Arozena A., Gordon D.E., Peden A.A., Lichtenberg M., Menzies F.M., Ravikumar B., Imarisio S. (2010). alpha-Synuclein impairs macroautophagy: Implications for Parkinson’s disease. J. Cell Biol..

[302] Song J.X., Lu J.H., Liu L.F., Chen L.L., Durairajan S.S., Yue Z., Zhang H.Q., Li M. (2014). HMGB1 is involved in autophagy inhibition caused by SNCA/alpha-synuclein overexpression: A process modulated by the natural autophagy inducer corynoxine B. Autophagy.

[303] Stefanovic A.N., Stockl M.T., Claessens M.M., Subramaniam V. (2014). alpha-Synuclein oligomers distinctively permeabilize complex model membranes. FEBS J..

[304] Crews L., Spencer B., Desplats P., Patrick C., Paulino A., Rockenstein E., Hansen L., Adame A., Galasko D., Masliah E. (2010). Selective molecular alterations in the autophagy pathway in patients with Lewy body disease and in models of alpha-synucleinopathy. PLoS ONE.

[305] Bai X., Wey M.C.-Y., Fernandez E., Hart M.J., Gelfond J., Bokov A.F., Rani S., Strong R. (2015). Rapamycin improves motor function, reduces 4-hydroxynonenal adducted protein in brain, and attenuates synaptic injury in a mouse model of synucleinopathy. Pathobiol. Aging Age Relat. Dis..

[306] Decressac M., Mattsson B., Weikop P., Lundblad M., Jakobsson J., Bjorklund A. (2013). TFEB-mediated autophagy rescues midbrain dopamine neurons from alpha-synuclein toxicity. Proc. Natl. Acad. Sci. USA.

[307] Bove J., Martinez-Vicente M., Vila M. (2011). Fighting neurodegeneration with rapamycin: Mechanistic insights. Nat. Rev. Neurosci..

[308] Guo Y.J., Dong S.Y., Cui X.X., Feng Y., Liu T., Yin M., Kuo S.H., Tan E.K., Zhao W.J., Wu Y.C. (2016). Resveratrol alleviates MPTP-induced motor impairments and pathological changes by autophagic degradation of alpha-synuclein via SIRT1-deacetylated LC3. Mol. Nutr Food Res..

[309] Wang Z.H., Zhang J.L., Duan Y.L., Zhang Q.S., Li G.F., Zheng D.L. (2015). MicroRNA-214 participates in the neuroprotective effect of Resveratrol via inhibiting alpha-synuclein expression in MPTP-induced Parkinson’s disease mouse. Biomed. Pharm..

[310] Wu F., Xu H.D., Guan J.J., Hou Y.S., Gu J.H., Zhen X.C., Qin Z.H. (2015). Rotenone impairs autophagic flux and lysosomal functions in Parkinson’s disease. Neuroscience.

[311] Sardiello M., Palmieri M., di Ronza A., Medina D.L., Valenza M., Gennarino V.A., Di Malta C., Donaudy F., Embrione V., Polishchuk R.S. (2009). A Gene Network Regulating Lysosomal Biogenesis and Function. Science.

[312] Settembre C., Ballabio A. (2011). TFEB regulates autophagy: An integrated coordination of cellular degradation and recycling processes. Autophagy.

[313] Settembre C., Di Malta C., Polito V.A., Garcia Arencibia M., Vetrini F., Erdin S., Erdin S.U., Huynh T., Medina D., Colella P. (2011). TFEB links autophagy to lysosomal biogenesis. Science.

[314] Settembre C., Zoncu R., Medina D.L., Vetrini F., Erdin S., Erdin S., Huynh T., Ferron M., Karsenty G., Vellard M.C. (2012). A lysosome-to-nucleus signalling mechanism senses and regulates the lysosome via mTOR and TFEB. EMBO J..

[315] Martini-Stoica H., Xu Y., Ballabio A., Zheng H. (2016). The Autophagy-Lysosomal Pathway in Neurodegeneration: A TFEB Perspective. Trends Neurosci..

[316] Arotcarena M.-L., Bourdenx M., Dutheil N., Thiolat M.-L., Doudnikoff E., Dovero S., Ballabio A., Fernagut P.-O., Meissner W.G., Bezard E. (2019). Transcription factor EB overexpression prevents neurodegeneration in experimental synucleinopathies. JCI Insight.

[317] Torra A., Parent A., Cuadros T., Rodriguez-Galvan B., Ruiz-Bronchal E., Ballabio A., Bortolozzi A., Vila M., Bove J. (2018). Overexpression of TFEB Drives a Pleiotropic Neurotrophic Effect and Prevents Parkinson’s Disease-Related Neurodegeneration. Mol. Therapy.

[318] Kilpatrick K., Zeng Y., Hancock T., Segatori L. (2015). Genetic and chemical activation of TFEB mediates clearance of aggregated alpha-synuclein. PLoS ONE.

[319] Song W., Wang F., Lotfi P., Sardiello M., Segatori L. (2014). 2-Hydroxypropyl-beta-cyclodextrin promotes transcription factor EB-mediated activation of autophagy: Implications for therapy. J. Biol. Chem..

[320] Tan S., Yu C.Y., Sim Z.W., Low Z.S., Lee B., See F., Min N., Gautam A., Chu J.J.H., Ng K.W. (2019). Pomegranate activates TFEB to promote autophagy-lysosomal fitness and mitophagy. Sci. Rep..

[321] Lan D.M., Liu F.T., Zhao J., Chen Y., Wu J.J., Ding Z.T., Yue Z.Y., Ren H.M., Jiang Y.P., Wang J. (2012). Effect of trehalose on PC12 cells overexpressing wild-type or A53T mutant alpha-synuclein. Neurochem. Res..

[322] Sarkar S., Davies J.E., Huang Z., Tunnacliffe A., Rubinsztein D.C. (2007). Trehalose, a novel mTOR-independent autophagy enhancer, accelerates the clearance of mutant huntingtin and alpha-synuclein. J. Biol. Chem..

[323] Hoffmann A.C., Minakaki G., Menges S., Salvi R., Savitskiy S., Kazman A., Vicente Miranda H., Mielenz D., Klucken J., Winkler J. (2019). Extracellular aggregated alpha synuclein primarily triggers lysosomal dysfunction in neural cells prevented by trehalose. Sci. Rep..

[324] Tanji K., Miki Y., Maruyama A., Mimura J., Matsumiya T., Mori F., Imaizumi T., Itoh K., Wakabayashi K. (2015). Trehalose intake induces chaperone molecules along with autophagy in a mouse model of Lewy body disease. Biochem. Biophys. Res. Commun..

[325] DeBosch B.J., Heitmeier M.R., Mayer A.L., Higgins C.B., Crowley J.R., Kraft T.E., Chi M., Newberry E.P., Chen Z., Finck B.N. (2016). Trehalose inhibits solute carrier 2A (SLC2A) proteins to induce autophagy and prevent hepatic steatosis. Sci. Signal..

[326] Howson P.A., Johnston T.H., Ravenscroft P., Hill M.P., Su J., Brotchie J.M., Koprich J.B. (2019). Beneficial Effects of Trehalose on Striatal Dopaminergic Deficits in Rodent and Primate Models of Synucleinopathy in Parkinson’s Disease. J. Pharm. Exp. Ther..

[327] Hebron M.L., Lonskaya I., Moussa C.E. (2013). Nilotinib reverses loss of dopamine neurons and improves motor behavior via autophagic degradation of alpha-synuclein in Parkinson’s disease models. Hum. Mol. Genet..

[328] Mahul-Mellier A.L., Fauvet B., Gysbers A., Dikiy I., Oueslati A., Georgeon S., Lamontanara A.J., Bisquertt A., Eliezer D., Masliah E. (2014). c-Abl phosphorylates alpha-synuclein and regulates its degradation: Implication for alpha-synuclein clearance and contribution to the pathogenesis of Parkinson’s disease. Hum. Mol. Genet..

[329] Pagan F., Hebron M., Valadez E.H., Torres-Yaghi Y., Huang X., Mills R.R., Wilmarth B.M., Howard H., Dunn C., Carlson A. (2016). Nilotinib Effects in Parkinson’s disease and Dementia with Lewy bodies. J. Parkinson’s Dis..

[330] Pagan F.L., Hebron M.L., Wilmarth B., Torres-Yaghi Y., Lawler A., Mundel E.E., Yusuf N., Starr N.J., Arellano J., Howard H.H. (2019). Pharmacokinetics and pharmacodynamics of a single dose Nilotinib in individuals with Parkinson’s disease. Pharmacol. Res. Perspect..

[331] Spencer B., Potkar R., Trejo M., Rockenstein E., Patrick C., Gindi R., Adame A., Wyss-Coray T., Masliah E. (2009). Beclin 1 gene transfer activates autophagy and ameliorates the neurodegenerative pathology in alpha-synuclein models of Parkinson’s and Lewy body diseases. J. Neurosci..

[332] Wang K., Huang J., Xie W., Huang L., Zhong C., Chen Z. (2016). Beclin1 and HMGB1 ameliorate the alpha-synuclein-mediated autophagy inhibition in PC12 cells. Diagn. Pathol..

[333] Lu J.H., Tan J.Q., Durairajan S.S., Liu L.F., Zhang Z.H., Ma L., Shen H.M., Chan H.Y., Li M. (2012). Isorhynchophylline, a natural alkaloid, promotes the degradation of alpha-synuclein in neuronal cells via inducing autophagy. Autophagy.

[334] Higdon J.V., Frei B. (2006). Coffee and Health: A Review of Recent Human Research. Crit. Rev. Food Sci. Nutr..

[335] Luan Y., Ren X., Zheng W., Zeng Z., Guo Y., Hou Z., Guo W., Chen X., Li F., Chen J.F. (2018). Chronic Caffeine Treatment Protects Against alpha-Synucleinopathy by Reestablishing Autophagy Activity in the Mouse Striatum. Front. Neurosci..

[336] Ambrosi G., Ghezzi C., Zangaglia R., Levandis G., Pacchetti C., Blandini F. (2015). Ambroxol-induced rescue of defective glucocerebrosidase is associated with increased LIMP-2 and saposin C levels in GBA1 mutant Parkinson’s disease cells. Neurobiol. Dis..

[337] McNeill A., Magalhaes J., Shen C., Chau K.-Y., Hughes D., Mehta A., Foltynie T., Cooper J.M., Abramov A.Y., Gegg M. (2014). Ambroxol improves lysosomal biochemistry in glucocerebrosidase mutation-linked Parkinson disease cells. Brain.

[338] Sardi S.P., Clarke J., Kinnecom C., Tamsett T.J., Li L., Stanek L.M., Passini M.A., Grabowski G.A., Schlossmacher M.G., Sidman R.L. (2011). CNS expression of glucocerebrosidase corrects alpha-synuclein pathology and memory in a mouse model of Gaucher-related synucleinopathy. Proc. Natl. Acad. Sci. USA.

[339] Gegg M.E., Burke D., Heales S.J., Cooper J.M., Hardy J., Wood N.W., Schapira A.H. (2012). Glucocerebrosidase deficiency in substantia nigra of parkinson disease brains. Ann. Neurol..

[340] Murphy K.E., Gysbers A.M., Abbott S.K., Tayebi N., Kim W.S., Sidransky E., Cooper A., Garner B., Halliday G.M. (2014). Reduced glucocerebrosidase is associated with increased alpha-synuclein in sporadic Parkinson’s disease. Brain.

[341] Mazzulli J.R., Xu Y.H., Sun Y., Knight A.L., McLean P.J., Caldwell G.A., Sidransky E., Grabowski G.A., Krainc D. (2011). Gaucher disease glucocerebrosidase and alpha-synuclein form a bidirectional pathogenic loop in synucleinopathies. Cell.

[342] Zunke F., Moise A.C., Belur N.R., Gelyana E., Stojkovska I., Dzaferbegovic H., Toker N.J., Jeon S., Fredriksen K., Mazzulli J.R. (2018). Reversible Conformational Conversion of alpha-Synuclein into Toxic Assemblies by Glucosylceramide. Neuron.

[343] Henderson M.X., Sedor S., McGeary I., Cornblath E.J., Peng C., Riddle D.M., Li H.L., Zhang B., Brown H.J., Olufemi M.F. (2019). Glucocerebrosidase Activity Modulates Neuronal Susceptibility to Pathological alpha-Synuclein Insult. Neuron.

[344] Mazzulli J.R., Zunke F., Isacson O., Studer L., Krainc D. (2016). alpha-Synuclein-induced lysosomal dysfunction occurs through disruptions in protein trafficking in human midbrain synucleinopathy models. Proc. Natl. Acad. Sci. USA.

[345] Do J., McKinney C., Sharma P., Sidransky E. (2019). Glucocerebrosidase and its relevance to Parkinson disease. Mol. Neurodegener..

[346] Migdalska-Richards A., Daly L., Bezard E., Schapira A.H. (2016). Ambroxol effects in glucocerebrosidase and alpha-synuclein transgenic mice. Ann. Neurol..

[347] Narita A., Shirai K., Itamura S., Matsuda A., Ishihara A., Matsushita K., Fukuda C., Kubota N., Takayama R., Shigematsu H. (2016). Ambroxol chaperone therapy for neuronopathic Gaucher disease: A pilot study. Ann. Clin. Transl. Neurol..

[348] Silveira C.R.A., MacKinley J., Coleman K., Li Z., Finger E., Bartha R., Morrow S.A., Wells J., Borrie M., Tirona R.G. (2019). Ambroxol as a novel disease-modifying treatment for Parkinson’s disease dementia: Protocol for a single-centre, randomized, double-blind, placebo-controlled trial. BMC Neurol..

[349] Mullin S., Smith L., Lee K., D’Souza G., Woodgate P., Elflein J., Hällqvist J., Toffoli M., Streeter A., Hosking J. (2020). Ambroxol for the Treatment of Patients With Parkinson Disease With and Without Glucocerebrosidase Gene Mutations: A Nonrandomized, Noncontrolled Trial. JAMA Neurol..

[350] Steet R.A., Chung S., Wustman B., Powe A., Do H., Kornfeld S.A. (2006). The iminosugar isofagomine increases the activity of N370S mutant acid β-glucosidase in Gaucher fibroblasts by several mechanisms. Proc. Natl. Acad. Sci. USA.

[351] Khanna R., Benjamin E.R., Pellegrino L., Schilling A., Rigat B.A., Soska R., Nafar H., Ranes B.E., Feng J., Lun Y. (2010). The pharmacological chaperone isofagomine increases the activity of the Gaucher disease L444P mutant form of beta-glucosidase. FEBS J..

[352] Sun Y., Liou B., Xu Y.H., Quinn B., Zhang W., Hamler R., Setchell K.D., Grabowski G.A. (2012). Ex vivo and in vivo effects of isofagomine on acid beta-glucosidase variants and substrate levels in Gaucher disease. J. Biol. Chem..

[353] Yang C., Rahimpour S., Lu J., Pacak K., Ikejiri B., Brady R.O., Zhuang Z. (2013). Histone deacetylase inhibitors increase glucocerebrosidase activity in Gaucher disease by modulation of molecular chaperones. Proc. Natl. Acad. Sci. USA.

[354] Richter F., Fleming S.M., Watson M., Lemesre V., Pellegrino L., Ranes B., Zhu C., Mortazavi F., Mulligan C.K., Sioshansi P.C. (2014). A GCase chaperone improves motor function in a mouse model of synucleinopathy. Neurotherapeutics.

[355] Aflaki E., Borger D.K., Moaven N., Stubblefield B.K., Rogers S.A., Patnaik S., Schoenen F.J., Westbroek W., Zheng W., Sullivan P. (2016). A New Glucocerebrosidase Chaperone Reduces alpha-Synuclein and Glycolipid Levels in iPSC-Derived Dopaminergic Neurons from Patients with Gaucher Disease and Parkinsonism. J. Neurosci..

[356] Mazzulli J.R., Zunke F., Tsunemi T., Toker N.J., Jeon S., Burbulla L.F., Patnaik S., Sidransky E., Marugan J.J., Sue C.M. (2016). Activation of beta-Glucocerebrosidase Reduces Pathological alpha-Synuclein and Restores Lysosomal Function in Parkinson’s Patient Midbrain Neurons. J. Neurosci..

[357] Burbulla L.F., Jeon S., Zheng J., Song P., Silverman R.B., Krainc D. (2019). A modulator of wild-type glucocerebrosidase improves pathogenic phenotypes in dopaminergic neuronal models of Parkinson’s disease. Sci. Transl. Med..

[358] Qiao L., Hamamichi S., Caldwell K.A., Caldwell G.A., Yacoubian T.A., Wilson S., Xie Z.L., Speake L.D., Parks R., Crabtree D. (2008). Lysosomal enzyme cathepsin D protects against alpha-synuclein aggregation and toxicity. Mol. Brain.

[359] Pupyshev A.B., Tikhonova M.A., Akopyan A.A., Tenditnik M.V., Dubrovina N.I., Korolenko T.A. (2019). Therapeutic activation of autophagy by combined treatment with rapamycin and trehalose in a mouse MPTP-induced model of Parkinson’s disease. Pharm. Biochem. Behav..

[360] Singh P.K., Kotia V., Ghosh D., Mohite G.M., Kumar A., Maji S.K. (2013). Curcumin modulates alpha-synuclein aggregation and toxicity. ACS Chem. Neurosci..

[361] Ono K., Yamada M. (2006). Antioxidant compounds have potent anti-fibrillogenic and fibril-destabilizing effects for alpha-synuclein fibrils in vitro. J. Neurochem..

[362] Shoval H., Weiner L., Gazit E., Levy M., Pinchuk I., Lichtenberg D. (2008). Polyphenol-induced dissociation of various amyloid fibrils results in a methionine-independent formation of ROS. Biochim. Biophys. Acta (BBA) Proteins Proteom..

[363] Bhatia N.K., Srivastava A., Katyal N., Jain N., Khan M.A., Kundu B., Deep S. (2015). Curcumin binds to the pre-fibrillar aggregates of Cu/Zn superoxide dismutase (SOD1) and alters its amyloidogenic pathway resulting in reduced cytotoxicity. Biochim. Biophys. Acta.

[364] Ahmad B., Lapidus L.J. (2012). Curcumin Prevents Aggregation in α-Synuclein by Increasing Reconfiguration Rate. J. Biol. Chem..

[365] Gautam S., Karmakar S., Batra R., Sharma P., Pradhan P., Singh J., Kundu B., Chowdhury P.K. (2017). Polyphenols in combination with β-cyclodextrin can inhibit and disaggregate α-synuclein amyloids under cell mimicking conditions: A promising therapeutic alternative. Biochim. Biophys. Acta (BBA) Proteins Proteom..

[366] Tavassoly O., Kakish J., Nokhrin S., Dmitriev O., Lee J.S. (2014). The use of nanopore analysis for discovering drugs which bind to α-synuclein for treatment of Parkinson’s disease. Eur. J. Med. Chem..

[367] Wang M.S., Boddapati S., Emadi S., Sierks M.R. (2010). Curcumin reduces α-synuclein induced cytotoxicity in Parkinson’s disease cell model. BMC Neurosci..

[368] Liu Z., Yu Y., Li X., Ross C.A., Smith W.W. (2011). Curcumin protects against A53T alpha-synuclein-induced toxicity in a PC12 inducible cell model for Parkinsonism. Pharmacol. Res..

[369] Spinelli K.J., Osterberg V.R., Meshul C.K., Soumyanath A., Unni V.K. (2015). Curcumin Treatment Improves Motor Behavior in alpha-Synuclein Transgenic Mice. PLoS ONE.

[370] Sharma N., Nehru B. (2017). Curcumin affords neuroprotection and inhibits α-synuclein aggregation in lipopolysaccharide-induced Parkinson’s disease model. Inflammopharmacology.

[371] Ahsan N., Mishra S., Jain M.K., Surolia A., Gupta S. (2015). Curcumin Pyrazole and its derivative (N-(3-Nitrophenylpyrazole) Curcumin inhibit aggregation, disrupt fibrils and modulate toxicity of Wild type and Mutant α-Synuclein. Sci. Rep..

[372] Marchiani A., Mammi S., Siligardi G., Hussain R., Tessari I., Bubacco L., Delogu G., Fabbri D., Dettori M.A., Sanna D. (2013). Small molecules interacting with α-synuclein: Antiaggregating and cytoprotective properties. Amino Acids.

[373] Gadad B.S., Subramanya P.K., Pullabhatla S., Shantharam I.S., Rao K.S. (2012). Curcumin-glucoside, a novel synthetic derivative of curcumin, inhibits alpha-synuclein oligomer formation: Relevance to Parkinson’s disease. Curr. Pharm Des..

[374] Taebnia N., Morshedi D., Yaghmaei S., Aliakbari F., Rahimi F., Arpanaei A. (2016). Curcumin-Loaded Amine-Functionalized Mesoporous Silica Nanoparticles Inhibit α-Synuclein Fibrillation and Reduce Its Cytotoxicity-Associated Effects. Langmuir.

[375] Kundu P., Das M., Tripathy K., Sahoo S.K. (2016). Delivery of Dual Drug Loaded Lipid Based Nanoparticles across the Blood-Brain Barrier Impart Enhanced Neuroprotection in a Rotenone Induced Mouse Model of Parkinson’s Disease. ACS Chem. Neurosci..

[376] Bollimpelli V.S., Kumar P., Kumari S., Kondapi A.K. (2016). Neuroprotective effect of curcumin-loaded lactoferrin nano particles against rotenone induced neurotoxicity. Neurochem. Int..

[377] Gautam S., Karmakar S., Bose A., Chowdhury P.K. (2014). beta-cyclodextrin and curcumin, a potent cocktail for disaggregating and/or inhibiting amyloids: A case study with alpha-synuclein. Biochemistry.

[378] Zhang N., Yan F., Liang X., Wu M., Shen Y., Chen M., Xu Y., Zou G., Jiang P., Tang C. (2018). Localized delivery of curcumin into brain with polysorbate 80-modified cerasomes by ultrasound-targeted microbubble destruction for improved Parkinson’s disease therapy. Theranostics.

[379] Kosuru R.Y., Roy A., Das S.K., Bera S. (2018). Gallic Acid and Gallates in Human Health and Disease: Do Mitochondria Hold the Key to Success?. Mol. Nutr. Food Res..

[380] Ardah M.T., Paleologou K.E., Lv G., Abul Khair S.B., Kazim A.S., Minhas S.T., Al-Tel T.H., Al-Hayani A.A., Haque M.E., Eliezer D. (2014). Structure activity relationship of phenolic acid inhibitors of α-synuclein fibril formation and toxicity. Front. Aging Neurosci..

[381] Liu Y., Carver J.A., Calabrese A.N., Pukala T.L. (2014). Gallic acid interacts with α-synuclein to prevent the structural collapse necessary for its aggregation. Biochim. Biophys. Acta (BBA) Proteins Proteom..

[382] Garcia-Moreno J.C., Porta de la Riva M., Martínez-Lara E., Siles E., Cañuelo A. (2019). Tyrosol, a simple phenol from EVOO, targets multiple pathogenic mechanisms of neurodegeneration in a C. elegans model of Parkinson’s disease. Neurobiol. Aging.

[383] Gasiorowski K., Lamer-Zarawska E., Leszek J., Parvathaneni K., Yendluri B.B., Blach-Olszewska Z., Aliev G. (2011). Flavones from root of Scutellaria baicalensis Georgi: Drugs of the future in neurodegeneration?. CNS Neurol. Disord. Drug Targets.

[384] Meng X., Munishkina L.A., Fink A.L., Uversky V.N. (2009). Molecular mechanisms underlying the flavonoid-induced inhibition of alpha-synuclein fibrillation. Biochemistry.

[385] Caruana M., Högen T., Levin J., Hillmer A., Giese A., Vassallo N. (2011). Inhibition and disaggregation of α-synuclein oligomers by natural polyphenolic compounds. FEBS Lett..

[386] Caruana M., Neuner J., Högen T., Schmidt F., Kamp F., Scerri C., Giese A., Vassallo N. (2012). Polyphenolic compounds are novel protective agents against lipid membrane damage by α-synuclein aggregates in vitro. Biochim. Biophys. Acta (BBA) Biomembr..

[387] Zhu Q., Zhuang X., Lu J. (2019). Neuroprotective effects of baicalein in animal models of Parkinson’s disease: A systematic review of experimental studies. Phytomedicine.

[388] Lu J.H., Ardah M.T., Durairajan S.S., Liu L.F., Xie L.X., Fong W.F., Hasan M.Y., Huang J.D., El-Agnaf O.M., Li M. (2011). Baicalein inhibits formation of alpha-synuclein oligomers within living cells and prevents Abeta peptide fibrillation and oligomerisation. Chembiochem.

[389] Hong D.P., Fink A.L., Uversky V.N. (2008). Structural characteristics of alpha-synuclein oligomers stabilized by the flavonoid baicalein. J. Mol. Biol..

[390] Li X., Zhang G., Nie Q., Wu T., Jiao L., Zheng M., Wan X., Li Y., Wu S., Jiang B. (2017). Baicalein blocks alpha-synuclein secretion from SN4741 cells and facilitates alpha-synuclein polymerization to big complex. Neurosci. Lett..

[391] Kostka M., Högen T., Danzer K.M., Levin J., Habeck M., Wirth A., Wagner R., Glabe C.G., Finger S., Heinzelmann U. (2008). Single Particle Characterization of Iron-induced Pore-forming α-Synuclein Oligomers. J. Biol. Chem..

[392] Hu Q., Uversky V.N., Huang M., Kang H., Xu F., Liu X., Lian L., Liang Q., Jiang H., Liu A. (2016). Baicalein inhibits alpha-synuclein oligomer formation and prevents progression of alpha-synuclein accumulation in a rotenone mouse model of Parkinson’s disease. Biochim. Biophys. Acta.

[393] Hung K.C., Huang H.J., Wang Y.T., Lin A.M. (2016). Baicalein attenuates alpha-synuclein aggregation, inflammasome activation and autophagy in the MPP(+)-treated nigrostriatal dopaminergic system in vivo. J. Ethnopharmacol..

[394] Morshedi D., Aliakbari F., Tayaranian-Marvian A., Fassihi A., Pan-Montojo F., Perez-Sanchez H. (2015). Cuminaldehyde as the Major Component of Cuminum cyminum, a Natural Aldehyde with Inhibitory Effect on Alpha-Synuclein Fibrillation and Cytotoxicity. J. Food Sci..

[395] Morshedi D., Nasouti M. (2016). Essential Oils May Lead alpha-Synuclein towards Toxic Fibrils Formation. Parkinsons Dis..

[396] Sneideris T., Baranauskiene L., Cannon J.G., Rutkiene R., Meskys R., Smirnovas V. (2015). Looking for a generic inhibitor of amyloid-like fibril formation among flavone derivatives. PeerJ.

[397] Pogacnik L., Pirc K., Palmela I., Skrt M., Kim K.S., Brites D., Brito M.A., Ulrih N.P., Silva R.F. (2016). Potential for brain accessibility and analysis of stability of selected flavonoids in relation to neuroprotection in vitro. Brain Res..

[398] Xu Y., Zhang Y., Quan Z., Wong W., Guo J., Zhang R., Yang Q., Dai R., McGeer P.L., Qing H. (2016). Epigallocatechin Gallate (EGCG) Inhibits Alpha-Synuclein Aggregation: A Potential Agent for Parkinson’s Disease. Neurochem. Res..

[399] Ehrnhoefer D.E., Bieschke J., Boeddrich A., Herbst M., Masino L., Lurz R., Engemann S., Pastore A., Wanker E.E. (2008). EGCG redirects amyloidogenic polypeptides into unstructured, off-pathway oligomers. Nat. Struct Mol. Biol..

[400] Bieschke J., Russ J., Friedrich R.P., Ehrnhoefer D.E., Wobst H., Neugebauer K., Wanker E.E. (2010). EGCG remodels mature alpha-synuclein and amyloid-beta fibrils and reduces cellular toxicity. Proc. Natl. Acad. Sci. USA.

[401] Trinh H.T., Joh E.H., Kwak H.Y., Baek N.I., Kim D.H. (2010). Anti-pruritic effect of baicalin and its metabolites, baicalein and oroxylin A, in mice. Acta Pharm. Sin..

[402] Liu X., Zhou S., Shi D., Bai Q., Liu H., Yao X. (2018). Influence of EGCG on alpha-synuclein (alphaS) aggregation and identification of their possible binding mode: A computational study using molecular dynamics simulation. Chem. Biol. Drug Des..

[403] Lorenzen N., Nielsen S.B., Yoshimura Y., Vad B.S., Andersen C.B., Betzer C., Kaspersen J.D., Christiansen G., Pedersen J.S., Jensen P.H. (2014). How epigallocatechin gallate can inhibit alpha-synuclein oligomer toxicity in vitro. J. Biol. Chem..

[404] Yang J.E., Rhoo K.Y., Lee S., Lee J.T., Park J.H., Bhak G., Paik S.R. (2017). EGCG-mediated Protection of the Membrane Disruption and Cytotoxicity Caused by the ‘Active Oligomer’ of alpha-Synuclein. Sci. Rep..

[405] Weinreb O., Mandel S., Youdim M.B.H., Amit T. (2013). Targeting dysregulation of brain iron homeostasis in Parkinson’s disease by iron chelators. Free Radic. Biol. Med..

[406] Palhano F.L., Lee J., Grimster N.P., Kelly J.W. (2013). Toward the molecular mechanism(s) by which EGCG treatment remodels mature amyloid fibrils. J. Am. Chem. Soc..

[407] Li Y., Chen Z., Lu Z., Yang Q., Liu L., Jiang Z., Zhang L., Zhang X., Qing H. (2018). “Cell-addictive” dual-target traceable nanodrug for Parkinson’s disease treatment via flotillins pathway. Theranostics.

[408] Grelle G., Otto A., Lorenz M., Frank R.F., Wanker E.E., Bieschke J. (2011). Black tea theaflavins inhibit formation of toxic amyloid-beta and alpha-synuclein fibrils. Biochemistry.

[409] Noack H., Kube U., Augustin W. (1994). Relations between tocopherol depletion and coenzyme Q during lipid peroxidation in rat liver mitochondria. Free Radic. Res..

[410] Forsmark-Andree P., Lee C.P., Dallner G., Ernster L. (1997). Lipid peroxidation and changes in the ubiquinone content and the respiratory chain enzymes of submitochondrial particles. Free Radic. Biol. Med..

[411] Schulz J.B., Henshaw D.R., Matthews R.T., Beal M.F. (1995). Coenzyme Q10 and nicotinamide and a free radical spin trap protect against MPTP neurotoxicity. Exp. Neurol..

[412] Cleren C., Yang L., Lorenzo B., Calingasan N.Y., Schomer A., Sireci A., Wille E.J., Beal M.F. (2008). Therapeutic effects of coenzyme Q10 (CoQ10) and reduced CoQ10 in the MPTP model of Parkinsonism. J. Neurochem..

[413] Yang L., Calingasan N.Y., Wille E.J., Cormier K., Smith K., Ferrante R.J., Beal M.F. (2009). Combination therapy with coenzyme Q10 and creatine produces additive neuroprotective effects in models of Parkinson’s and Huntington’s diseases. J. Neurochem..

[414] Shults C.W., Oakes D., Kieburtz K., Beal M.F., Haas R., Plumb S., Juncos J.L., Nutt J., Shoulson I., Carter J. (2002). Effects of coenzyme Q10 in early Parkinson disease: Evidence of slowing of the functional decline. Arch. Neurol..

[415] Esteves A.R., Arduino D.M., Swerdlow R.H., Oliveira C.R., Cardoso S.M. (2009). Oxidative stress involvement in alpha-synuclein oligomerization in Parkinson’s disease cybrids. Antioxid. Redox. Signal..

[416] Mohanan P., Subramaniyam S., Mathiyalagan R., Yang D.C. (2018). Molecular signaling of ginsenosides Rb1, Rg1, and Rg3 and their mode of actions. J. Ginseng. Res..

[417] Ardah M.T., Paleologou K.E., Lv G., Menon S.A., Abul Khair S.B., Lu J.-H., Safieh-Garabedian B., Al-Hayani A.A., Eliezer D., Li M. (2015). Ginsenoside Rb1 inhibits fibrillation and toxicity of alpha-synuclein and disaggregates preformed fibrils. Neurobiol. Dis..

[418] Heng Y., Zhang Q.S., Mu Z., Hu J.F., Yuan Y.H., Chen N.H. (2016). Ginsenoside Rg1 attenuates motor impairment and neuroinflammation in the MPTP-probenecid-induced parkinsonism mouse model by targeting alpha-synuclein abnormalities in the substantia nigra. Toxicol. Lett..

[419] Van Kampen J.M., Baranowski D.B., Shaw C.A., Kay D.G. (2014). Panax ginseng is neuroprotective in a novel progressive model of Parkinson’s disease. Exp. Gerontol..

[420] Tatemoto K., Hosoya M., Habata Y., Fujii R., Kakegawa T., Zou M.X., Kawamata Y., Fukusumi S., Hinuma S., Kitada C. (1998). Isolation and characterization of a novel endogenous peptide ligand for the human APJ receptor. Biochem. Biophys. Res. Commun..

[421] Zhu J., Dou S., Jiang Y., Bai B., Chen J., Wang C., Cheng B. (2019). Apelin-36 exerts the cytoprotective effect against MPP+-induced cytotoxicity in SH-SY5Y cells through PI3K/Akt/mTOR autophagy pathway. Life Sci..

[422] Zhu J., Gao W., Shan X., Wang C., Wang H., Shao Z., Dou S., Jiang Y., Wang C., Cheng B. (2020). Apelin-36 mediates neuroprotective effects by regulating oxidative stress, autophagy and apoptosis in MPTP-induced Parkinson’s disease model mice. Brain Res..

[423] Satoh T., McKercher S.R., Lipton S.A. (2014). Reprint of: Nrf2/ARE-mediated antioxidant actions of pro-electrophilic drugs. Free Radic. Biol. Med..

[424] Skibinski G., Hwang V., Ando D.M., Daub A., Lee A.K., Ravisankar A., Modan S., Finucane M.M., Shaby B.A., Finkbeiner S. (2017). Nrf2 mitigates LRRK2- and α-synuclein–induced neurodegeneration by modulating proteostasis. Proc. Natl. Acad. Sci. USA.

[425] Mogana R., Teng-Jin K., Wiart C. (2013). Anti-Inflammatory, Anticholinesterase, and Antioxidant Potential of Scopoletin Isolated from Canarium patentinervium Miq. (Burseraceae Kunth). Evid. Based. Complement. Altern. Med..

[426] Narasimhan K.K.S., Jayakumar D., Velusamy P., Srinivasan A., Mohan T., Ravi D.B., Uthamaraman S., Sathyamoorthy Y.K., Rajasekaran N.S., Periandavan K. (2019). Morinda citrifolia and Its Active Principle Scopoletin Mitigate Protein Aggregation and Neuronal Apoptosis through Augmenting the DJ-1/Nrf2/ARE Signaling Pathway. Oxidative Med. Cell. Longev..

[427] Lee J.A., Son H.J., Choi J.W., Kim J., Han S.H., Shin N., Kim J.H., Kim S.J., Heo J.Y., Kim D.J. (2018). Activation of the Nrf2 signaling pathway and neuroprotection of nigral dopaminergic neurons by a novel synthetic compound KMS99220. Neurochem. Int..

[428] Lowe R., Pountney D.L., Jensen P.H., Gai W.P., Voelcker N.H. (2004). Calcium(II) selectively induces alpha-synuclein annular oligomers via interaction with the C-terminal domain. Protein Sci..

[429] Rasia R.M., Bertoncini C.W., Marsh D., Hoyer W., Cherny D., Zweckstetter M., Griesinger C., Jovin T.M., Fernandez C.O. (2005). Structural characterization of copper(II) binding to alpha-synuclein: Insights into the bioinorganic chemistry of Parkinson’s disease. Proc. Natl. Acad. Sci. USA.

[430] Binolfi A., Rasia R.M., Bertoncini C.W., Ceolin M., Zweckstetter M., Griesinger C., Jovin T.M., Fernandez C.O. (2006). Interaction of alpha-synuclein with divalent metal ions reveals key differences: A link between structure, binding specificity and fibrillation enhancement. J. Am. Chem. Soc..

[431] Jia F., Song N., Wang W., Du X., Chi Y., Jiang H. (2018). High Dietary Iron Supplement Induces the Nigrostriatal Dopaminergic Neurons Lesion in Transgenic Mice Expressing Mutant A53T Human Alpha-Synuclein. Front. Aging Neurosci..

[432] Jiang H., Song N., Jiao Q., Shi L., Du X. (2019). Iron Pathophysiology in Parkinson Diseases. Adv. Exp. Med. Biol..

[433] Wang R., Wang Y., Qu L., Chen B., Jiang H., Song N., Xie J. (2019). Iron-induced oxidative stress contributes to alpha-synuclein phosphorylation and up-regulation via polo-like kinase 2 and casein kinase 2. Neurochem. Int..

[434] Devos D., Moreau C., Devedjian J.C., Kluza J., Petrault M., Laloux C., Jonneaux A., Ryckewaert G., Garcon G., Rouaix N. (2014). Targeting chelatable iron as a therapeutic modality in Parkinson’s disease. Antioxid. Redox. Signal..

[435] Carboni E., Tatenhorst L., Tonges L., Barski E., Dambeck V., Bahr M., Lingor P. (2017). Deferiprone Rescues Behavioral Deficits Induced by Mild Iron Exposure in a Mouse Model of Alpha-Synuclein Aggregation. Neuromol. Med..

[436] Martin-Bastida A., Ward R.J., Newbould R., Piccini P., Sharp D., Kabba C., Patel M.C., Spino M., Connelly J., Tricta F. (2017). Brain iron chelation by deferiprone in a phase 2 randomised double-blinded placebo controlled clinical trial in Parkinson’s disease. Sci. Rep..

[437] Febbraro F., Andersen K.J., Sanchez-Guajardo V., Tentillier N., Romero-Ramos M. (2013). Chronic intranasal deferoxamine ameliorates motor defects and pathology in the alpha-synuclein rAAV Parkinson’s model. Exp. Neurol..

[438] Kaur D., Yantiri F., Rajagopalan S., Kumar J., Mo J.Q., Boonplueang R., Viswanath V., Jacobs R., Yang L., Beal M.F. (2003). Genetic or pharmacological iron chelation prevents MPTP-induced neurotoxicity in vivo: A novel therapy for Parkinson’s disease. Neuron.

[439] Finkelstein D.I., Hare D.J., Billings J.L., Sedjahtera A., Nurjono M., Arthofer E., George S., Culvenor J.G., Bush A.I., Adlard P.A. (2016). Clioquinol Improves Cognitive, Motor Function, and Microanatomy of the Alpha-Synuclein hA53T Transgenic Mice. ACS Chem. Neurosci..

[440] Billings J.L., Gordon S.L., Rawling T., Doble P.A., Bush A.I., Adlard P.A., Finkelstein D.I., Hare D.J. (2019). l-3,4-dihydroxyphenylalanine (l-DOPA) modulates brain iron, dopaminergic neurodegeneration and motor dysfunction in iron overload and mutant alpha-synuclein mouse models of Parkinson’s disease. J. Neurochem..

[441] Mena N.P., Garcia-Beltran O., Lourido F., Urrutia P.J., Mena R., Castro-Castillo V., Cassels B.K., Nunez M.T. (2015). The novel mitochondrial iron chelator 5-((methylamino)methyl)-8-hydroxyquinoline protects against mitochondrial-induced oxidative damage and neuronal death. Biochem. Biophys. Res. Commun..

[442] Shachar D.B., Kahana N., Kampel V., Warshawsky A., Youdim M.B. (2004). Neuroprotection by a novel brain permeable iron chelator, VK-28, against 6-hydroxydopamine lession in rats. Neuropharmacology.

[443] Zheng H., Weiner L.M., Bar-Am O., Epsztejn S., Cabantchik Z.I., Warshawsky A., Youdim M.B., Fridkin M. (2005). Design, synthesis, and evaluation of novel bifunctional iron-chelators as potential agents for neuroprotection in Alzheimer’s, Parkinson’s, and other neurodegenerative diseases. Bioorg. Med. Chem..

[444] Das B., Kandegedara A., Xu L., Antonio T., Stemmler T., Reith M.E.A., Dutta A.K. (2017). A Novel Iron(II) Preferring Dopamine Agonist Chelator as Potential Symptomatic and Neuroprotective Therapeutic Agent for Parkinson’s Disease. ACS Chem. Neurosci..

[445] Das B., Rajagopalan S., Joshi G.S., Xu L., Luo D., Andersen J.K., Todi S.V., Dutta A.K. (2017). A novel iron (II) preferring dopamine agonist chelator D-607 significantly suppresses alpha-syn- and MPTP-induced toxicities in vivo. Neuropharmacology.

[446] Finkelstein D.I., Billings J.L., Adlard P.A., Ayton S., Sedjahtera A., Masters C.L., Wilkins S., Shackleford D.M., Charman S.A., Bal W. (2017). The novel compound PBT434 prevents iron mediated neurodegeneration and alpha-synuclein toxicity in multiple models of Parkinson’s disease. Acta Neuropathol. Commun..

[447] Du T., Li L., Song N., Xie J., Jiang H. (2010). Rosmarinic acid antagonized 1-methyl-4-phenylpyridinium (MPP+)-induced neurotoxicity in MES23.5 dopaminergic cells. Int. J. Toxicol..

[448] Qu L., Xu H., Jia W., Jiang H., Xie J. (2019). Rosmarinic acid protects against MPTP-induced toxicity and inhibits iron-induced alpha-synuclein aggregation. Neuropharmacology.

[449] Zhang P., Park H.J., Zhang J., Junn E., Andrews R.J., Velagapudi S.P., Abegg D., Vishnu K., Costales M.G., Childs-Disney J.L. (2020). Translation of the intrinsically disordered protein alpha-synuclein is inhibited by a small molecule targeting its structured mRNA. Proc. Natl. Acad. Sci. USA.

[450] Villar-Pique A., Rossetti G., Ventura S., Carloni P., Fernandez C.O., Outeiro T.F. (2017). Copper(II) and the pathological H50Q alpha-synuclein mutant: Environment meets genetics. Commun. Integr. Biol..

[451] Castillo-Gonzalez J.A., Loera-Arias M.J., Saucedo-Cardenas O., Montes-de-Oca-Luna R., Garcia-Garcia A., Rodriguez-Rocha H. (2017). Phosphorylated alpha-Synuclein-Copper Complex Formation in the Pathogenesis of Parkinson’s Disease. Parkinsons Dis..

[452] Tsunemi T., Hamada K., Krainc D. (2014). ATP13A2/PARK9 regulates secretion of exosomes and alpha-synuclein. J. Neurosci..

[453] Tsunemi T., Krainc D. (2014). Zn(2)(+) dyshomeostasis caused by loss of ATP13A2/PARK9 leads to lysosomal dysfunction and alpha-synuclein accumulation. Hum. Mol. Genet..

[454] Kumar V., Singh D., Singh B.K., Singh S., Mittra N., Jha R.R., Patel D.K., Singh C. (2018). Alpha-synuclein aggregation, Ubiquitin proteasome system impairment, and L-Dopa response in zinc-induced Parkinsonism: Resemblance to sporadic Parkinson’s disease. Mol. Cell Biochem..

[455] Zhao J., Liang Q., Sun Q., Chen C., Xu L., Ding Y., Zhou P. (2017). (−)-Epigallocatechin-3-gallate (EGCG) inhibits fibrillation, disaggregates amyloid fibrils of α-synuclein, and protects PC12 cells against α-synuclein-induced toxicity. RSC Adv..

[456] Teng Y., Zhao J., Ding L., Ding Y., Zhou P. (2019). Complex of EGCG with Cu(II) Suppresses Amyloid Aggregation and Cu(II)-Induced Cytotoxicity of alpha-Synuclein. Molecules.

[457] Zhao J., Xu L., Liang Q., Sun Q., Chen C., Zhang Y., Ding Y., Zhou P. (2017). Metal chelator EGCG attenuates Fe(III)-induced conformational transition of alpha-synuclein and protects AS-PC12 cells against Fe(III)-induced death. J. Neurochem..

[458] Kurkowska-Jastrzebska I., Litwin T., Joniec I., Ciesielska A., Przybylkowski A., Czlonkowski A., Czlonkowska A. (2004). Dexamethasone protects against dopaminergic neurons damage in a mouse model of Parkinson’s disease. Int. Immunopharmacol..

[459] McLeary F.A., Rcom-H’cheo-Gauthier A.N., Kinder J., Goulding M., Khoo T.K., Mellick G.D., Chung R.S., Pountney D.L. (2018). Dexamethasone Inhibits Copper-Induced Alpha-Synuclein Aggregation by a Metallothionein-Dependent Mechanism. Neurotox Res..

[460] Bezard E. (2003). Neuroprotection for Parkinson’s disease: A call for clinically driven experimental design. Lancet. Neurol..

[461] Bezard E., Dovero S., Prunier C., Ravenscroft P., Chalon S., Guilloteau D., Crossman A.R., Bioulac B., Brotchie J.M., Gross C.E. (2001). Relationship between the appearance of symptoms and the level of nigrostriatal degeneration in a progressive 1-methyl-4-phenyl-1,2,3,6-tetrahydropyridine-lesioned macaque model of Parkinson’s disease. J. Neurosci..

[462] Kordower J.H., Olanow C.W., Dodiya H.B., Chu Y., Beach T.G., Adler C.H., Halliday G.M., Bartus R.T. (2013). Disease duration and the integrity of the nigrostriatal system in Parkinson’s disease. Brain.

[463] Davis A.A., Inman C.E., Wargel Z.M., Dube U., Freeberg B.M., Galluppi A., Haines J.N., Dhavale D.D., Miller R., Choudhury F.A. (2020). APOE genotype regulates pathology and disease progression in synucleinopathy. Sci. Transl. Med..

[464] Zhao N., Attrebi O.N., Ren Y., Qiao W., Sonustun B., Martens Y.A., Meneses A.D., Li F., Shue F., Zheng J. (2020). APOE4 exacerbates alpha-synuclein pathology and related toxicity independent of amyloid. Sci. Transl. Med..

